# The discovery of new metagenomic urethanases utilising a novel colorimetric assay for applications in the biodegradation of polyurethanes

**DOI:** 10.1039/d5gc03560k

**Published:** 2025-09-04

**Authors:** Silvia Anselmi, Yeke Ni, Alessia Tonoli, Jingyue Wu, Yu Wang, Luba Prout, Mark Miodownik, Jack W. E. Jeffries, Helen C. Hailes

**Affiliations:** a Department of Chemistry, University College London 20 Gordon Street London WC1H 0AJ UK h.c.hailes@ucl.ac.uk; b UCL Plastic Waste Innovation Hub, University College London 90 Tottenham Court Road W1T 4TJ London UK; c Department of Biochemical Engineering, University College London Bernard Katz Building Gower Street London WC1E 6BT UK; d Department of Mechanical Engineering, University College London Roberts Engineering Building Torrington Place London WC1E 7JE UK

## Abstract

The enzymatic molecular recycling of plastics is of increasing interest, where polymers are converted into monomers for reuse or upcycled into value added chemicals. Polyurethanes are an important class of synthetic hydrolysable polymers found in textiles as an elastane component, also known as lycra and spandex, with most post-consumer waste currently disposed of in landfill. Here we have identified three active novel urethane hydrolytic enzymes from a drain metagenome able to breakdown methylenedianiline-based elastane model substrates. In addition, we have established a new colorimetric assay, suitable for high-throughput applications using tyrosinases. For the urethanases identified, the reaction conditions and substrate scope were explored. Finally, the urethanases and assay were used with commercial fabrics, demonstrating breakdown of the polymer.

## Abstract 2

New urethane hydrolytic enzymes have been discovered from a drain metagenome and a novel colorimetric assay established for high-throughput applications.

## Graphical abstract

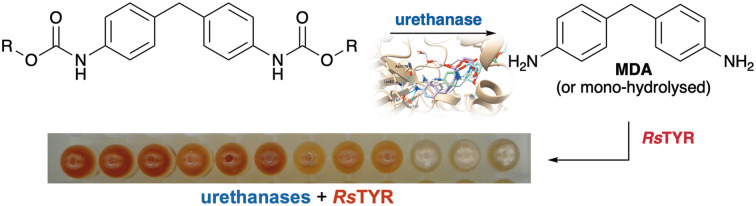

**Green foundation**

1. Polyurethanes (PUs) are a major class of synthetic hydrolysable polymers found in foams, adhesives, coatings and particularly textiles as an elastane component. New methods for depolymerising polymers are required to enable sustainable recycling and upcycling options for these valuable materials that currently mostly end up in landfill. Here, we have identified new enzymes to cleave the urethane bond in model compounds to enable the enzymatic depolymerisation of this synthetic fibre.

2. Initially a new colorimetric-based assay was developed and then used to screen enzymes identified from a drain metagenome. Three enzymes were highlighted for further study and reaction conditions explored, together with expansion of the substrate scope. Finally, the enzymes and assay were used with commercial fabrics as a proof of concept and combined with ball-milling and cellulases.

3. In the future further metagenome mining will be carried out using these enzyme sequences and enzyme engineering to enhance further the biodegradation properties.

## Introduction

Plastic waste is currently a major global issue. The production of plastics has increased by 9% on a yearly basis from the 1950s, with half of the total plastic produced manufactured within the last 20 years.^1–3^ As plastic production continues to increase, it is predicted that 12 billion tonnes will end up in landfill or the environment by 2050.^4,5^ The textile industry has seen a rapid growth in recent years, which has caused a substantial accumulation of textile waste, estimated at 92 million tons per year.^6,7^ Of this, 73% ends up in landfill, 14% is mismanaged and lost in the environment, 12% is downcycled to lower value products, and only 1% recycled.^6^ A major challenge with recycling textile waste is the complexity of the materials where natural and/or synthetic polymer fibres are woven into mixed fabrics. Textile recycling is then difficult due to the mixed materials and methods such as mechanical recycling results in lower quality materials. Enzymatic molecular recycling is of increasing interest, where polymers are converted into monomers for reuse or upcycled into value added chemicals.^8^ Such processes require less energy and compared to other recycling methods, use green solvents and sustainable reagents, providing a strategy towards a more sustainable circular economy.^8,9^

The biocatalytic depolymerisation of polyethylene terephthalate (PET) is a major example of successful biological recycling developments. Since the discovery of the PETase from *Ideonella sakaiensis* (*Is*PETase) in 2016,^10^ enzyme discovery and enzyme engineering has yielded more active and thermoresistant PETases.^11–17^ The work by Carbios with an engineered cutinase highlights potential applications of this strategy where postconsumer PET bottles were converted into monomers within 10 hours (h).^12^ Other work has developed mechanoenzymatic approaches with whole cell PETase enzymes and rPET-based textiles where up to 27-fold degradation improvements were compared to enzyme-lysate based reactions.^18^

**Fig. 1:**
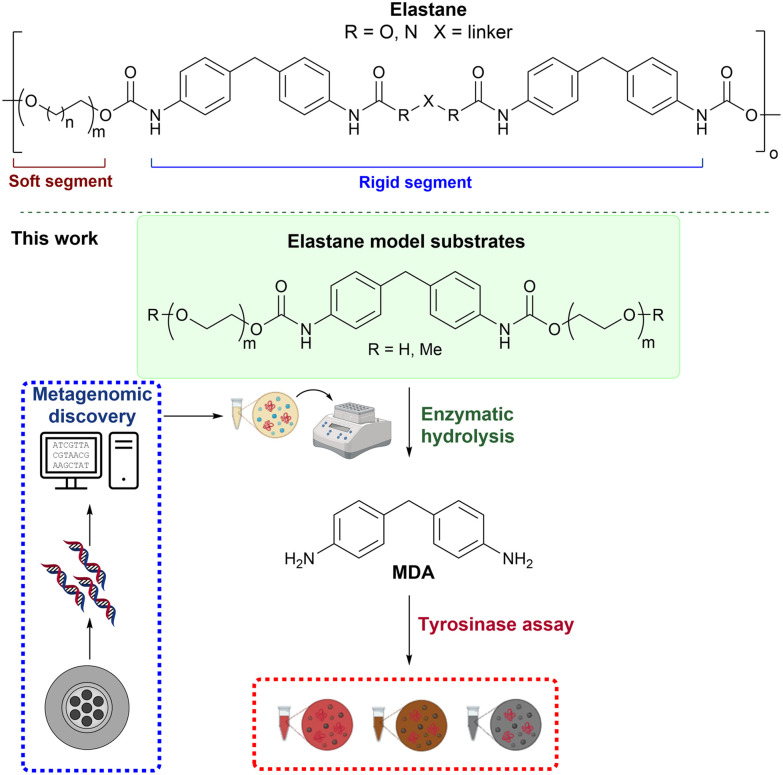
Representative structure of elastane and the approach adopted in this study for the metagenomic-driven discovery of novel urethanases using a colorimetric-based assay.

Polyurethanes (PUs) are another major class of synthetic hydrolysable polymers found in materials such as foams, adhesives and coatings. They are also prevalent in textiles as an elastane component (also known as lycra/spandex).^19^ The production of elastane is projected to grow by over 60% over the next decade, and most is currently disposed in landfill sites.^8^ There are currently no effective strategies to enzymatically depolymerise this synthetic fibre which would enable molecular recycling or upcycling options.^8,9^ Elastane has a rigid aromatic segment, typically comprised of 4,4′-methylenedianiline (MDA) and a soft polyether segment (or polyester segment) joined by a urethane bond (Fig. 1). The alternating rigid and soft segments make elastane both durable and elastic,^20^ and it is then blended with other fabrics including cotton (1–5%) and PET or nylon (up to 20%).

Esterases, lipases, cutinases, ureases and oxido-reductases, have been reported for the degradation of some polyurethanes.^9,20–25^ While the PUs used had rigid aliphatic or aromatic segments, the soft segments were polyesters, which are more susceptible to hydrolysis than carbamates. For example, Impranil-DLN (Covestro), an aliphatic polyester-polyurethane colloidal dispersion in water,^26^ was degraded by a strain of *Pseudomonas putida*.^27^ The vast majority of studies have described the enzymatic degradation of Impranil-DLN, although some polyurethane foams, films and model substrates have been used. Typical methods to study the enzymatic hydrolysis of PUs include mass loss, scanning electron microscopy (SEM), thermogravimetric analysis (TGA) and differential scanning calorimetry (DSC) for thermal properties, Fourier-transform infrared spectroscopy (FTIR) for functional group changes, and gel permeation chromatography (GPC) for oligomer formation.^25^ However, while indicating PU degradation, it is hard to determine whether the hydrolysis occurred at ester or carbamate sites. In 2023 Branson *et al.* reported for the first time urethanases UMG-SP-1-3 that can hydrolyse carbamates, and were active towards toluenediamine (TDA)-based urethane model substrates.^28,29^ Notably, UMG-SP-2 could hydrolyse >90% TDA-diethylene glycol (TDA-DEG) in 12 h and it was employed in the chemo-enzymatic recycling of TDA based foams, achieving the full hydrolysis of glycolyzed TDA-DEG breakdown products in 48 h. The same group later employed UMG-SP-2 in a one-pot two-step depolymerisation of an amorphous mix of hydrolysable plastics, namely PET, polybutylene adipate terephthalate (PBAT) and an MDA-based thermoplastic polyester polyurethane.^30^ Recent publications have also described mutagenesis studies.^31,32^ In 2025, further enzymes with urethanase activity, including a sequence from the AS family (OspAmd), have been identified.^33^

These reports are notable steps in identifying biocatalysts for the degradation of PUs, but it is clear that as an emerging field, new enzymes are required to tackle the sustainable degradation of elastanes. In parallel with recent reported studies, we have used a drain metagenome as the source of novel hydrolytic enzymes, active towards the breakdown of elastane model substrates (Fig. 1). To aid the discovery of suitable enzymes, a new colorimetric assay, suitable for high-throughput applications was established using tyrosinases (TYRs) and validated using reported urethanases. By using this assay to screen putative enzymes from the drain metagenome, three active biocatalysts were identified, and the reaction conditions and substrate scope explored. The urethanases and assays were then used with commercial fabrics. This work expands the biocatalytic toolbox and assays for the depolymerisation of elastane.

## Results and discussion

### Metagenome mining and identification of novel amidases

Metagenomics is the study of collective genomes from environmental samples and, as it is culture independent, it allows access to genomic material from unculturable organisms. In previous studies, our group mined various enzyme classes using sequence-based strategies applied to in-house metagenomic databases,^34–40^ in which all open reading frames (ORFs) were annotated according to the Pfam database. Specifically, the retrieval of transaminase,^35^ ene-reductase,^36^ transketolase,^38^ and deoxyribose-5-phosphate aldolase,^39^ sequences from the metagenome of a domestic drain led in some cases to the identification of hits with enhanced features, such as increased robustness and activity. In this work this metagenome was selected as a potential source of new amidase biocatalysts for two reasons: firstly, the metagenome was derived from a protein-rich environment, as materials such as hair and skin debris normally deposit in shower drains, and it was therefore predicted that it may have included a high number of amidases and similar hydrolytic enzymes; secondly, organisms growing in shower drains are exposed daily to soaps and other chemicals found in self-care products, thus representing a potential source of enzymes presenting high chemical resistance. From the literature, it was apparent that most enzymes capable of breaking down polyurethanes were either esterases or amidases, with amidases active towards the hydrolysis of the urethane bond.^28,29^ Currently, amidases are classified in three distinct categories:^41^ (i) the amidase signature (AS) family, the largest of the three, whose enzymes have shown activity towards a broad spectrum of substrates, including aliphatic and aromatic amides, as well as α-hydroxyamides; (ii) the nitrilase superfamily, whose substrate scope is quite narrow and limited mostly to aliphatic amides;^42^ and (iii) the FmdA_AmdA family, a small group of enzymes that can hydrolyse short chain aliphatic amides. Based on pre-existing literature and enzyme classification, the metagenomic analysis began by searching for novel AS family as promising elastane degrading enzymes.

**Fig. 2:**
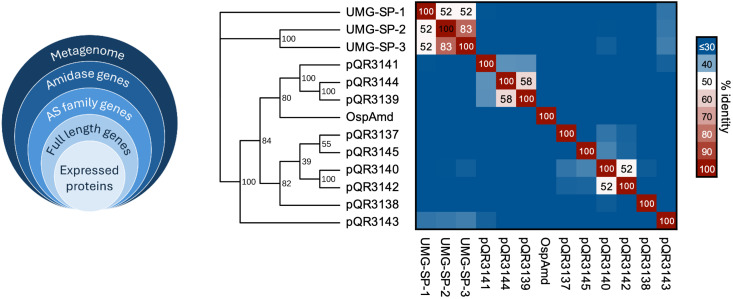
Newly identified amidases from the drain metagenome belonging to the AS family (left). Percentage of Identity Matrix and corresponding cladogram of retrieved amidases, including urethanases reported in the literature. The mutual identity among drain amidases ranges from 21% to 58%, with the highest identity to literature urethanases being 35% (pQR3143). Bootstrap support values are indicated at the nodes of the cladogram.

AS family enzymes, such as the amidase from *Rhodococcus* sp. (*Rh* amidase),^41^ the most studied amidase from this superfamily, present a highly conserved Ser–Ser-Lys catalytic triad, and can be identified by the Pfam identifier PF01425 (amidase domain). The lysine of the triad directly interacts with the OH of the second serine forcing the residue into an unusual *cis*-conformation.^41,43^ This Pfam ID was therefore used to retrieve all the ORFs of such amidases from the in-house drain metagenome. From a total of 46 identified ORFs, ten non-redundant sequences of at least 340 amino acids, complete with an initiator methionine and a stop codon were selected for PCR retrieval. Nine out of ten sequences were successfully amplified by PCR, cloned into the pET-29a(+) vector, and given pQR numbers 3137–3145 (Fig. 2). Based on a BLAST search, the amino acid sequences of the nine putative amidases showed 62–97% identity with known proteins in the NCBI database, all derived from bacteria of the Pseudomonadota group, the predominant phylum in this metagenome (Table S1).^44^ The multiple sequence alignment of the nine enzymes (Fig. S1) revealed sequence identities ranging from 21% to 58%, with most enzymes sharing 22–40% mutual identity (Fig. 2). Compared to the UMG-SP enzymes, pQR3143 exhibited the highest identity (34–35%), whereas the other metagenomic amidases showed lower identities, ranging from 23% to 32%. The enzyme pQR3145, the shortest candidate, did not present any catalytic lysine when aligned with the other putative amidases, but was still screened in assays. Phylogenetic analysis further suggested the separation between the UMG-SP urethanases and the retrieved enzymes, which form a distinct clade (Fig. 2). This divergence is also reflected by the InterPro families according to sequence analysis with the InterProScan tool:^45^ although all these amidases belong to the same signature superfamily, the UMG-SPs are classified within the more specific fatty-acid amide hydrolase family (IPR052739), while the majority of the enzymes in our set and the literature amidase OspAMD fall under the broader amidase family (IPR000120). Altogether this data indicated that the selected putative amidases pQR3137-3145 are distantly related and provide diverse potential candidates for the hydrolysis of MDA-based urethanes.

### MDA-urethanases colorimetric assay and validation

In parallel with the identification of new hydrolytic enzymes, an assay with potential in a high throughput setting was sought to detect the enzymatic breakdown of elastane or model substrates at the carbamate bond to release MDA (Fig. 1). While a coumarin based assay has been described for non-MDA carbamates,^31^ we were keen to detect MDA production to determine in screens whether an enzyme from a library should be investigated further. Tyrosinases (TYRs) are Cu-dependent enzymes that oxidises compounds such as tyrosine to melanins and other polyphenolic compounds.^46^ Monophenolase activity converts tyrosine to l-DOPA and the diphenolase activity oxidises this to dopaquinone which non-enzymatically polymerises to brown-black melanin. It has been reported that aromatic monoamines can be oxidised by TYRs to give initially *ortho*-aminophenols and then *ortho*-quinoneimines.^47,48^ If MDA could be oxidised by TYRs, it was envisaged that the quinoneimines formed could be tautomerised to generate conjugated compounds or polymers (Scheme S1) for use in a colorimetric assay.

**Fig. 3:**
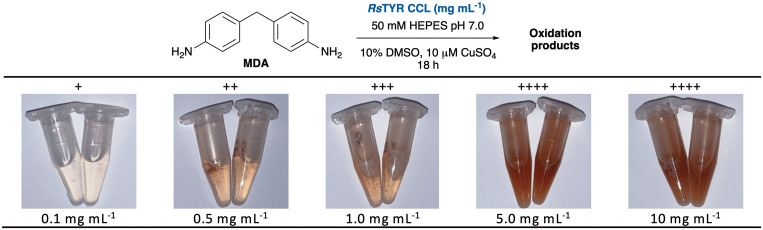
Development of the *Rs*TYR assay for the oxidation of MDA. Concentrations of the enzyme are expressed as total resuspended lyophilised CCL powder.

Available recombinant TYRs in *E. coli*, from *Ralstonia solanacearum* (*Rs*TYR), *Candidatus* Nitrosopumilus salaria BD31Q (*Cn*TYR) and a variant *Cn*TYR_N201S were used as crude cell lysates (CCL) in test assays.^49,50^ With dopamine, or MDA in 10% v/v of CH_3_CN or DMSO to enhance solubility, a black or dark red coloration was formed, respectively (Fig. S4 and S5). *Cn*TYR performed poorly in the presence of acetonitrile, suggesting poor stability in the presence of this solvent. In general, *Rs*TYR gave the clearest red coloration. Therefore, together with DMSO to solubilise substrates/fragments, *Rs*TYR was selected for further development of the assay. Different concentrations of resuspended lyophilised CCL (mg_LCCL_ mL^−1^) of *Rs*TYR (0.1 to 10 mg mL^−1^) were investigated. After 18 h, a concentration of 5.0 mg_LCCL_ mL^−1^*Rs*TYR, corresponding to 2.5 mg mL^−1^ total protein content, was sufficient to obtain a deep red colour (Fig. 3), and was then used further.

Quantification of the concentration of MDA oxidation products was attempted by spectrophotometric analysis. However, due to the poor solubility of the red products, it was not possible to obtain reproducible data at varying MDA concentrations. However, it is a valuable qualitative assay, suitable for high-throughput screening.

**Fig. 4:**
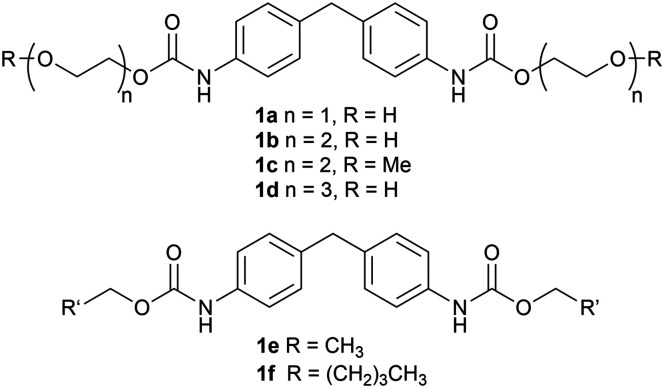
Elastane model substrates 1a–1f.

To identify urethanases in assays, model substrates were required. Several carbamates were synthesised using established methods, either from the isocyanate and ethylene glycol units (*n* = 1–3) including one with a terminal methoxy group (1a–1d),^51^ or from MDA and chloroformates^52^ to give two more hydrophobic analogues (1e, 1f) (Fig. 4).^53^ Carbamate 1e was previously described for use in initial screens with the UMG-SP enzymes,^29^ while 1b has been used in enzyme engineering studies^32^ in parallel with our enzyme discovery studies. To confirm that the carbamates (compared to MDA) did not give a positive TYR colorimetric readout, compound 1a was reacted with *Rs*TYR and after 18 h no colour change was observed (Fig. S6).

**Scheme 1:**
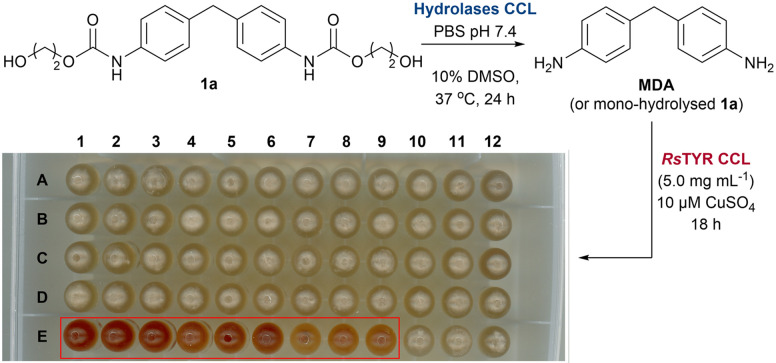
Screening of enzyme panel using 1a (also see SI, Table S3).

The colorimetric assay was then validated using a range of hydrolytic enzymes for comparison purposes including PETases,^10–18^ PLAses,^54^ nylonases (NylB and NylC),^55^ and the recently reported amidases which have shown activity towards urethanase bonds – UMG-SP-1-3^28^ (Table S3). The enzymes were used as CCL in a 96-well plate format with 1a. After 24 h reaction, *Rs*TYR was added and the reactions incubated for a further 18 h. From this initial screening, nine wells (three enzymes) turned red/orange, indicating the presence of hydrolysed products, potentially MDA or mono-hydrolysed 1a. These wells (E1–3, E4–6 and E7–8) contained UMG-SP-1, UMG-SP-2 and UMG-SP-3, respectively, enzyme hits capable of hydrolysing 1a (Scheme 1). These experiments were key and demonstrated the value of such a qualitative assay for initial hit identification.

To confirm the hits from the enzyme panel, HPLC analysis was performed. As predicted from the colorimetric assay, reactions in wells A1 to D12, as well as the empty vector controls (EVCs), showed no conversion to MDA, while reactions containing UMG-SPs showed good to poor conversions (Table 1). The conversion of **1a** by UMG-SP-1 was achieved in 83%, whereas UMG-SP-2 and UMG-SP-3 gave <10% conversions.

**Table 1:** Quantification of the conversions of 1a using reported UMG-SP enzymes under different reaction conditions and 1b–d with the EVC^a^

Enzyme^b^	Substrate	Buffer	Conv.^c^ %
UMG-SP-1	1a	PBS	83
UMG-SP-2	1a	PBS	8
UMG-SP-3	1a	PBS	5
UMG-SP-1^d^	1a	PBS	99
UMG-SP-2^d^	1a	PBS	46
UMG-SP-3^d^	1a	PBS	11
UMG-SP-1	1a	KPi	99^e^
UMG-SP-2	1a	KPi	54^e^
UMG-SP-3	1a	KPi	28^e^
EVC^f^	1a	KPi	2
EVC^f^	1b	KPi	19
EVC^f^	1c	KPi	<1
EVC^f^	1d	KPi	10

a Reactions were carried out in duplicate unless stated otherwise.

b CCL enzymes used unless indicated otherwise at a total protein concentration 10 mg mL^−1^, containing 7–10% urethanase enzyme.

c Conversions were based on the remaining 1a–d in solution, which was determined by HPLC analysis against product standards.

d Purified enzyme, 1.0 mg mL^−1^.

e Reactions were carried out in triplicate and the standard deviation was within 5% of the mean conversions given.

f EVC = BL21(DE3) empty pET-28a(+) vector.

To better understand the behaviour and reactivity of the reported enzymes against the model carbamates prior to screening the metagenomic enzymes, lyophilised CCL and the purified UMG enzymes were also tested. When stored as lyophilised CCL, UMG-SP-1 and 2 appeared to lose hydrolytic activity, indicating inactivation during the freeze-drying process. As purified enzymes, a quantitative hydrolysis of 1a was reached with UMG-SP-1, while it improved to 46% with UMG-SP-2; little change was observed in the conversion with UMG-SP-3 (Table 1). Further studies revealed that a switch in the reaction buffer from PBS to KPi gave further improvements when using CCLs (Table 1). Interestingly, a 2% conversion of 1a was observed in the negative control under these conditions, possibly due to background hydrolytic activity by *E. coli* endogenous enzymes. Substrates 1b-d were then reacted with UMG-SP-1-3 in KPi buffer. While all the enzymes converted 1b–1d in ∼99% conversions, most likely reflecting the improved substrate solubilities with the longer PEG side chains, for 1b and 1d, 19% and 10% conversions in the EVC control reactions were observed, respectively (Table 1). As no EVC conversion was observed for 1c, it was considered that the terminal hydroxyl group (in 1a,b,d) could potentially react intramolecularly with the carbamate moiety to some degree in assays.

### Initial screening of the 9 metagenomic amidases

**Scheme 2:**
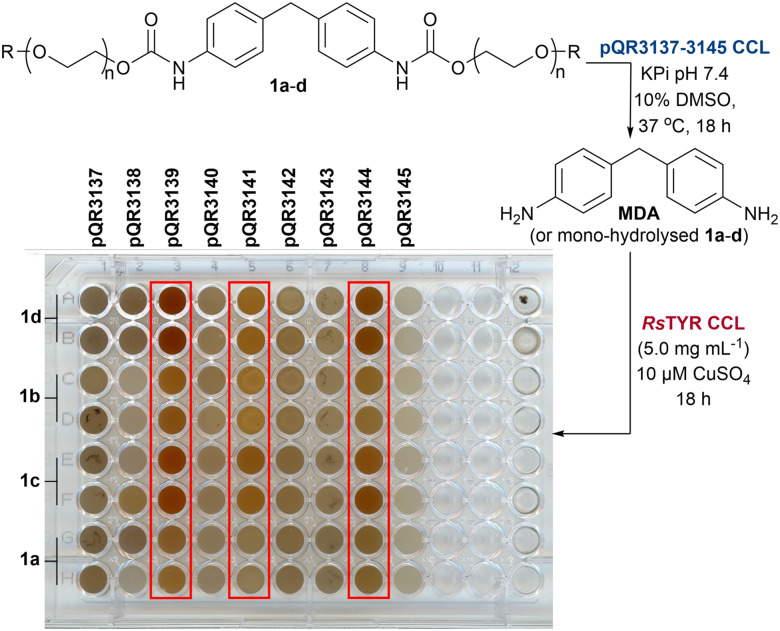
Screening of new amidases from the drain metagenome. Colorimetric assay shows the greatest activity with enzymes from pQR3139/3141/3144.

**Fig. 5:**
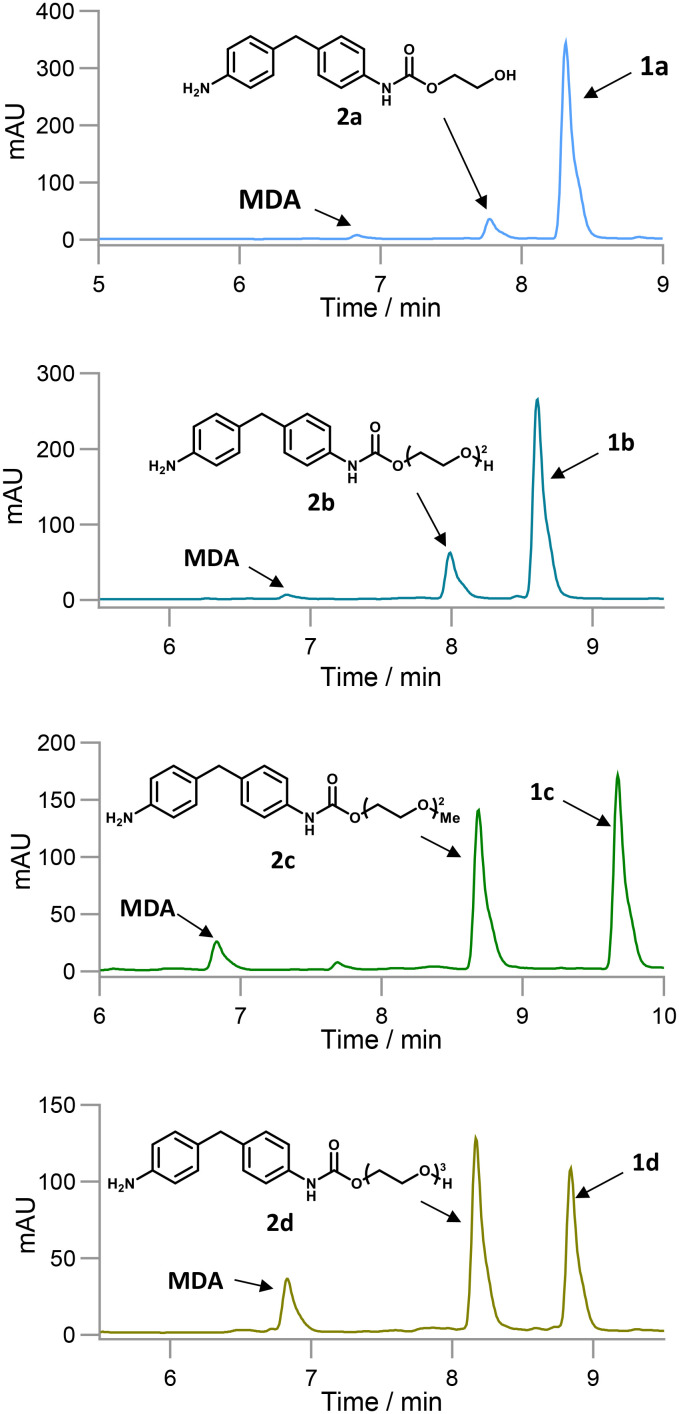
HPLC traces of the hydrolysis of substrates 1a–d using pQR3139 showing the formation of both MDA and the mono-hydrolysed products 2a–d.

After cloning the putative amidases in pET-29a(+) and expressing them in *E. coli* BL21(DE3) cells, these were obtained as CCL (Fig. S2) in 96-well deep-well plates and screened against 1a–d to identify active biocatalysts. After 18 h as before, *Rs*TYR was added to obtain a colorimetric readout. Enzymes pQR3139, pQR3141, and pQR3144 presented a red colour, suggesting urethanase activity (Scheme 2). Interestingly, all three enzymes showed increased activity from substrate 1a to 1d, as the red colour intensified progressively, indicating a preference for compounds with longer PEG chains. To confirm the activities, pQR3139, pQR3141, and pQR3144 were then grown on a 50 mL batch scale using either TB media or MagicMedia®, which had enhanced protein expression and solubility in previous work.^18^ While protein overexpression was achieved, enzyme solubility was still an issue (Fig. S3). Satisfactory protein yields were finally achieved when either LB or M9 minimal media were used, and protein production induced with 0.5 mM IPTG at an OD_600_ of 0.4. Hydrolysis of 1a–d using the amidases from pQR3139, pQR3141, and pQR3144 was then determined by HPLC analysis, monitoring the consumption of substrates, and the production of MDA against product standards (Table 2). While the concentration of MDA after 24 h was quite low, the starting materials were significantly reduced in most cases. The possibility of background hydrolysis was ruled out as being a major issue from previous experiments (Table 2). Further inspection of the HPLC traces, suggested the formation of intermediate mono-hydrolysed products (Fig. 5) which was confirmed by LC-MS analysis (see SI for spectra). Overall, substrates 1c, 1d were hydrolysed to 2c, 2d and MDA with 55–69% conversions, while 1a, 1b presented lower conversions of up to 38% and minimal MDA production. These results are in accordance with the colorimetric assay, indicating it can also detect mono-hydrolysed products, and highlighting its value for the identification of amidases active towards MDA or potentially other aniline-based urethanes. Substrates 1c and 1d were better accepted by the enzymes, and hence they were taken forward for further investigations.

**Table 2:** Hydrolysis of substrates 1a–d to MDA and 2a–d using amidases from pQR3139, pQR3141, pQR3144^a^

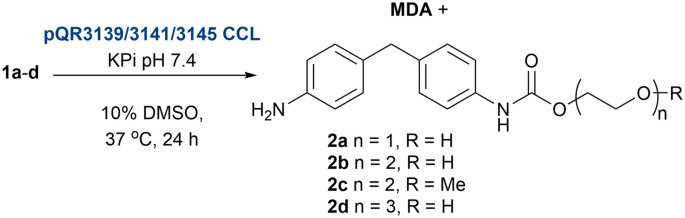			
Enzyme^b^	Substrate	Conv.^c^ %	MDA yield^d^ %
pQR3139	1a	9	3
pQR3139	1b	29	4
pQR3139	1c	58	13
pQR3139	1d	69	17
pQR3141	1a	6	<1
pQR3141	1b	38	<1
pQR3141	1c	55	1
pQR3141	1d	49	1
pQR3144	1a	25	2
pQR3144	1b	28	2
pQR3144	1c	63	14
pQR3144	1d	69	15

a Reactions were carried out in triplicate.

b CCL enzymes were used at a total protein concentration 10 mg mL^−1^, with a calculated amidase content of 10%.

c Conversions were based on the remaining 1a–d concentration in solution.

d HPLC yield against an MDA product standard.

### Optimization of the reaction condition of selected enzymes and expansion of the scope

**Fig. 6:**
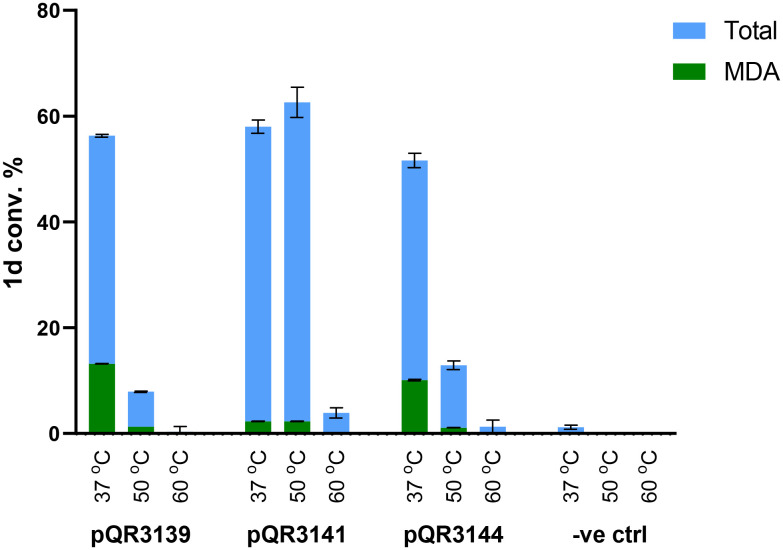
Conversion of 1d at 37, 50 and 60 °C using pQR3139, 3141, and 3144. Purified enzymes were used at a concentration of 0.5 mg mL^−1^. The −ve control was 50 mM KPi buffer pH 7.4. Reactions were carried out in duplicate (and the mean values are shown with error bars representing the standard deviation).

The thermostability of the amidases from pQR3139, pQR3141, and pQR3144 was explored at higher temperature (50 °C and 60 °C) using purified enzymes (at 0.5 mg mL^−1^). pQR3139 and pQR3144 appeared to have poor reactivities above 37 °C, while pQR3141 at 50 °C achieved a conversion to 1d and MDA of 63% compared to 58% at 37 °C (Fig. 6).

**Fig. 7:**
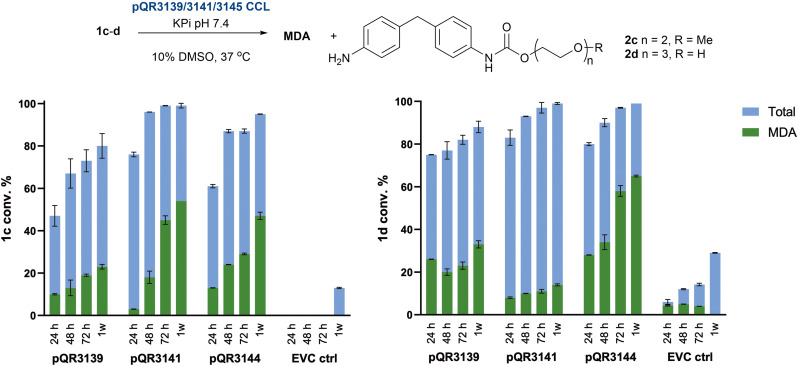
Time-point reactions of 1cd with pQR3139, 3141, and 3144. Reactions were carried out for 24, 48 and 72 h and 1 week in duplicate using the enzymes as CCL at a total protein concentration 20 mg mL^−1^, with an enzyme content of 10%. EVC controls = CCL BL21(DE3) empty pET-28a(+) vector. The mean values are shown with error bars representing the standard deviation.

Despite the slight improvement, further reactions were performed at 37 °C which is preferable on sustainability grounds. Nevertheless, the higher temperature tolerance could be useful in industrial processes. Interestingly, when pQR3139 and pQR3144 were used as purified enzymes at 37 °C, the total conversions of 1d were lower than when used as CCLs (Table 2). It was therefore decided to continue using all three amidases as CCLs. Longer reactions were then performed to evaluate the hydrolysis of substrates 1c and 1d at 24, 48, 72 h and 1 week. As shown in Fig. 7, all amidases retained activity over the course of a week, continuing to hydrolyse the substrates and leading to progressively higher 2c, 2d and/or MDA yields. In general, substrate 1d gave higher levels of hydrolytic products compared to 1c, presenting quantitative conversions after a week when reacted with pQR3141 and pQR3144. However, the intramolecular carbamate hydrolysis may have also occurred with 1d, highlighting 1c as a preferred test substrate.

**Fig. 8:**
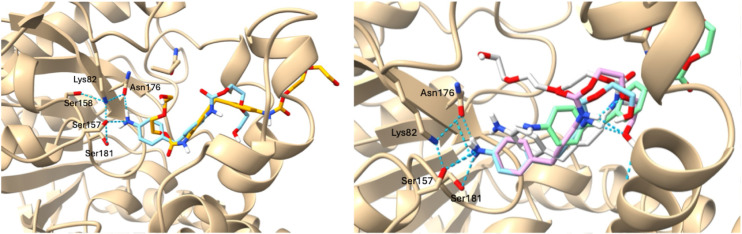
(A) *In silico* molecular docking (structure generated using Alphafold2,^56^ see SI) of 1b (orange, Δ*G* = −6.5 kcal mol^−1^) and 2b (cyan, Δ*G* = −7.0 kcal mol^−1^) with pQR3139, highlighting the binding of 2b to Ser, Lys and Asn residues. (B) *In silico* molecular docking of 2a–2d (2a cyan, 2b pink, 2c grey, 2d green) of pQR3139 highlighting the preference for 2a and 2b to bind further into the active site and slowing down the rearrangement required for the second hydrolysis step to release MDA.

These two enzymes performed better compared to pQR3139, leading to higher total conversions of the substrates. Conversions to MDA continued to increase with time for all combinations of substrates and enzymes with the highest MDA yield obtained with 1d and pQR3144 after 1 week (65%.) The production of 2d when using the model substrate 1d with the pendant hydroxyl group, also suggested some background hydrolytic cleavage. This was not observed when substrate 1c, with the capped methoxy group, was used. The data also indicated that the second hydrolysis of the mono-hydrolysed 2c–d was slower compared to the first hydrolytic reaction of 1c–d. This preference for the formation of the mono-hydrolysed products is interesting and comparable to hydrolysis of PET or bis(2-hydroxyethyl) terephthalate (BHET) to mono-(2-hydroxyethyl) terephthalate (MHET). It likely reflects different affinities for the less polar substrates 1 in the active site compared to 2 containing the pendant aniline group. Indeed, docking studies conducted with pQR3139 (structure generated using Alphafold2^56^) revealed that for example, substrate 1b hydrogen bonded less readily with the polar residues in the active site compared to 2b (Fig. 8A) where rearrangement in 2b is required to carry out the second hydrolysis to MDA. Indeed, the mono-hydrolysed products 2a and 2b formed strong hydrogen-bonding interactions with key catalytic residues within the active site *in silico*, with 2c and 2d further removed from the catalytic residues, enabling substrate release and rearrangement (Fig. 8B).

**Fig. 9:**
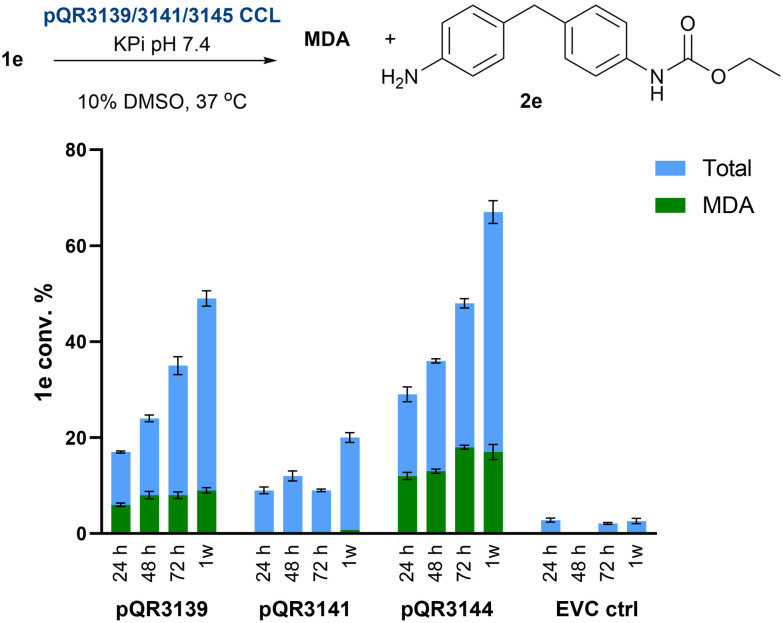
Conversion of 1e after 24, 48 and 72 h and 1 week using pQR3139, pQR3141, and pQR3144 as CCL. Total protein concentration 20 mg mL^−1^, with an enzyme content of 10%. Reactions carried out in duplicate (and the mean values are shown with error bars representing the standard deviation). EVC controls = CCL BL21(DE3) empty pET-28a(+) vector.

Although substrates 1a–d reflect the repeating units of elastane, the flexible and polar nature of the PEG side chains made these substrates quite water-soluble, particularly as the number of repeating ethylene units increased. However, one of the major challenges faced when trying to enzymatically degrade synthetic polymers is the insolubility of the material in the aqueous reaction conditions. Therefore, to assess the ability of pQR3139, pQR3141, and pQR3144 to catalyse the hydrolysis of more insoluble materials, model substrates 1e and 1f bearing aliphatic side chains were used. These appeared to be insoluble in 50 mM KPi buffer and 10% DMSO. When reacted with the three amidases as CCLs the reactions were again analysed at 24, 48 and 72 h and 1 week. To a lesser extent, the three amidases appeared to be active towards 1e, with a maximum total conversion of 67% achieved with pQR3144 after one week (Fig. 9).

In general, the same reactivity pattern as before was noted, where the yield of both MDA and the mono-hydrolysed product 2e increased during the week-long experiment. However, compared to 1c–d, pQR3141 was less active with 1e. While it was possible to calculate the hydrolytic conversion of 1e, the longer side chains in 1f rendered the substrate completely insoluble in any solvent/buffer combination.

**Fig. 10:**
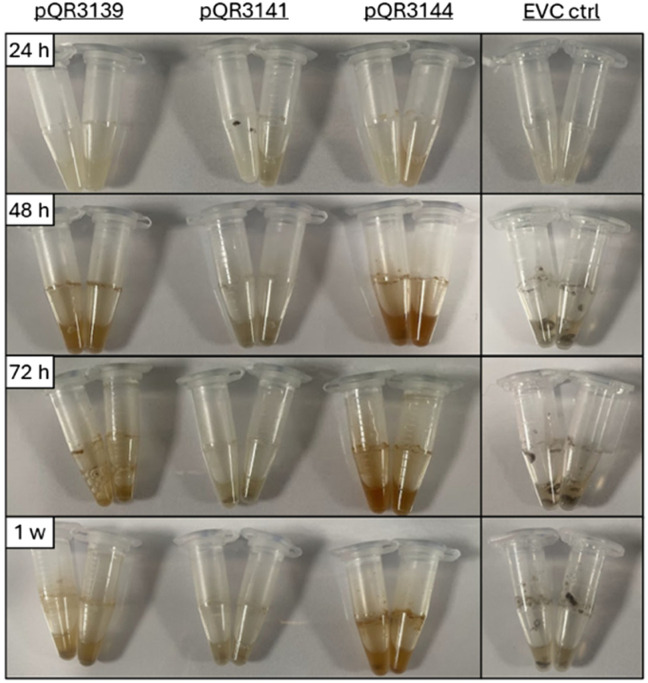
Qualitative analysis of time-point experiments for the hydrolysis of 1f using the *Rs*TYR colorimetric assay to assess the activity of pQR3139, pQR3141, and pQR3144.

Therefore, the *Rs*TYR qualitative assay was used instead to evaluate the activity of pQR3139, pQR3141, and pQR3144 toward 1f. Interestingly, very little red colour was observed after a 24 h reaction, indicating that there was no significant hydrolysis of 1f. Moreover, no red colouration was detected for pQR3141 (and EVC controls) (Fig. 10) in agreement with the much lower conversions obtained for 1e. However, a clear reddish colouration was noted with pQR3139 and pQR3144, highlighting the value of the colorimetric assay with substrates that are otherwise challenging to analyse by standard methods such as HPLC.

Following this approach, these enzymes were then used with two commercial textile samples containing 95% viscose/5% elastane, and 96% cotton/4% elastane according to the supplier's details provided. To minimise interference in assays from surface additives, they were first washed. Given that these fabrics are typically produced by twisting and blending different fibres, which can limit access to embedded elastic fibres, a bead-milling treatment was then used, as it was found to be effective in our previous studies.^18^ It was noted that the addition of methanol (1 mL per 250 mg fabric) during bead-milling enhanced the breakdown of the fibres. The resulting methanol solution and solid residue (resuspended in DMSO) were then treated with the amidases (CCL), followed by the TYR-assay to give a colorimetric readout of the degradation.

**Fig. 11:**
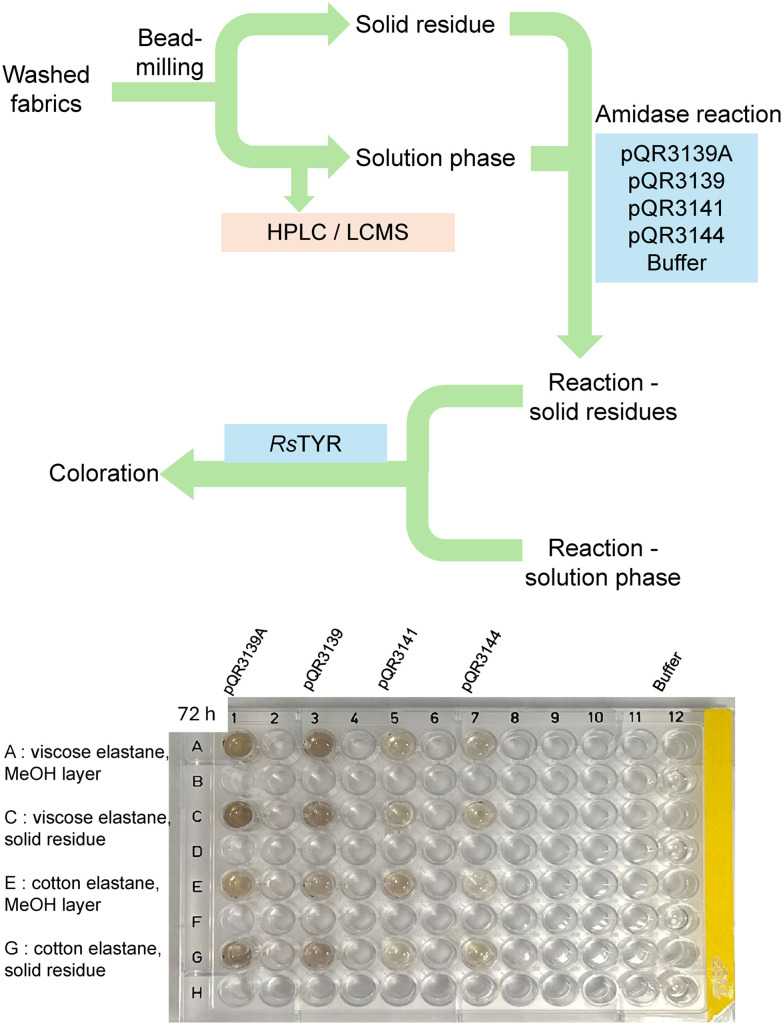
The general workflow for fabrics degradation and 96-well plates after 72 h showing the results of the amidase reactions and subsequent TYR-based assay. *Washing*: water and MeOH; bead-milling: 250 mg fabric per 1 mL MeOH; *Amidase reaction*: 10 mg materials in 100 μL DMSO, 100 μL amidases (as CCLs), 800 μL KPi buffer (50 mM, pH 7.5), 37 °C, 24–72 h Reactions for pQR3139 were carried out in duplicate. *TYR reaction*: 160 μL post amidase reaction, 20 μL *Rs*TYR, 20 μL CuSO_4_, 37 °C, 24 h. See SI for further details.

Negative controls were conducted both with buffer alone (Fig. 11) and with a pET-29a(+) empty vector (Fig. S7) with no coloration resulting. Notably, a colour change was observed within 1 h when using pQR3139 with both materials. It should also be noted that after the bead milling, samples were tested for urethane degradation with *Rs*TYR but no coloration resulted (Fig. 11, lane 12, see SI for further details).

**Fig. 12:**
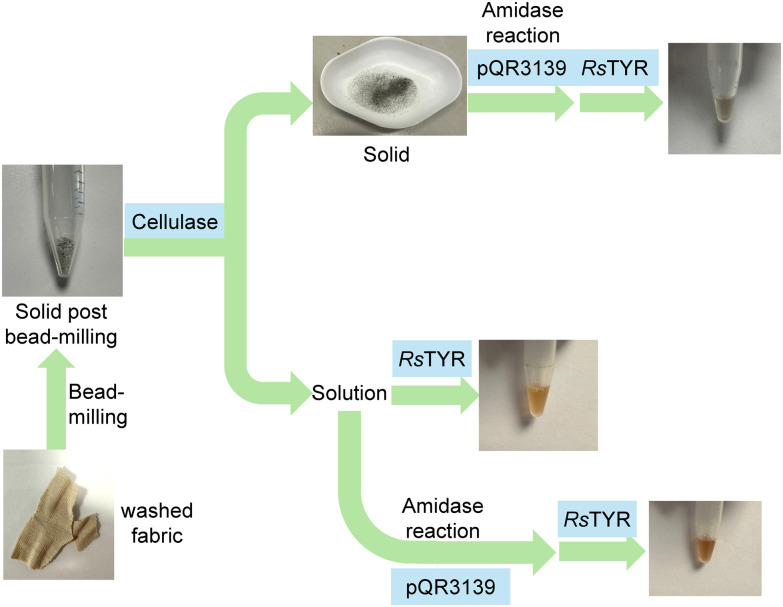
Viscose/elastane fabric treatment with bead-milling, cellulase, amidase and the TYR assay. *Bead-milling*: 250 mg fabric, 1 mL MeOH; *Cellulase reactions*: 100 mg fabric residue from bead-milling, 1 mL cellulase mix (Sigma-Aldrich), 9 mL KPi buffer (50 mM, pH 7.5), 50 °C, 1 h; *Amidase reactions*: 10 mg materials in 100 μL DMSO, 100 μL amidases, 800 μL KPi buffer (50 mM, pH 7.5), 37 °C, 24–72 h; *TYR reactions*: 160 μL amidase reactions, 20 μL *Rs*TYR, 20 μL CuSO_4_, 37 °C, 24 h.

From this initial assay, pQR139 was further used with the viscose/elastane fabric. Viscose cellulosic fibres can be hydrolysed into sugars using cellulases and this approach has recently been described for some textile polyester/polyamide blends.^57–59^ Therefore, after washing and bead-milling the fabric, it was first treated with a commercial cellulase mix to enhance access to the elastane. Then, pQR3139 was added to both the cellulase-treated solution and solid formed (Fig. 12).

Subsequent TYR assays with the cellulase-treated solution exhibited a pale red-orange coloration within 1 h, suggesting the presence of aniline/MDA fragments released from elastane by the cellulose. When the post-cellulase solution was further treated with pQR3139 followed by *Rs*TYR for the colorimetric readout, a darker coloration was observed, indicating breakdown of a polyurethane to give terminal aniline/MDA units. Notably, the solid arising from the cellulase reaction when treated with pQR3139 and *Rs*TYR gave a faint coloration.

It was not possible to obtain information on which elastane was present, as the structure of elastane can vary considerably and protected under patent. However, ^1^H NMR analysis (Fig. S8) revealed signals corresponding to aromatic rings and methylene bridges on MDA-derived fragments. This suggested that the amidases achieved the depolymerisation of elastane into fragments. Overall, this initial study with elastane containing fabrics, highlighted the value of the colorimetric assay for determining the enzymatic degradation of the elastane components. The treatment with cellulases was also useful to breakdown cellulose fibres into soluble sugars, thereby improving access to the elastane fibres.

## Conclusions

In conclusion, we have identified novel urethane hydrolytic enzymes from a drain metagenome able to breakdown elastane model substrates. In addition, we have established a new colorimetric assay suitable for high-throughput urethanase screening applications utilising tyrosinases. From this, three active urethanases were identified, and the reaction conditions and substrate scope explored. The urethanases and assays were then used with commercial fabrics which were treated with ball-milling. Incubation of these fabrics with a cellulase mix and the selected amidase pQR3139 enabled the depolymerisation of elastane components. This work highlights the potential of using enzymes to sustainably depolymerise textiles and future work will focus on expanding the pool of enzymes through further metagenome mining and enzyme engineering of the best performing amidases to enhance their polyurethane degradation properties.

## Author contributions

S. A. performed chemical syntheses, enzyme discovery and enzymatic assays. A. T. carried out the metagenomics analysis, Y. W. the initial tyrosinase work and L. P. helped with the enzyme discovery and cloning. S. A and J. W. performed the *in silico* docking. Y. N. performed chemical synthesis and bead milling/assay work. The manuscript first draft was written by S. A./H. C. H. and then through contributions of all authors. The project was conceived and supervised by M. M., J. W. E. J. and H. C. H. All authors have given approval to the final version of the manuscript.

## Conflicts of interest

There are no conflicts to declare.

## Supplementary Material

GC-027-D5GC03560K-s001

## Data Availability

The data supporting this article have been included as part of the SI. Supplementary information: Experimental details and NMR spectra. See DOI: https://doi.org/10.1039/d5gc03560k.
