# Supplementary material for: The discovery of new metagenomic urethanases utilising a novel colorimetric assay for applications in the biodegradation of polyurethanes
Source: Green Chem. 2025 Sep 4;27(39):12176–86. doi: 10.1039/d5gc03560k (PMC12421990; doi:10.1039/d5gc03560k)
Supplement: GC-027-D5GC03560K-s001 [file GC-027-D5GC03560K-s001.pdf]

Supplementary Information (SI) for Green Chemistry.
This journal is © The Royal Society of Chemistry 2025

The discovery of new metagenomic urethanases utilising a novel colorimetric assay for applications in the biodegradation of polyurethanes

Silvia Anselmi,[a,b] Yeke Ni,[a-c] Alessia Tonoli,[b,c] Jingyue Wu,[a,b] Yu Wang,[a] Luba Prout,[b,c] Mark Miodownik,[b,d] Jack W. E. Jeffries[b,c]* and Helen C. Hailes[a,b]*

[a] *Department of Chemistry, University College London, 20 Gordon Street, London, WC1H 0AJ, UK. E-mail: h.c.hailes@ucl.ac.uk.*
[b] *UCL Plastic Waste Innovation Hub, University College London, 90 Tottenham Court Road, W1T 4TJ, London, UK.*
[c] *Department of Biochemical Engineering, University College London, Bernard Katz Building, Gower Street, London, WC1E 6BT, UK.*
[d] *Department of Mechanical Engineering, University College London, Roberts Engineering Building, Torrington Place, London, WC1E 7JE, UK.*

Supporting information

Table of Contents

1 Molecular biology and bioinformatics 3

1.1 General methods 3

1.2 Proteins and DNA sequences of DM amidases 3

1.3 Protein and DNA sequences of UMG-SP amidases 8

1.4 Multiple sequence alignment (MSA) of putative amidases pQR3137-3145 and urethanases from the literature 11

1.5 Percentage of identity matrix and phylogenetic analysis 11

1.6 BLAST search results with percentage identity to known proteins 12

1.7 Metagenomic amidases expression and purification 12

1.8 SDS-PAGE gel of pQR3137-3145 13

1.9 List of tyrosinases and growth conditions 14

1.10 Tyrosinase studies 15

1.11 Proposed mechanism of tyrosinase-mediated oxidation of MDA 16

1.12 List of hydrolases screened with the HTS colorimetric method 16

1.13 Layout of Jeffries-Hailes library in 96-well plate used for the colorimetric assay 17

1.14 Procedure for the screening of enzyme panel with the colorimetric assay 17

1.15 Batch growth of urethanases 18

1.16 General procedure for the enzymatic hydrolysis of substrates 1 18

1.17 HPLC calibration curves 18

2 Chemistry 25

2.1 General methods 25

2.2 General synthesis of polyether model substrates 1a-d 25

2.3 Diethyl (methylenebis(4,1-phenylene))dicarbamate 1e[22] 26

2.4 Synthesis of dihexyl (methylenebis(4,1-phenylene))dicarbamate 1f[23] 27

2.5 NMR spectra 28

2.6 Mass spectrometry analysis of monohydrolysed compounds 2 34

3 Molecular docking 37

4 Experiments with commercial fabrics 39

4.1 General procedures 39

4.2 Investigations with commercial fabrics 39

5 References 41

1 Molecular biology and bioinformatics

1.1 General methods

Salts and media were used as supplied and according to vendor instructions. Milli Q water and RO water were obtained from Merk purifying system. Centrifuges used were Beckman Avanti JXN-26 Series, Eppendorf Centrifuge 5415R, Eppendorf Centrifuge 5810R, Eppendorf Centrifuge 5430R. Kuhner ShakerX ClimoShaker ISFI-X and New Brunswick Scientific Innova 44 incubating shakers were used for cell culture. Eppendorf ThermoMixer C and BIOER Mixing Block MB-102 thermomixers were used to incubate 96-well plate cultures and enzymatic reactions. ProFlex PCR System were used for cloning the metagenomic genes. The autoclaves used were a Priorclave TACTROL 2 and a Priorclave TACTROL 3. Where sterilisation of waste and media was performed, the temperature was held at 121 °C for 20 min. Sonication of *E. coli* cells was performed using a Branson Sonifier 150 with either a microprobe tip for volumes up to 15 mL or a probe tip for volumes up to 50 mL at 75% power (15 µA). Purification of clarified cell lysate (CCL) was performed manually by gravity or vacuum Ni Sepharose 6 Fast Flow resin from Cytiva. UV-Vis spectrometry was carried out using a Thermo Scientific NANODROP 2000c, UV-vis instrument JENWAY 7315 Spectrometer and plate reader CLARIOstar plus.

1.2 Proteins and DNA sequences of DM amidases

For the initial screening, all genes were cloned into pET-29a(+) vectors flanked with NdeI/XhoI restriction sites and with a C-terminal His_6-tag. Cloning was performed with traditional PCR methods from New England Biolabs according to the manufacturer instructions. For selected enzymes pQR3139, 3141 and 3144, the codon-optimised genes for expression in *E. coli* were also ordered from GenScript as synthetic genes already in pET-29a(+) with a C-terminal His_6-tag.

>pQR3137

MQKISLLGAAALLAISPAAAPVSAMPPPLPEVEGKDAATLRAEMESGMLYEGLIALVYQDRIARIDDYGPKLD
AVLATRSQADLSAEGKRLYDERMAGKARGPLHGIPILVKDNIEVAGLPTTAGSLALKGNMTNRDAPIIARLRK
AGAIILGKTNLSEWANIRSDNSTSGWSAVGGLTKNPHALDRNTCGSSSGSAAAVAASLAPLAIGTETDGSITC
PAGVNGIVGFKPTVGLVSRTHIVPISHTQDTAGPMTLTVRDAAAVMTVIAGSDPADPATAEADARKTDYIAAL
SPDALKGKRVGVLRDRIGGRADIAALLDAALKQMEGLGATVVEIADSRKGLEELGAAELEILLTELKADIKTY
LASLPDAKGPKSLADVIAFNKAHPDELKWFDQSLFELAETKGGLDSKTYLDAKAKAARLAGPEGIDRLLAAYK
VDLLIGVTNGPAWTSDLVNGDHYNGPSASQLPAVAGYPHLTVPMGTVQGLPIGISFVGPEWSDAEVLAAGYAY
EQASRKRMAPTFRASAAPLEHHHHHH*

ATGCAAAAAATTTCGCTGCTCGGCGCCGCCGCACTCCTCGCCATCTCCCCCGCCGCCGCCCCGGTCTCAGCAA
TGCCGCCGCCACTTCCCGAGGTCGAAGGCAAGGATGCAGCCACGCTTCGCGCCGAGATGGAAAGCGGCATGCT
CTACGAAGGGCTGATCGCGCTCGTCTATCAGGACCGGATCGCCCGGATCGACGATTATGGGCCCAAGCTCGAC
GCCGTGCTCGCAACCAGGTCCCAAGCCGACCTCAGTGCCGAGGGAAAGCGACTGTATGACGAGCGGATGGCGG
GCAAGGCGCGCGGCCCGCTCCATGGCATCCCGATCCTCGTCAAGGACAATATCGAGGTCGCCGGCCTGCCGAC
GACGGCGGGCTCGCTCGCGCTCAAGGGCAATATGACCAACCGCGATGCGCCGATCATAGCGCGGCTGCGCAAG
GCGGGCGCGATTATCCTTGGCAAGACGAACCTCAGCGAATGGGCGAACATCCGTTCGGACAATTCGACGAGCG
GGTGGAGCGCGGTCGGGGGGCTGACGAAAAACCCGCACGCGCTCGATCGCAATACATGTGGTTCCTCATCAGG
CAGCGCCGCTGCCGTCGCCGCCAGCCTTGCCCCGCTTGCCATCGGGACCGAAACCGACGGCTCGATCACCTGT
CCCGCCGGGGTCAACGGCATCGTCGGCTTCAAGCCCACCGTCGGGCTGGTCAGTCGCACACATATCGTGCCAA
TCAGCCATACGCAGGACACCGCAGGCCCGATGACGCTGACCGTCCGCGATGCCGCCGCGGTGATGACGGTGAT
CGCGGGCAGCGACCCCGCCGATCCTGCGACGGCCGAGGCTGATGCCCGCAAGACCGACTATATCGCCGCGCTC
TCGCCCGATGCGCTGAAGGGCAAGCGTGTCGGCGTACTGCGCGACCGGATCGGCGGTCGTGCCGATATCGCCG
CGCTACTCGATGCTGCGCTGAAGCAGATGGAAGGTCTCGGCGCGACAGTCGTCGAGATCGCCGACAGCCGCAA
AGGGCTGGAGGAACTGGGCGCCGCGGAACTCGAAATATTGCTGACCGAACTCAAGGCCGACATCAAAACCTAT
CTAGCCTCGCTGCCGGACGCCAAGGGGCCCAAATCGCTCGCCGACGTCATCGCCTTCAACAAGGCGCATCCCG
ACGAACTCAAATGGTTCGACCAGTCGCTGTTCGAACTCGCGGAGACCAAGGGCGGGCTCGACAGCAAGACCTA

TCTCGACGCCAAGGCCAAGGCTGCGCGGCTTGCGGGGCCCGAGGGCATCGACCGGTTGCTCGCGGCGTACAAG
GTCGACCTTCTCATCGGCGTCACCAACGGCCCTGCCTGGACCAGCGACCTCGTCAACGGCGATCATTACAACG
GCCCGAGCGCGAGCCAGCTCCCCGCTGTGGCCGGTTACCCGCACCTCACGGTGCCCATGGGCACGGTGCAGGG
CCTCCCGATCGGGATCAGCTTCGTCGGGCCCGAATGGAGCGATGCGGAGGTGCTCGCCGCGGGTTACGCCTAT
GAGCAAGCGAGCAGGAAGCGCATGGCGCCGACGTTTCGGGCAAGTGCCGCGCCC

>pQR3138

MSASSLWTIADWQHAYRTQGQSLEQLLAWHASLDANDPAWLHLLSAEHITAQLETLATRLVACGQDWTRLPLY
GIPFAVKDNIDVEGLPTTAACPMLQTPASSDAHVVKTLKALGAVVIGKTNLDQFATGLVGTRSPYGIVPNPFH
PAYISGGSSSGSASVVSRGIVPFALGTDTAGSGRVPAALQNIVGLKPTRGWFSNTGLLPACKTLDCISVFALT
VADAWLIAELMGGSDPLDPYSRVHPRTTPAAFSSTPKIGVPASLFFAEDPIQAAAFAVAQQQWIDLGATLVPI
DFSSFEALAKQLYQGAWVAERTAAVGELLSEPTSAHAVVKTIIDKGHLFSAIDAYNAEYQRAELSQQIAQTFA
AVDCILVPTTPTIYTIQALLEQPIELNSVLGTYTNFTNLADLSALAIPAPKRSDGLPFGVTLIAPAWYDRALA
ELAQRMQNQVGWILGTSNRPTPTQQLSISPNHVRVAVVGAHLTGMPLNFQLTTRQAVLVEQTLTAPQYALYAL
AGTVPPKPGLARCAEGEQGHNLIVEVWDIPTARFGEFVAEIPPPLGIGNVELIDGRWVKGFICEPYALASAQP
ISEFGGWRAYIAHRNAQAAAAKTTNLEHHHHHH*

ATGTCCGCCTCCTCTTTGTGGACGATTGCTGATTGGCAGCATGCGTACCGCACACAAGGTCAATCGCTTGAGC
AACTGTTGGCATGGCATGCCAGTTTAGATGCCAACGATCCAGCGTGGCTGCATCTGCTCAGTGCTGAGCACAT
CACCGCACAACTCGAAACCCTCGCCACGCGTTTGGTCGCGTGTGGGCAGGATTGGACGCGACTGCCCTTATAT
GGGATTCCTTTTGCGGTCAAAGACAATATTGATGTGGAAGGGCTACCCACCACCGCTGCCTGTCCGATGCTAC
AAACCCCCGCCAGTAGTGATGCTCATGTGGTCAAAACCCTCAAAGCTCTTGGTGCGGTGGTGATTGGTAAAAC
CAATTTGGATCAATTTGCCACCGGTCTGGTGGGCACGCGCTCACCGTATGGCATTGTCCCCAATCCATTTCAT
CCGGCTTATATCAGTGGAGGCTCCAGCTCCGGCTCGGCAAGCGTGGTCAGTCGTGGCATTGTGCCTTTTGCGC
TAGGCACCGATACCGCAGGTTCGGGTCGTGTGCCTGCGGCGCTGCAAAATATCGTGGGACTCAAGCCGACACG
CGGTTGGTTTTCCAATACTGGTTTGTTGCCTGCCTGTAAGACCCTTGATTGTATTTCGGTATTTGCGCTCACT
GTAGCGGATGCTTGGCTGATTGCCGAATTGATGGGCGGGTCAGATCCGCTTGATCCTTACTCACGTGTGCATC
CACGCACCACCCCTGCGGCATTTTCATCTACACCCAAAATCGGCGTTCCTGCGTCGCTGTTTTTTGCTGAAGA
TCCAATCCAAGCGGCGGCGTTTGCGGTGGCGCAACAACAGTGGATTGATCTGGGTGCAACACTGGTACCGATT
GATTTTTCCTCATTTGAAGCCCTTGCCAAACAACTGTATCAAGGTGCTTGGGTCGCAGAGCGTACCGCAGCAG
TGGGTGAACTGTTGTCTGAGCCAACTAGCGCCCATGCGGTGGTCAAAACTATTATTGATAAAGGTCACTTATT
TAGTGCCATCGATGCGTACAACGCCGAGTATCAACGTGCTGAGCTGAGCCAGCAGATCGCCCAAACCTTTGCT
GCGGTGGATTGTATCCTTGTACCGACCACCCCAACCATCTACACCATCCAAGCGTTGCTTGAGCAACCGATTG
AACTCAATAGTGTACTGGGGACATATACCAACTTTACCAATCTGGCTGACTTGTCAGCGCTTGCCATTCCCGC
GCCCAAACGCAGCGATGGCTTGCCGTTTGGGGTCACCTTGATTGCACCTGCATGGTATGACCGTGCGTTGGCT
GAACTGGCACAACGGATGCAAAATCAGGTGGGCTGGATACTCGGCACCTCAAACCGTCCCACCCCCACCCAAC
AATTGTCGATCAGCCCCAATCATGTCCGCGTGGCGGTGGTGGGTGCACACCTGACGGGTATGCCCCTCAACTT
CCAACTCACCACCCGCCAAGCAGTATTGGTCGAGCAAACCCTGACTGCACCGCAGTACGCCCTGTATGCCCTT
GCGGGCACAGTACCGCCCAAACCGGGTTTGGCACGCTGTGCAGAGGGTGAGCAAGGCCATAACCTGATTGTCG
AAGTGTGGGACATCCCCACCGCACGTTTTGGTGAATTTGTAGCAGAAATTCCGCCGCCACTTGGCATTGGCAA
TGTCGAATTGATCGATGGTCGCTGGGTCAAAGGCTTTATTTGCGAGCCTTATGCACTGGCGAGCGCCCAACCG
ATCAGTGAGTTTGGCGGCTGGCGTGCCTATATCGCCCATCGCAATGCCCAAGCCGCTGCCGCAAAAACAACCA
AT

>pQR3139

MTTHAIHAFTQDALGTDDAVALVQRLKRREVSATELVDAALRRAAQVNPALHAIAFLDEQHAKQHAKHASQGV
LAGIPSFIKDNTDVVGWPTRQGSRAVVARPATQHGAFAAQFLAQGLIVLGKSTLPEFGFNASTEPAFAAATAN
PWDTRYSAGGSSGGAAALVAAGVVPIAHANDGGGSIRIPAACCGLVGLKPTRGRTIPDDRTKILPINIVNDGV
LTRSVRDTALFFAGAERHWQNPKLPPIGWVTQPSPERRRIGVVVDSITGRPTDTDTRAAVEHTAVLLEQLGHQ
VELIPVPASPIFADDFNDYWAFLAFLVSTFGKVNFGREFDALHMDALSHGLVQRFRARGWRLPQVLYRLKQTE
AQARRLTAQYDAILSPVLAHTTPRLGHLTPEQTFDELMDKLRHYVSFTPLNNANGTPAISLPLGRTSQGLPIG
VQLSAAHGAERILLELALELEEASPWQQLYQQPLIALEHHHHHH*

ATGACGACGCACGCCATCCACGCTTTTACCCAAGATGCGCTTGGTACAGATGATGCTGTGGCGCTGGTGCAAC
GGCTCAAGCGCCGCGAGGTCAGTGCGACGGAGTTGGTCGATGCGGCACTGCGGCGTGCGGCACAGGTCAATCC
AGCCTTACATGCCATCGCTTTTTTGGATGAGCAGCATGCCAAACAACACGCCAAGCATGCCTCGCAAGGCGTA
TTGGCGGGGATTCCGAGTTTTATCAAAGACAATACCGATGTGGTGGGTTGGCCGACTCGACAAGGCTCACGCG
CTGTGGTGGCACGCCCTGCCACGCAGCATGGTGCATTTGCGGCACAGTTTTTGGCACAAGGCTTGATTGTATT
GGGCAAAAGCACCTTGCCTGAGTTTGGTTTTAATGCCTCGACCGAGCCGGCCTTTGCCGCAGCCACCGCCAAC
CCTTGGGATACACGCTATTCGGCGGGTGGCTCGTCGGGGGGGGCTGCGGCACTGGTCGCTGCGGGTGTCGTTC
CGATTGCCCATGCCAATGATGGCGGTGGGTCAATCCGCATACCGGCGGCCTGTTGTGGCTTGGTTGGACTCAA
ACCCACCCGTGGACGCACCATCCCCGATGATCGTACCAAGATCCTTCCCATCAATATTGTCAACGATGGCGTG
CTGACCCGCAGTGTGCGCGATACCGCGTTGTTTTTGCAGGGGCAGAGCGGCATTGGCAAAATCCCAAACTGC
CACCAATTGGCTGGGTGACACAGCCTAGTCCAGAGCGTCGTCGGATTGGCGTGGTGGTGGACTCTATTACAGG
GCGACCGACCGATACCGATACCCGTGCCGCCGTTGAGCATACTGCTGTCTTGTTGGAGCAGCTTGGACATCAG
GTTGAGTTGATTCCGGTGCCAGCGTCCCCGATTTTTGCCGATGATTTCAATGATTATTGGGCGTTTTTGGCCT
TTTTAGTCAGCACCTTTGGCAAAGTCAATTTTGGTCGTGAGTTTGACGCGTTGCACATGGATGCACTGAGTCA
TGGGTTGGTGCAACGCTTTCGGGCGCGTGGCTGGCGATTGCCGCAAGTCTTGTATCGCCTCAAACAAACGGAG
GCGCAAGCACGTCGCCTGACCGCGCAATACGATGCGATTTTGTCACCAGTGTTGGCGCATACCACACCGCGAC
TGGGGCATTTGACGCCAGAACAAACCTTTGATGAACTGATGGATAAACTGCGGCACTATGTTAGCTTTACGCC
GCTCAACAATGCCAATGGGACGCCCGCCATCTCGTTACCACTGGGGCGTACCTCACAAGGCTTACCCATAGGC
GTGCAATTGAGCGCAGCACATGGTGCAGAGCGCATCTTGCTGGAGCTGGCATTGGAGCTGGAAGAGGCGAGTC
CGTGGCAGCAGTTGTATCAACAGCCCCTAATCGCT

>pQR3140

MSTLHLLSLTDQIAGLAGGQFSSVELTQHYLDRIAALDGKVGSFITVTPEHALAQAQAADQARSAGRATALTG
LPFAHKDIFCTQGVRTSAGSKMLDNFISPYNATVVEKCAAAGLVMLGKTNMDEFAMGSSNESSYYGAAKNPWD
LGRVAGGSSGGSAAAIAADLTPLATGTDTGGSIRQPAAFCGLTGIKPTYGRVSRFGMIAYASSLDQGGPMARS
AADCAQLLQVMAGHDGKDSTSVDRPVDDYVTALGESIKGLRIGLPRQYFADGLDADVKARVMDALKVLEGLGA
QLVEIDLTTTDAAIPAYYLIAPAEASSNLSRFDGVRYGYRCDAPKDLYDLYTRSRAEGFGKEVQRRILIGTYA
LSAGYYDAYYLKAQKVRRLIQQDFLKAFEQCDVIAGPTVPSTAYALGAKQDPVAMYLGDIYTIAVNLAGLPAL
SAPCGFDAQGLPVGLQLIGNYWSEGRLLAAAHQYQQHTTWHTQRAPIAMEALEHHHHHH*

ATGTCAACACTGCATTTGCTCAGTTTAACAGACCAGATCGCAGGATTAGCGGGTGGTCAATTCTCCTCAGTTG
AACTGACTCAACACTATCTTGATCGGATTGCCGCACTCGATGGCAAGGTCGGTAGCTTTATTACCGTGACGCC
AGAGCATGCCCTCGCCCAAGCCCAAGCTGCGGATCAAGCCCGTTCGGCAGGCCGCGCTACGGCACTGACTGGT
TTGCCATTTGCCCACAAAGATATTTTTTGTACCCAAGGCGTGCGCACCTCGGCTGGCTCCAAGATGCTGGATA
ACTTTATCTCGCCATACAATGCCACGGTGGTAGAAAAATGCGCTGCGGCTGGCCTTGTGATGTTGGGTAAAAC
CAACATGGACGAGTTCGCGATGGGTTCATCCAACGAAAGCTCGTATTACGGCGCAGCCAAAAATCCGTGGGAT
TTGGGTCGGGTGGCGGGTGGCTCATCGGGTGGTTCAGCCGCTGCGATTGCCGCCGACCTCACCCCACTGGCAA
CGGGCACAGATACTGGGGGGTCGATTCGTCAGCCGGCGGCATTTTGTGGTTTGACTGGGATCAAGCCCACCTA
TGGTCGGGTGTCGCGCTTTGGCATGATTGCCTATGCATCCAGCCTTGATCAAGGCGGCCCGATGGCTCGCAGT
GCCGCAGACTGCGCACAACTGTTGCAAGTGATGGCAGGTCACGACGGCAAAGACTCCACCAGTGTGGATCGTC
CGGTGGATGACTATGTAACTGCACTCGGTGAGTCGATCAAGGGCTTGCGCATTGGCCTGCCGCGCCAGTATTT
TGCCGATGGCTTAGATGCCGATGTCAAAGCGCGGGTGATGGACGCGCTCAAAGTGCTTGAAGGCTTGGGCGCT
CAGTTGGTCGAGATTGATCTGACCACCACCGATGCCGCCATTCCAGCTTATTACTTGATTGCTCCTGCCGAAG
CCTCATCGAACTTGTCGCGTTTTGATGGGGTGCGCTATGGCTATCGCTGCGATGCACCCAAAGATTTGTATGA
CCTGTACACTCGCTCACGTGCCGAAGGTTTTGGCAAAGAAGTACAGCGCCGGATTTTGATTGGTACTTATGCG
CTCTCGGCGGGCTACTACGATGCCTATTATCTCAAAGCGCAAAAAGTCCGCCGCCTGATTCAACAAGACTTCC
TCAAAGCCTTTGAGCAATGCGATGTGATTGCAGGGCCGACCGTCCCAAGCACCGCTTATGCGCTTGGTGCCAA
GCAAGATCCGGTCGCGATGTATTTGGGCGATATCTACACCATCGCGGTCAACTTGGCCGGTCTGCCTGCCTTG
AGTGCCCCGTGTGGCTTCGATGCACAAGGTTTGCCGGTCGGTTTGCAATTGATTGGCAACTATTGGAGCGAAG
GCCGCTTGCTTGCTGCCGCGCACCAATACCAACAACACACCACATGGCACACCCAGCGTGCCCCAATCGCGAT
GGAGGCT

>pQR3141

MKFEEYRQHDALGLAELVARGETTAVDLLHCAQTRAAAVNPALNAIVIPMAEIAKKRVQETLSGPFAGVPFLI
KDIAQDYAGVPTTAGSRALRDYRPDRHSSYVQRALDSGLVIFGKTNTPEFALKGTTEPAFWGATRNPWDKTRT
PGGSSGGAAAAVAAGIVPMAGASDGGGSIRIPASHCGLFGLRPSRGRVPSGPYFAEFWHGASSEHVLTRSVRD
SAAMLDALQGPDTGAPFRIRPPERPYREEITHEPGRLRIAYSTRSPLGLGVDADCVRAVESSIALLRRLGHEV
EEAEPVVDGRALAEAFLTMYMGQTAANMAEAQRLTGAAESDFELDTRALASLGRALSAGEYTRSLFRWNDFAR
AVGEFFKHYDLYLTPTVATVPALIGEQDTPLWQQLALRPALPLGKLLLKSGQVQKNAYNNLWRVPFTQLSNLS
GTPSMSVPLHWTAAGLPVGVQFMAPSGEEARLLRLAAQLEQEQPWFARVPALEHHHHHH*

ATGAAATTCGAAGAGTACCGCCAGCACGATGCCCTGGGACTTGCCGAACTGGTGGCCCGTGGTGAAACCACCG
CTGTTGACCTGCTGCACTGCGCACAGACACGTGCCGCCGCGGTCAATCCCGCGCTGAACGCCATCGTCATTCC
CATGGCGGAGATCGCGAAAAAACGCGTGCAGGAAACCCTGTCCGGTCCTTTTGCCGGTGTGCCCTTCCTCATC
AAGGACATCGCCCAGGACTATGCCGGTGTGCCCACCACGGCCGGCAGTCGCGCCCTGCGCGATTACCGTCCTG
ATCGCCACTCTTCCTATGTGCAGCGTGCACTGGACAGCGGCCTGGTGATTTTCGGCAAAACCAATACGCCGGA
GTTCGCGCTCAAGGGCACCACGGAACCGGCCTTCTGGGGAGCGACACGCAATCCCTGGGATAAAACTCGTACA
CCGGGTGGCTCATCGGGCGGTGCTGCTGCCGCCGTTGCTGCCGGCATCGTGCCCATGGCCGGTGCCAGCGATG
GTGGTGGCTCCATACGTATTCCCGCCTCGCATTGCGGTCTCTTCGGTTTGCGCCCTTCACGCGGTCGCGTGCC
TTCGGGTCCTTACTTTGCCGAGTTCTGGCATGGCGCCTCCAGCGAACATGTGCTGACACGCAGTGTGCGTGAC
AGCGCCGCCATGCTGGATGCGCTGCAGGGACCGGACACGGGCGCGCCCTTCCGTATCCGGCCGCCCGAACGGC
CCTATCGTGAAGAAATCACGCACGAGCCGGGGCGGCTGCGTATTGCCTACTCCACGCGTTCACCGCTGGGACT
GGGAGTAGATGCCGATTGCGTAAGGGCCGTGGAAAGCAGCATCGCGCTGCTGCGCCGTCTGGGTCATGAAGTA
GAGGAAGCGGAGCCGGTGGTTGATGGTCGTGCCCTGGCCGAGGCTTTCCTCACCATGTATATGGGCCAGACCG
CGGCCAATATGGCCGAAGCCCAGCGCCTGACCGGCGCGGCGGAAAGTGATTTCGAACTGGATACCCGCGCCCT
GGCCTCTCTGGGCCGCGCGCTTTCTGCGGGCGAATACACCCGCTCGCTGTTCCGCTGGAATGATTTTGCGCGT
GCCGTGGGTGAATTCTTCAAGCACTACGATCTCTACCTGACACCGACCGTGGCCACGGTGCCGGCGCTGATAG
GCGAACAGGACACGCCCTTGTGGCAGCAGCTGGCCCTGCGTCCTGCCCTGCCCCTGGGCAAGCTGCTGCTGAA
AAGCGGGCAGGTGCAGAAGAATGCCTATAACAATCTCTGGCGTGTGCCTTTTACACAGCTGTCCAATCTGTCC
GGAACGCCATCGATGAGCGTGCCGCTGCACTGGACGGCCGCCGGCCTGCCGGTGGGCGTGCAATTCATGGCGC
CTTCCGGTGAGGAAGCGCGGCTGCTGCGGCTGGCGGCACAGCTGGAGCAGGAACAGCCGTGGTTTGCGCGTGT
GCCGGCT

>pQR3142

MTELTNLTVAQIRDGHRAGDFSAVEVAEAFNVNVAGAKLLNAFIVETPEHALAAAKAADADRAAGTLKPLSGV
PIGMKDLFCTDGVQTTAASHMLEGFVPRYESTVSQKLWDAGAGMLGKLNLDQFAMGSSNETSYFGNVISPWRR
KDGGNAALAPGGSSGGSSTAIAARLCPAATGTDTGGSIRQPAAFTGISGIKPTYGRCSRWGIVAFASSLDQAG
PMARDVRDCAIMLENMAGFDPKDATSLNLPVPDWEVALSSDLKGKTVGIPKEYRLEGIDPDIDAMWDAGIAML
KDAGADVVEISLPHTKYALPAYYIIAPAEASSNLARYDGVRYGLRDLPDGAGLQDMYAATRADGFGPEVKRRI
MIGTYVLSAGFYDAYYTQAQKVRTLIARDFEAAFGVCDVILAPTAPSAAFGLGEKMADPLAMYLNDVFAVPAS
LAGLPAMSVPAALNREGLPLGLQIIGKAFDEQGVLNAGLAIEERAGFNARAEKWWLEHHHHHH*

ATGACCGAGCTTACCAATCTGACCGTTGCCCAGATCCGCGACGGGCACCGCGCGGGCGATTTCAGCGCCGTTG
AGGTGGCGGAGGCGTTCAATGTCAATGTCGCGGGCGCGAAGCTGCTCAATGCGTTTATCGTCGAGACGCCCGA
GCATGCGCTTGCAGCGGCGAAAGCTGCCGATGCCGACCGCGCGGCGGGAACGCTGAAGCCGCTGTCGGGCGTG
CCGATCGGGATGAAGGATCTATTCTGCACCGATGGCGTCCAGACCACGGCGGCGAGCCACATGCTCGAAGGCT
TTGTCCCGCGCTATGAATCGACCGTCAGCCAGAAATTGTGGGACGCGGGCGCCGGGATGCTGGGCAAGCTGAA
CCTTGACCAGTTCGCGATGGGGTCGTCGAACGAGACTTCCTACTTTGGCAATGTCATCAGCCCGTGGCGGCGC
AAGGACGGCGGCAACGCTGCACTCGCGCCGGGCGGCTCCTCGGGCGGCTCGTCGACCGCGATCGCGGCGCGAC
TGTGCCCTGCGGCGACGGGGACCGACACCGGCGGCTCGATCCGCCAGCCCGCGGCCTTCACCGGCATTTCGGG
GATCAAGCCGACCTATGGCCGCTGCTCGCGCTGGGGCATTGTGGCGTTCGCCAGCTCGCTCGATCAGGCGGGA
CCGATGGCGCGCGACGTGCGTGACTGCGCTATCATGCTCGAAAATATGGCAGGCTTCGATCCGAAGGATGCGA
CGAGCCTGAACCTGCCCGTGCCCGATTGGGAAGTGGCTTTGTCTAGCGATCTTAAGGGCAAGACGGTCGGTAT
TCCCAAGGAATATCGGCTGGAGGGCATCGATCCCGACATCGACGCGATGTGGGATGCGGGCATCGCGATGCTG

AAGGATGCGGGCGCGGACGTGGTCGAGATCAGCCTGCCGCACACCAAATATGCGCTGCCCGCCTATTACATCA
TCGCGCCCGCCGAGGCGTCGTCGAACCTCGCGCGCTATGACGGCGTGCGTTACGGTTTGCGCGACCTGCCGGA
CGGGGCGGGGTTGCAGGACATGTACGCCGCGACGCGCGCCGACGGCTTCGGGCCCGAGGTCAAGCGCCGCATC
ATGATCGGCACCTATGTGCTCTCGGCCGGCTTTTACGACGCTTATTATACGCAGGCGCAGAAGGTGCGGACGC
TGATCGCGCGCGATTTCGAGGCGGCGTTCGGGGTGTGCGACGTGATCCTCGCGCCGACGGCGCCGTCGGCGGC
GTTCGGGCTCGGCGAGAAGATGGCCGATCCGCTGGCGATGTACCTCAATGACGTGTTCGCGGTTCCCGCGAGC
CTCGCGGGGCTGCCCGCGATGTCGGTTCCCGCAGCGCTGAACCGCGAAGGGCTGCCGCTGGGGCTTCAGATCA
TCGGCAAGGCGTTCGACGAGCAGGGCGTGCTGAACGCCGGGCTGGCAATTGAAGAGCGGGCAGGCTTCAACGC
GCGTGCGGAGAAGTGGTGG

>pQR3143

MSGNEELIQMTARQAVAALKKKEVKPVELVEAAIRRIEKVDGEINALPVKMFDDARLAAKAFDEKPDGKGPGW
LAGLPFVAKEMNDVKGYPTTYGSPVYKDNIAKDTDITVRVMQQSGGIVMAKSNVPEFAGSNTFNSVYGKTRNP
WDLRMSAGGSSGGAAAALASGTAWLAMGSDVGGSLRIPASYCGVVGLRPTPGRVARGRKLLPFDPIWVEGPMA
RNVDDLALMLDAQSTYVEEDPLSRPPPSIPFSEQIKVTQKGLKIAYSSNFGLTEVDKEVAEVTLAAAKRLEEI
GAFVSENCPDLSGSIDAFQTQRAVLFASVHGEIVKKERDRIPASIVWNIEKGLNIKADEVLRAERERQRLVQV
MRVFFESVDFFVCPTVSVPPFIVEQAYPTEINGRKMETYIDWMAMTFVITVTGNPAISVPCGFTASGLPIGLQ
IVGKHNTEGQLLALAAQLEAVLKLPRTPIDPRQGLEHHHHHH*

ATGTCAGGGAACGAAGAACTGATACAGATGACCGCACGGCAAGCGGTCGCCGCGCTTAAGAAGAAAGAAGTGA
AGCCTGTCGAGCTTGTCGAAGCCGCCATAAGGCGTATCGAGAAGGTTGACGGTGAAATCAACGCGCTGCCGGT
CAAGATGTTCGATGACGCTCGACTGGCGGCAAAGGCGTTCGATGAGAAGCCCGATGGCAAAGGGCCGGGTTGG
CTGGCAGGTCTGCCGTTCGTCGCCAAGGAAATGAACGACGTCAAAGGCTATCCGACAACTTATGGATCGCCAG
TCTACAAGGACAATATCGCCAAGGACACCGACATCACAGTTCGCGTCATGCAGCAGTCTGGCGGCATCGTGAT
GGCGAAGTCAAACGTGCCAGAGTTTGCGGGCTCCAACACTTTCAACTCTGTGTATGGAAAGACCCGTAACCCC
TGGGATTTGCGCATGTCGGCTGGTGGGTCTTCCGGTGGTGCTGCAGCGGCACTGGCGAGCGGCACTGCATGGC
TGGCCATGGGCAGCGACGTCGGCGGCAGTCTCCGCATTCCGGCCAGCTACTGTGGCGTCGTGGGGCTCCGGCC
TACGCCTGGAAGAGTTGCCCGTGGACGTAAGCTTCTCCCCTTCGACCCAATCTGGGTGGAGGGACCTATGGCT
CGAAATGTCGACGACCTCGCGCTCATGCTCGACGCTCAGTCCACCTATGTCGAGGAAGACCCGCTTTCACGAC
CACCTCCGAGCATCCCGTTCTCCGAGCAGATCAAGGTTACTCAGAAAGGCCTTAAAATCGCCTATTCCAGCAA
CTTTGGTTTGACCGAAGTTGATAAGGAAGTGGCGGAAGTGACGCTGGCGGCGGCGAAGCGCCTGGAGGAAATC
GGGGCGTTCGTAAGCGAGAACTGCCCTGATCTGAGTGGCTCCATTGATGCATTCCAGACCCAGCGAGCCGTTC
TCTTCGCGTCTGTGCATGGCGAGATCGTCAAAAAGGAACGCGACCGAATACCGGCGAGCATCGTCTGGAACAT
CGAAAAGGGCCTGAATATCAAGGCTGACGAGGTCCTGCGTGCCGAGCGCGAGCGGCAGCGTCTGGTTCAAGTG
ATGCGCGTTTTCTTTGAAAGCGTGGACTTCTTCGTCTGTCCGACAGTGTCGGTGCCGCCATTTATTGTCGAGC
AGGCATATCCGACGGAAATCAACGGCCGGAAGATGGAAACCTACATCGATTGGATGGCTATGACCTTCGTCAT
CACTGTGACAGGGAATCCGGCAATTTCGGTTCCCTGCGGTTTCACCGCAAGTGGTTTGCCAATCGGTCTCCAG
ATTGTCGGAAAGCATAACACGGAAGGCCAATTGCTTGCGCTCGCAGCGCAGCTAGAGGCGGTCCTGAAACTGC
CTCGGACGCCGATTGACCCGCGGCAGGGT

>pQR3144

MPPSKTVHAFSNDILANHDAVALADLIRRREISPAEVTQAAIARAEQVNPALNAIRLETFEAARQQARQPLQG
VFAGVPTFIKDNTDLRGLPTSQGSQAVHPRPAKADGAFARQFLQQGVICLGKSQLPEFGLNASTEFLGQPPTR
NPWHTDYSSGASSGGSAALVAAGVVPLAHANDGGGSIRIPAAACGLVGLKPTRGRLVDGEAARTLPVNIVSEG
VVTRSVRDTAHFYAGAEKYFRNAKLPAIGLVEGPGRKRLRIGVVYDSITGHPSDDDTRRTVGDTARLLAGSGH
HVTEMALPITPRFIDDFSTYWGMLSFLVSRFGHRILSPDFNADELDNLTKGLAALYRRNALKTPLVLYRLRKT
WDDYARVFRDYDIVLSPVLAHSTPELGWLSPEQPFEELFERLLRYVSFTPLNNAAGSPAISLPMGATAQGLPV
AVHCSAAHGAERTLLELAFELEQAKPWRRIQDQLEHHHHHH*

ATGCCGCCCTCCAAAACCGTCCATGCCTTCAGCAACGATATCCTGGCCAACCACGATGCCGTCGCCCTGGCCG
ACCTGATACGCCGCCGTGAAATCAGTCCCGCCGAAGTCACGCAGGCCGCCATTGCCCGCGCTGAACAGGTGAA
CCCGGCCCTGAACGCCATACGCCTGGAAACTTTCGAGGCCGCCCGGCAGCAGGCGCGCCAACCGCTGCAGGGC

GTGTTTGCCGGCGTACCCACTTTCATCAAGGACAACACCGACCTGCGCGGCCTGCCCACCAGCCAGGGCTCAC
AGGCGGTGCATCCGCGTCCGGCCAAGGCCGACGGCGCTTTTGCGCGGCAATTCCTGCAGCAGGGCGTGATCTG
CCTGGGCAAAAGCCAGCTGCCGGAATTCGGCCTCAATGCCAGCACGGAATTCCTGGGCCAGCCGCCGACACGC
AATCCCTGGCATACCGATTATTCCAGCGGCGCCTCCTCCGGCGGTTCCGCCGCACTGGTCGCGGCCGGTGTGG
TGCCGCTGGCACATGCCAATGACGGTGGCGGCTCCATACGCATTCCCGCCGCGGCCTGCGGTCTTGTCGGCCT
CAAGCCCACGCGCGGCCGTCTCGTCGATGGCGAAGCGGCGCGTACCTTGCCGGTGAATATCGTGTCGGAAGGC
GTGGTCACGCGCAGCGTGCGCGACACCGCGCATTTCTATGCCGGCGCGGAAAAATATTTCCGTAATGCCAAGC
TGCCTGCCATCGGCCTGGTGGAAGGCCCTGGGCGTAAACGCCTGCGCATAGGCGTGGTCTACGACTCCATCAC
CGGCCATCCCAGTGATGACGACACACGTCGCACCGTGGGCGACACCGCACGCCTGCTGGCAGGATCAGGCCAC
CATGTCACCGAGATGGCGCTGCCCATCACACCGCGCTTTATCGATGATTTCAGTACTTACTGGGGCATGCTGT
CCTTTCTGGTCAGCCGTTTCGGCCACCGCATTCTCAGCCCTGACTTCAATGCCGACGAACTCGACAACCTCAC
CAAAGGCCTGGCCGCGCTTTACCGCCGCAATGCGCTGAAGACGCCCCTGGTGCTCTACCGCCTGCGCAAGACC
TGGGATGACTACGCCCGTGTCTTCCGCGACTACGACATCGTGCTGTCGCCCGTGCTGGCACACAGCACGCCTG
AGCTGGGCTGGCTGTCGCCGGAGCAGCCTTTCGAGGAATTGTTCGAGCGTCTGCTGCGCTATGTGAGTTTCAC
ACCACTGAACAATGCCGCGGGCAGCCCGGCGATTTCCCTGCCCATGGGCGCCACCGCCCAAGGCCTGCCGGTA
GCGGTGCATTGCTCTGCCGCCCATGGTGCCGAACGCACGCTGCTGGAACTGGCTTTTGAACTGGAACAGGCCA
AACCCTGGCGCCGTATCCAGGACCA

>pQR3145

MGFSAGGSSSGSGVVVALGEVDMAIGGDQGGSIRMPSSFCGTYGMKPTWGLVPYTGIMPIEIFVDHTGPMTAS
VADNALLLEVLAGDDGYDPRIKAPKVEEYTKALGGGVKGLKIGILKEGFEQPGAETAVNESVREAAKRLRSLG
ATVEDVSVPMHMVGPAIWTPIGTEGMTQTMMYGDGYGLSRSDLYSTSLMDFHRGWRRQADSLSETTKLFLLLG
TYINNNFGPRYYGKALNVSRRLTAAYDAVLKDYDLLLLPTTPMKATPLPPANASREDYVARALEMITNTAPFD
ITHHPAMSLPCGMVDGLPVGLMLVGRHFDESTIYRAAHAFEQAGDWKKMLEHHHHHH*

ATGGGCTTTTCGGCCGGCGGGTCGTCGTCGGGCAGCGGCGTCGTCGTCGCGCTCGGAGAGGTCGACATGGCGA
TCGGCGGCGACCAGGGCGGCTCGATCCGCATGCCGTCCTCGTTCTGCGGCACCTACGGCATGAAGCCGACCTG
GGGTCTTGTCCCCTACACCGGCATCATGCCGATCGAAATCTTCGTCGATCACACCGGTCCGATGACGGCCAGC
GTCGCCGACAATGCCCTGCTGCTCGAAGTGCTCGCCGGCGACGACGGCTACGATCCGCGGATCAAGGCGCCGA
AGGTCGAGGAGTACACCAAGGCGCTCGGCGGCGGCGTCAAGGGTCTCAAGATCGGCATCCTCAAGGAAGGGTT
CGAGCAGCCAGGGGCCGAGACGGCGGTGAACGAAAGCGTGCGCGAGGCGGCAAAGCGCCTTCGCAGCCTGGGC
GCGACCGTCGAGGACGTCTCCGTCCCGATGCATATGGTGGGGCCGGCGATCTGGACGCCGATCGGGACCGAAG
GCATGACGCAGACCATGATGTATGGTGACGGCTACGGCCTGAGCCGGTCGGACCTGTATTCGACATCGCTGAT
GGATTTCCATCGCGGCTGGCGCCGGCAGGCCGACTCGCTCTCCGAGACCACGAAGCTGTTCCTGCTGCTCGGC
ACCTACATCAACAACAATTTCGGGCCGCGCTACTACGGCAAGGCGCTCAACGTTTCCCGCCGGTTGACCGCGG
CGTATGACGCGGTTCTGAAGGACTACGATCTGCTGCTGCTGCCGACGACGCCGATGAAGGCGACGCCGCTGCC
GCCGGCGAACGCCAGCCGCGAGGACTATGTCGCACGCGCCCTCGAAATGATCACCAACACGGCGCCCTTCGAC
ATCACCCACCATCCGGCGATGTCGTTGCCATGCGGCATGGTCGATGGCCTTCCGGTCGGATTGATGCTGGTCG
GCCGCCACTTCGATGAATCGACGATCTATCGCGCTGCGCATGCCTTCGAGCAGGCCGGCGACTGGAAGAAGAT

1.3 Protein and DNA sequences of UMG-SP amidases

>UMG-SP-1

MAREPTALATAAAIRSGATTARAETEAAIARIEALDGAINAVVVRDFDRALAAADAADARIQAGDTAPLLGVP
MTVKEAFDVEGLPTHWGFRQHAGNIATSDAEAVRRLKAAGAIILGKTNVPKGLGDWQSVNSIHGVTNHPLDPT
RTPGGSSGGSAAALASGMVPLELGSDIGGSIRIPAHFCGVWGLKPSWGAISSHGHRYPGTNGAETPLGVIGPM
ARSPDDLAAMLDLLATLPMPRASRPPRRVLAITDHPAIRTSAVCRDAVDTAAEALAGAGIEVIRSTDLLPDLA
RQHHAYGQMLSVAFARSDPTLHASLPNLLTWLSWQDAQARNTRAWGRLFGEVDAVIAPPAATQAFAHDHAPQA
NRTLDIDGVASPYDAHLAWAGVATYPGLPAVVVPVGTANGLPVGVQVITDFHRDHDAIATAALIHRLTEGQPA
LEHHHHHH*

ATGGCGCGCGAACCGACCGCGCTGGCGACCGCGGCGGCGATTCGCAGCGGCGCGACCACCGCGCGCGCGGAAA
CCGAAGCGGCGATTGCGCGCATTGAAGCGCTGGATGGCGCGATTAACGCGGTGGTGGTGCGCGATTTTGATCG
CGCGCTGGCGGCGGCGGATGCGGCGGATGCGCGCATTCAGGCGGGCGATACCGCGCCGCTGCTGGGCGTGCCG
ATGACCGTGAAAGAAGCGTTTGATGTGGAAGGCCTGCCGACCCATTGGGGCTTTCGCCAGCATGCGGGCAACA
TTGCGACCAGCGATGCGGAAGCGGTGCGCCGCCTGAAAGCGGCGGGCGCGATTATTCTGGGCAAAACCAACGT
GCCGAAAGGCCTGGGCGATTGGCAGAGCGTGAACAGCATTCATGGCGTGACCAACCATCCGCTGGATCCGACC
CGCACCCCGGGCGGCAGCAGCGGCGGCAGCGCGGCGGCGCTGGCGAGCGGCATGGTGCCGCTGGAACTGGGCA
GCGATATTGGCGGCAGCATTCGCATTCCGGCGCACTTTTGCGGCGTGTGGGGCCTGAAACCGAGCTGGGGCGC
GATTAGCAGCCATGGCCATCGCTATCCGGGCACCAACGGCGCGGAAACCCCGCTGGGCGTGATTGGCCCGATG
GCGCGCAGCCCGGATGATCTGGCGGCGATGCTGGATCTGCTGGCGACCCTGCCGATGCCGCGCGCGAGCCGCC
CGCCGCGCCGCGTGCTGGCGATTACCGATCATCCGGCGATTCGCACCAGCGCGGTGTGCCGCGATGCGGTGGA
TACCGCGGCGGAAGCGCTGGCGGGCGCGGGCATTGAAGTGATTCGCAGCACCGATCTGCTGCCGGATCTGGCG
CGCCAGCATCATGCGTATGGCCAGATGCTGAGCGTGGCGTTTGCGCGCAGCGATCCGACCCTGCATGCGAGCC
TGCCGAACCTGCTGACCTGGCTGAGCTGGCAGGATGCGCAGGCGCGCAACACCCGCGCGTGGGGCCGCCTGTT
TGGCGAAGTGGATGCGGTGATTGCGCCGCCGGCGGCGACCCAGGCGTTTGCGCATGATCATGCGCCGCAGGCG
AACCGCACCCTGGATATTGATGGCGTGGCGAGCCCGTATGATGCGCATCTGGCGTGGGCGGGCGTGGCGACCT
ATCCGGGCCTGCCGGCGGTGGTGGTGCCGGTGGGCACCGCGAACGGCCTGCCGGTGGGCGTGCAGGTGATTAC
CGATTTTCATCGCGATCATGATGCGATTGCGACCGCGGCGCTGATTCATCGCCTGACCGAAGGCCAGCCGGCG
CTCGAGCACCACCACCACCACCACTGA

>UMG-SP-2
MSELSAIETAAAIAGGSMTALEACDAAIARIEQRDGPINAVVVRDFDRARDAAKAADAEIAAAVRKPLLGVPM
TIKESFDIAGLPTSWGFAEHADHIATADSLVVSRLKAAGAVFLGKSNIPVGLADWQSVNPNYGRTNNPHDHSR
SAGGSSGGAAAALAAGMVPLEYGSDIGGSIRVPAHFCGVWGLKTTFDAVSLEGHYFPRTDSAKADLSVVGPMA
RTPADLALALDITSKVPLPQSRIANLSGLRILLLTAHPETVADSATISAVERAAAACEASGATVATSSPDLPD
LSALVADYTRMLLVVLARGLAPEGTEPVSLNAWYAMLDDQARMMRAFDRLFESFDAIFCPVLGTTAFAHSDEP
DWAKRSLSIDGGIAPFAAQLGWISMATYGGMPALSMPLGADGNGLPINLQIITRNWSDHDAIRIGALVAEALD
RLEHHHHHH*

ATGAGCGAACTGAGCGCGATTGAAACCGCGGCGGCGATTGCGGGCGGCAGCATGACCGCGCTGGAAGCGTGCG
ATGCGGCGATTGCGCGCATTGAACAGCGCGATGGCCCGATTAACGCGGTGGTGGTGCGCGATTTTGATCGCGC
GCGCGATGCGGCGAAAGCGGCGGATGCGGAAATTGCGGCGGCGGTGCGCAAACCGCTGCTGGGCGTGCCGATG
ACCATTAAAGAAAGCTTTGATATTGCGGGCCTGCCGACCAGCTGGGGCTTTGCGGAACATGCGGATCATATTG
CGACCGCGGATAGCCTGGTGGTGAGCCGCCTGAAAGCGGCGGGCGCGGTGTTTCTGGGCAAAAGCAACATTCC
GGTGGGCCTGGCGGATTGGCAGAGCGTGAACCCGAACTATGGCCGCACCAACAACCCGCATGATCATAGCCGC
AGCGCGGGCGGCAGCAGCGGCGGCGCGGCGGCGGCGCTGGCGGCGGGCATGGTGCCGCTGGAATATGGCAGCG
ATATTGGCGGCAGCATTCGCGTGCCGGCGCACTTTTGCGGCGTGTGGGGCCTGAAAACCACCTTTGATGCGGT
GAGCCTGGAAGGCCATTATTTTCCGCGCACCGATAGCGCGAAAGCGGATCTGAGCGTGGTGGGCCCGATGGCG
CGCACCCCGGCGGATCTGGCGCTGGCGCTGGATATTACCAGCAAAGTGCCGCTGCCGCAGAGCCGCATTGCGA
ACCTGAGCGGCCTGCGCATTCTGCTGCTGACCGCGCATCCGGAAACCGTGGCGGATAGCGCGACCATTAGCGC
GGTGGAACGCGCGGCGGCGGCGTGCGAAGCGAGCGGCGCGACCGTGGCGACCAGCAGCCCGGATCTGCCGGAT
CTGAGCGCGCTGGTGGCGGATTATACCCGCATGCTGCTGGTGGTGCTGGCGCGCGGCCTGGCGCCGGAAGGCA
CCGAACCGGTGAGCCTGAACGCGTGGTATGCGATGCTGGATGATCAGGCGCGCATGATGCGCGCGTTTGATCG
CCTGTTTGAAAGCTTTGATGCGATTTTCTGCCCGGTGCTGGGCACCACCGCGTTTGCGCATAGCGATGAACCG
GATTGGGCGAAACGCAGCCTGAGCATTGATGGCGGCATTGCGCCGTTTGCGGCGCAGCTGGGCTGGATTAGCA
TGGCGACCTATGGCGGCATGCCGGCGCTGAGCATGCCGCTGGGCGCGGATGGCAACGGCCTGCCGATTAACCT
GCAGATTATTACCCGCAACTGGAGCGATCATGATGCGATTCGCATTGGCGCGCTGGTGGCGGAAGCGCTGGAT
CGCCTCGAGCACCACCACCACCACCACTGA

>UMG-SP-3

MSELSAIETAAAIAGGSMTALEACDAAIARIEQRDGPINAVVVRDFDRAREAAKAADGEVAAGVSKPLLGVPM
TIKESIDIAGLPTSWGFAEHADHIATADSVVVSRLKAAGAVFLGKTNIPVALADWQSSNPNYGRTNNPHDLTR
SAGGSSGGAAAALAAGMVPLEYGSDIGGSIRVPAHFCGVWGLKTTFDAVSLEGHYLPRTDGARGELGVVGPMA
RNPQDLALALDLTSRIALPIARIDTLNGLRILLLTHHPRAAADSAVVAAVEKAAESCAAQGAQVSTSNADLPD
LSKLVSDYTRMLLIVLAQGKAPEGTEPVSLNAWYGMLDDQARTIRGFDRLFDSFDAIFCPVLGTSAFPHSDEA
DWGKRTLTIDGADTPFGSQLAWISMATYCGMPALSMPVGTDANGLPIGLQIITRNWSDHDAVRIGALVADALA
ALEHHHHHH*

ATGAGCGAACTGAGCGCGATTGAAACCGCGGCGGCGATTGCGGGCGGCAGCATGACCGCGCTGGAAGCGTGCG
ATGCGGCGATTGCGCGCATTGAACAGCGCGATGGCCCGATTAACGCGGTGGTGGTGCGCGATTTTGATCGCGC
GCGCGAAGCGGCGAAAGCGGCGGATGGCGAAGTGGCGGCGGGCGTGAGCAAACCGCTGCTGGGCGTGCCGATG
ACCATTAAAGAAAGCATTGATATTGCGGGCCTGCCGACCAGCTGGGGCTTTGCGGAACATGCGGATCATATTG
CGACCGCGGATAGCGTGGTGGTGAGCCGCCTGAAAGCGGCGGGCGCGGTGTTTCTGGGCAAAACCAACATTCC
GGTGGCGCTGGCGGATTGGCAGAGCAGCAACCCGAACTATGGCCGCACCAACAACCCGCATGATCTGACCCGC
AGCGCGGGCGGCAGCAGCGGCGGCGCGGCGGCGGCGCTGGCGGCGGGCATGGTGCCGCTGGAATATGGCAGCG
ATATTGGCGGCAGCATTCGCGTGCCGGCGCACTTTTGCGGCGTGTGGGGCCTGAAAACCACCTTTGATGCGGT
GAGCCTGGAAGGCCATTATCTGCCGCGCACCGATGGCGCGCGCGGCGAACTGGGCGTGGTGGGCCCGATGGCG
CGCAACCCGCAGGATCTGGCGCTGGCGCTGGATCTGACCAGCCGCATTGCGCTGCCGATTGCGCGCATTGATA
CCCTGAACGGCCTGCGCATTCTGCTGCTGACCCATCATCCGCGCGCGGCGGCGGATAGCGCGGTGGTGGCGGC
GGTGGAAAAGGCGGCGGAAAGCTGCGCGGCGCAGGGCGCGCAGGTGAGCACCAGCAACGCGGATCTGCCGGAT
CTGAGCAAACTGGTGAGCGATTATACCCGCATGCTGCTGATTGTGCTGGCGCAGGGCAAAGCGCCGGAAGGCA
CCGAACCGGTGAGCCTGAACGCGTGGTATGGCATGCTGGATGATCAGGCGCGCACCATTCGCGGCTTTGATCG
CCTGTTTGATAGCTTTGATGCGATTTTCTGCCCGGTGCTGGGCACCAGCGCGTTTCCGCATAGCGATGAAGCG
GATTGGGGCAAACGCACCCTGACCATTGATGGCGCGGATACCCCGTTTGGCAGCCAGCTGGCGTGGATTAGCA
TGGCGACCTATTGCGGCATGCCGGCGCTGAGCATGCCGGTGGGCACCGATGCGAACGGCCTGCCGATTGGCCT
GCAGATTATTACCCGCAACTGGAGCGATCATGATGCGGTGCGCATTGGCGCGCTGGTGGCGGATGCGCTGGCG
GCGCTCGAGCACCACCACCACCACCACTGA

1.4 Multiple sequence alignment (MSA) of putative amidases pQR3137-3145 and urethanases from the literature

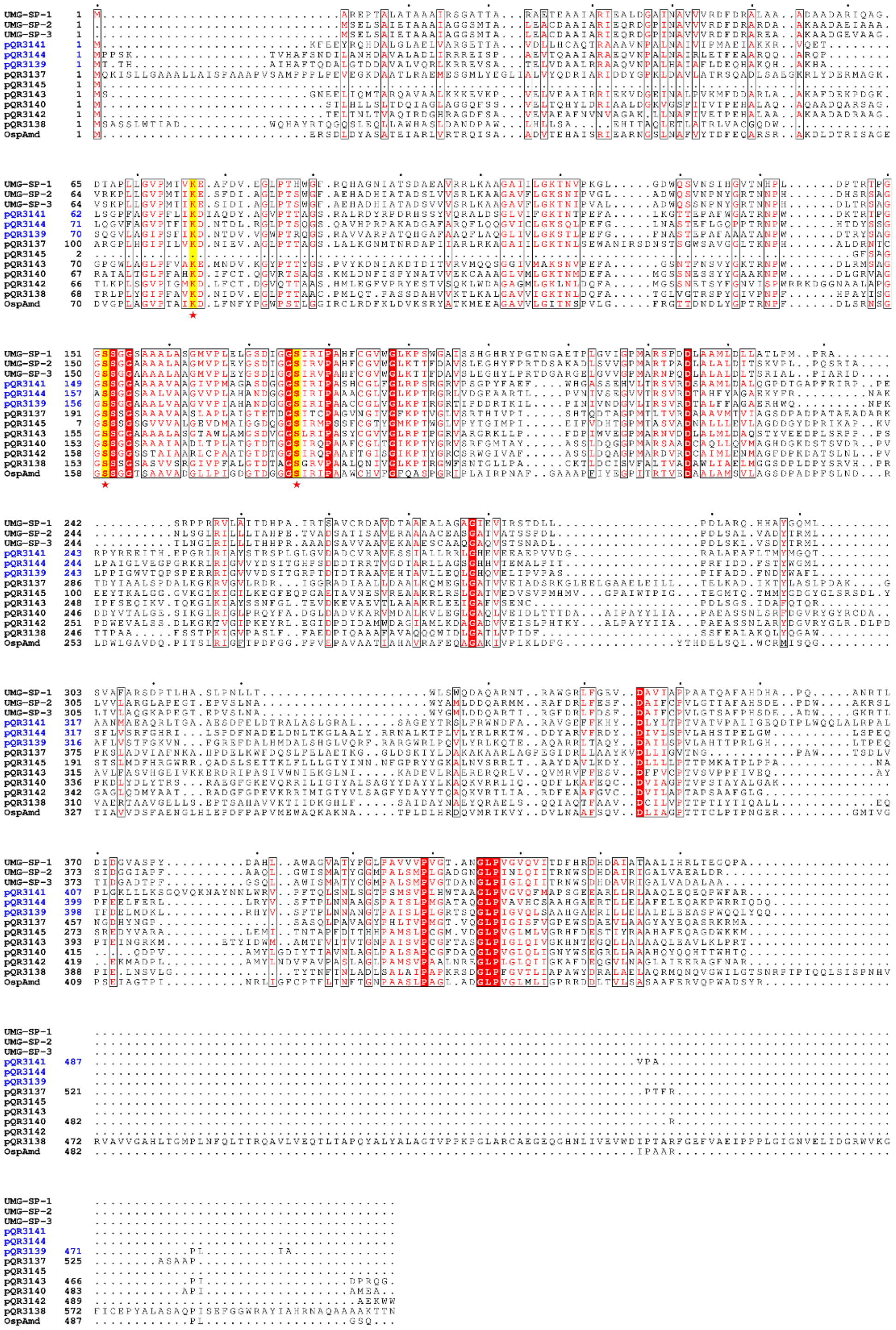

Figure S1 *MSA of metagenomic putative amidases. Key catalytic triad residues are highlighted in yellow and marked by a red star. Active enzyme's IDs from this study are highlighted in blue. MSA formatted with ESPript 3.*[1]

1.5 Percentage of identity matrix and phylogenetic analysis

The percentage of identity matrix was obtained by aligning the protein sequences with clustal Omega (1.2.4).[2] The matrix was then visualised and formatted in Microsoft Excel.

Starting from a protein alignment created with MAFFT version 7,[3] accessed via the MPI Bioinformatics Toolkit,[4,5] a phylogenetic analysis was performed with IQ-TREE version 2.3.6[6,7] using the following parameters: -m MFP -b 1000 (1000 bootstrap replicates) -alrt 1000 -bnni. The best model according to the Bayesian Information Criterion (BIC) was Q.pfam+F+I+G4. The maximum likelihood tree was then visualised and formatted using TreeViewer version 2.2.0.[8]

1.6 BLAST search results with percentage identity to known proteins

***Table S1**. List of metagenomic enzymes and the respective BLAST search results with percentage identity and accession codes.*

Enzyme	Protein origin in NCBI database	Percentage identity / %	Accession code
pQR3137	unclassified Sphingopyxis	84.9	WP_056371756
pQR3138	Acinetobacter johnsonii	62.1	WP_411935676.1
pQR3139	Alcanivoracaceae bacterium	61.9	MFN3714646.1
pQR3140	Pseudomonadota bacterium	82.6	MEY4516224.1
pQR3141	Moraxellaceae bacterium	95.3	HEX6592331.1
pQR3142	Sphingopyxis sp.	97.4	MFN7029724.1
pQR3143	Bradyrhizobium sp. KB893862 SZCCT0404	74.3	WP_212028535.1
pQR3144	Moraxellaceae bacterium	94.2	HEX6591620.1
pQR3145	unclassified Bradyrhizobium	93.2	WP_334366095.1

1.7 Metagenomic amidases expression and purification

The genes for enzymes pQR3137-3145 were amplified from a drain metagenome by standard PCR methods and cloned into pET-29a(+). PCR reactions were carried out in 50 µL volumes and contained the following: 50 ng template DNA, 0.5 µM forward primer, 0.5 µM reverse primer, 0.2 mM dNTPs, 0.50 µL Q5 Hot Start High-Fidelity DNA Polymerase (NEB), 1X Q5 Reaction Buffer (NEB) and 1X Q5 High GC Enhancer (NEB). A list of primers can be found below, with NdeI and XhoI restriction sites marked in bold.

Gene	Forward primer	Reverse primer
pQR3137	AGGA**CATATG**CAAAAAATTTCGCTGCTCG	ATAA**CTCGAG**GGGCGCGGCAC
pQR3138	ATAG**CATATG**TCCGCCTCCTCTTTG	ATAA**CTCGAG**ATTGGTTGTTTTTGCGGCAG
pQR3139	AGAA**CATATG**ACGACGCACGCC	ATAA**CTCGAG**AGCGATTAGGGGCTGTTG
pQR3140	AGAA**CATATG**TCAACACTGCATTTGCTCAG	ATAA**CTCGAG**AGCCTCCATCGCGATTG
pQR3141	AGGA**CATATG**AAATTCGAAGAGTACCGCC	ATAA**CTCGAG**AGCCGGCACACGC
pQR3142	AGGA**CATATG**ACCGAGCTTACCAATCTGA	ATAA**CTCGAG**CCACCACTTCTCCGCAC
pQR3143	AGAG**CATATG**TCAGGGAACGAAGAACTG	ATAA**CTCGAG**ACCCTGCCGCGG
pQR3144	CGAT**CATATG**CCGCCCTCCAAAAC	ATAA**CTCGAG**CTGGTCCTGGATACGGC
pQR3145	CGAG**ATTAAT**GGGCTTTTCGGCC	ATAA**CTCGAG**CATCTTCTTCCAGTCGCC

Alternatively, the codon optimised genes for expression in *E. coli* were obtained in pET-29a(+) from GenScript. BL21(DE3) competent *E. coli* cells were transformed with each plasmid following the manufacturer's instructions. Single colonies were used to inoculate 10 mL of LB media supplemented with 50 µg/mL kanamycin, which was incubated at 37 °C overnight at 250 rpm. 2 mL of inoculum of the overnight cultures

was then used to inoculate 200 mL of LB media with 50 µg/mL kanamycin. The cultures were incubated at 37 °C and shaken at 250 rpm until the OD_{600} reached 0.4, then induced with 0.5 mM IPTG and the temperature was lowered to 25 °C for overnight protein expression. The cells were then harvested by centrifugation (5000 rcf, 30 min, 4 °C) and the pellet was either frozen at -80 °C for storage or resuspended in a buffer depending on the chosen level of purity of the enzymes.

Clarified cell lysate. The pellet was resuspended in 50 mM KPi buffer at pH 7.5, lysed by sonication (15 sec on 20 sec off, 15 cycles, 75% intensity), and clarified by centrifugation (10000 rcf, 40 min, 4 °C). The supernatant was divided in 200-500 µL aliquots, flash frozen and stored at -20 °C. Total protein concentrations were calculated using standard Bradford assays and the target enzyme percentage concentration was calculated by gel densitometry using ImageJ software.

Purified enzymes. The pellet was resuspended in Buffer A (50 mM KPi buffer pH 7.5, 20 mM imidazole, 500 mM NaCl, 4 °C), lysed by sonication (15 sec on 20 sec off, 15 cycles, 75% intensity), and clarified by centrifugation (10000 rcf, 40 min, 4 °C). The filtered supernatant was loaded onto a nickel affinity column containing 4-8 mL of Ni-resin which had previously been washed with water (4 column volume (CV)) and then equilibrated with Buffer A (4 CV) at 4 °C. Stepwise protein elution was achieved using wash Buffer B (50 mM KPi buffer pH 7.5, 40 mM imidazole, 500 mM NaCl, 4 °C, 4 CV) and Buffer C (50 mM KPi buffer pH 7.5, 100 mM imidazole, 500 mM NaCl, 4 °C, 4 CV) followed by elution Buffer D (50 mM KPi buffer pH 7.5, 500 mM imidazole, 500 mM NaCl, 4 °C, 1-4 CV). The eluted protein of interest was concentrated to approximately 2.5 mL and was buffer exchanged into 50 mM KPi buffer at pH 7.5 using PD-10 Desalting Columns (Cytiva). The final purity and concentration were assessed by SDS-PAGE gel and Bradford assay respectively. The purified proteins were aliquoted in 100-200 µL samples, flash frozen, stored at -20 °C and used within 1 month.

1.8 SDS-PAGE gel of pQR3137-3145

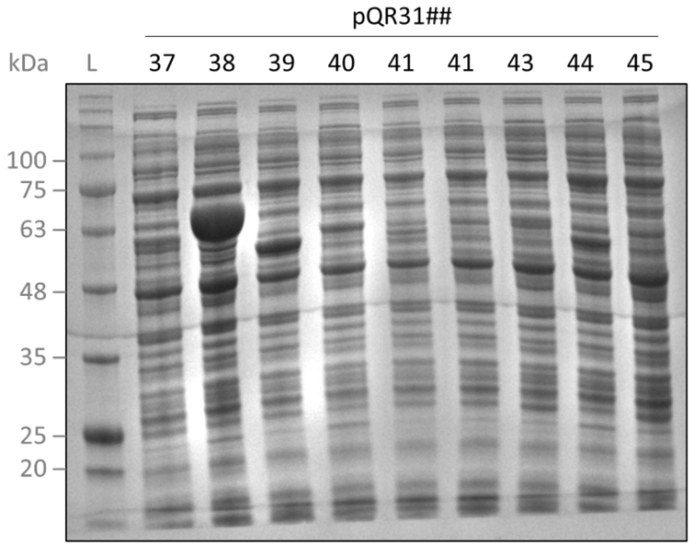

Gene pQR	MW / kDa
3137	56.0
3138	66.0
3139	52.3
3140	52.9
3141	53.9
3142	53.0
3143	52.2
3144	52.1
3145	37.7

***Figure S2**. SDS-PAGE gel showing the expression of metagenomic amidases pQR3137-3145 in CCL and their relative MW. L = ladder.*

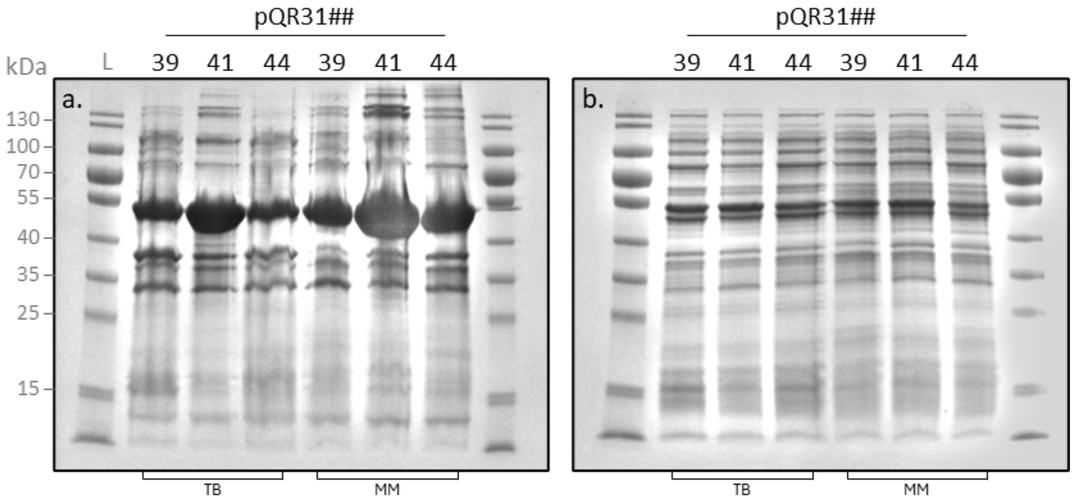

Figure S3. *SDS-PAGE gels showing the expression of pQR3139, 3141 and 3144 in CCL in TB or MagicMedia®. Insoluble pellet (a.) and soluble fraction (b.) shown.*

1.9 List of tyrosinases and growth conditions

Table S2. *Tyrosinases with species and databases accession codes.*

Enzyme	Bacterium	UniProtKB/GeneBank
*Rs*TYR[9]	*Ralstonia solanacearum*	Q8Y2J8/CAD13865
*Cn*TYR[9,10]	*Candidatus* Nitrosopumilus salaria	EIJ65432
*Cn*TYR_N201S[10]	*Candidatus* Nitrosopumilus salaria	-

BL21(DE3) glycerol stocks of the TYR genes in pJ401 were used to inoculate 10 mL of LB media supplemented with 50 µg/mL kanamycin, which was incubated at 37 °C overnight at 250 rpm. The overnight cultures were then used to inoculate 100 mL of TB media with 50 µg/mL kanamycin at a 1% final concentration of inoculum. The cultures were incubated at 37 °C and shaken at 250 rpm until the OD_{600} reached 0.7, then induced with 1 mM isopropyl-β-D-thiogalactopyranoside (IPTG) and the temperature was lowered to 25 °C for overnight protein expression. The cells were then harvested by centrifugation (5000 rcf, 30 min, 4 °C), the pellet was resuspended in 5 mL of 50 mM HEPES buffer at pH 7.0 and lysed by sonication (15 sec on 20 sec off, 15 cycles, 75% intensity). The lysate was clarified by centrifugation (10000 rcf, 40 min, 4 °C), the supernatant flash frozen in liquid nitrogen and lyophilised. The dry clarified cell lysate powder was stored at -20 °C. Total protein concentration was calculated using a standard Bradford assay.

Sequences

*Rs*TYR

```
MRIDFTINNGGDAAARYLTWAPSPLRLRLLDATPGPDVVATLSEDRQPNGGSIRFCATPDGNFTPTLKVPLPASGASVTV
YVRGKFGTPSQADGDVSIVVGGPASELGRLPVMVRVRKNANQLTPAERDRFISAMAQINNRGTGRFTDFRNMHVAGRADQ
QAHGGPGFLPWHRAYLLDLERELQAIDPAVTIPYWRFDRPAPNLFTTDFIGVPDALGTVSFSPANPLQFWATDGVQGILR
RQLGASPGAQAAPNILTEAQTLALGSAYRNFRGMQGNPHGSAHVSYFSGSISSIPTAAKDPLFFLLHCNVDRLWAKWQSQ
VGRYDANVAAAYDAGPTPTSLLAGHNLHDTLWPWNGIVTPPRPSTAPGGAMAGSSCVSAPGNAPRVSDMLDFQGVVSSSA
KLGFAYDDVPLPHHHHHH
```

*Cn*TYR

```
MVRKNASSLNPIERENFCKAVLTLKNTKIPGHALNRYDEFVAIHFGVTSRERANLPIGDGAHGNSGFLPWHREFLCRFEH
ALKSVDPTVSLPYWDWSSGDTSDTIDIFNDDFMGPAGTVNSGYFSGTGNSFNSNRPWIVHPSLDQTSPGQPPLGSTLIRN
SNLLSASTLNYLMDLGEMARDSLNESTYNAFRSTLEHPPHNHVHGVTVQGHMGWMTSPNDPIFFLHHANVDRLWAEWQRT
HPGSSNYTPNATEPYGVHLNDPMWPWQGADTTVTTRTHTDSNASLNTLLPSFSTADLVTPNDVLDHIQRCGPYDTDPISK
PKEFEKIPKEIIKEIIKDKEKEFGDKNPKEIIKEIIKDKEKEFGDKNPKEIIKEIIKDKEKEFGDKNPKEIKEIIKDKEK
EFGDKNPKEIKEIIKDKEKEFGDKNPKEIIETGDIKIENNKDVVEILSTPSTTVSSPKHPKEQSKETLEITNTLFDPLSK
INHRLDMLENEIKGTAFIKSTERPNITKRAISKNTSTKKTTRKKTKNTKNTMPKKSNTSKRKRISHHHHHH
```

*Cn*TYR-N201S

MVRKNASSLNPIERENFCKAVLTLKNTKIPGHALNRYDEFVAIHFGVTSRERANLPIGDGAHGNSGFLPWHREFLCRFEH
ALKSVDPTVSLPYWDWSSGDTSDTIDIFNDDFMGPAGTVNSGYFSGTGNSFNSNRPWIVHPSLDQTSPGQPPLGSTLIRN
SNLLSASTLNYLMDLGEMARDSLNESTYNAFRSTLEHPPHSHVHGVTVQGHMGWMTSPNDPIFFLHHANVDRLWAEWQRT
HPGSSNYTPNATEPYGVHLNDPMWPWQGADTTVTTRTHTDSNASLNTLLPSFSTADLVTPNDVLDHIQRCGPYDTDPISK
PKEFEKIPKEIIKEIIKDKEKEFGDKNPKEIIKEIIKDKEKEFGDKNPKEIIKEIIKDKEKEFGDKNPKEIKEIIKDKEK
EFGDKNPKEIKEIIKDKEKEFGDKNPKEIIETGDIKIENNKDVVEILSTPSTTVSSPKHPKEQSKETLEITNTLFDPLSK
INHRLDMLENEIKGTAFIKSTERPNITKRAISKNTSTKKTTRKKTKNTKNTMPKKSNTSKRKRISHHHHHH

1.10 Tyrosinase studies

1.10.1 Activity with dopamine

The activity of the three studied tyrosinases was tested using dopamine, a known substrate of this class of enzymes. The results from the tyrosinase screening are shown below in Figure S4.

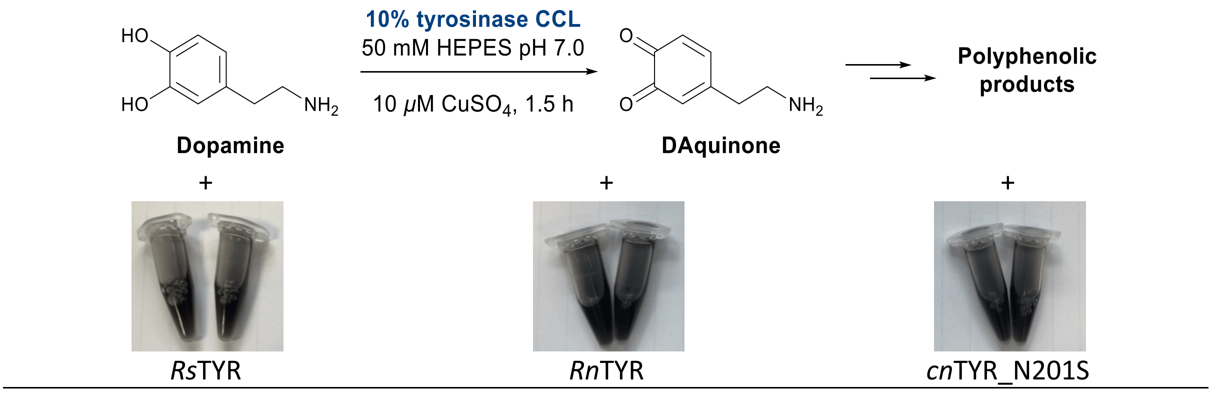

Figure S4. *Oxidation of dopamine using RsTYR, CnTYR and CnTYR_N201S. 2.5 mM dopamine, 10% clarified cell lysate and 10 μM $CuSO_4$ in 50 mM HEPES pH 7.0. Reaction shaken at 1000 rpm at 37 °C for 90 min.*

1.10.2 Co-solvent tolerance with MDA

The co-solvent tolerance of the selected TYRs in the oxidation of MDA was tested using CH_3CN and DMSO in different concentrations. The results are shown below in Figure S5.

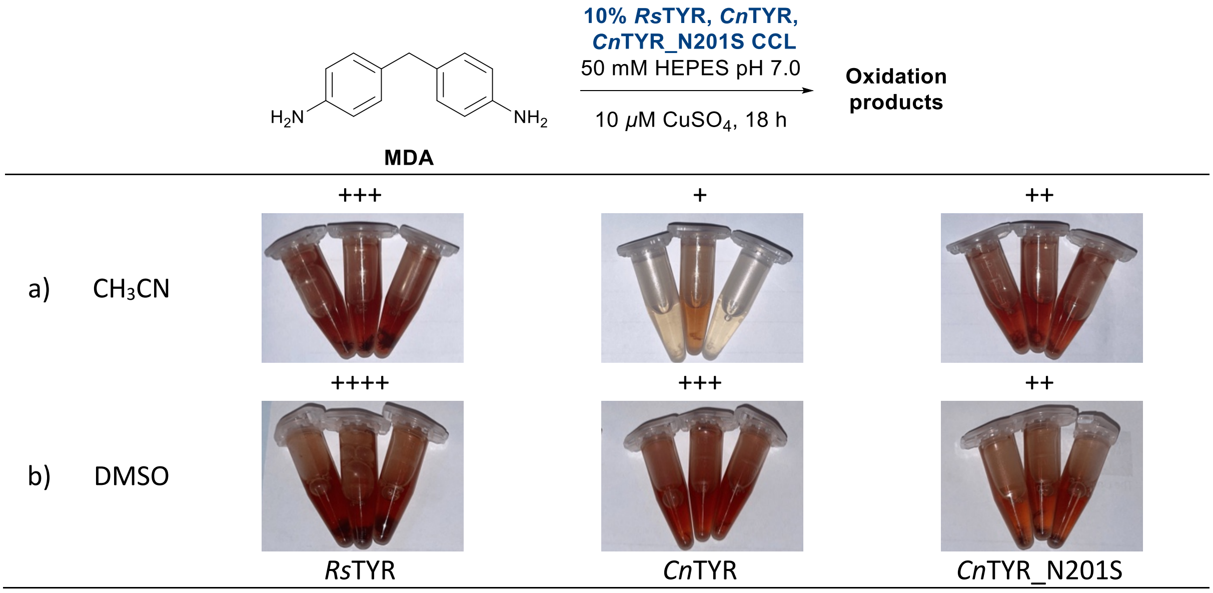

Figure S5. *Oxidation of MDA using RsTYR, CnTYR and CnTYR_N201S. 2.5 mM MDA, 10% clarified cell lysate, 10% cosolvent a) ACN b) DMSO, and 10 μM $CuSO_4$ in 50 mM HEPES pH 7.0. Reactions were shaken at 1000 rpm at 37 °C for 18 h. Guide: +++ represents a strong (dark) red coloration, ++ a medium red coloration, and + a pale red/orange coloration.*

1.10.3 *Rs*TYR control reactions with model substrates 1a-f

Control reactions with *Rs*TYR and substrates **1** were performed over 24 h. The results are shown below in Figure S6.

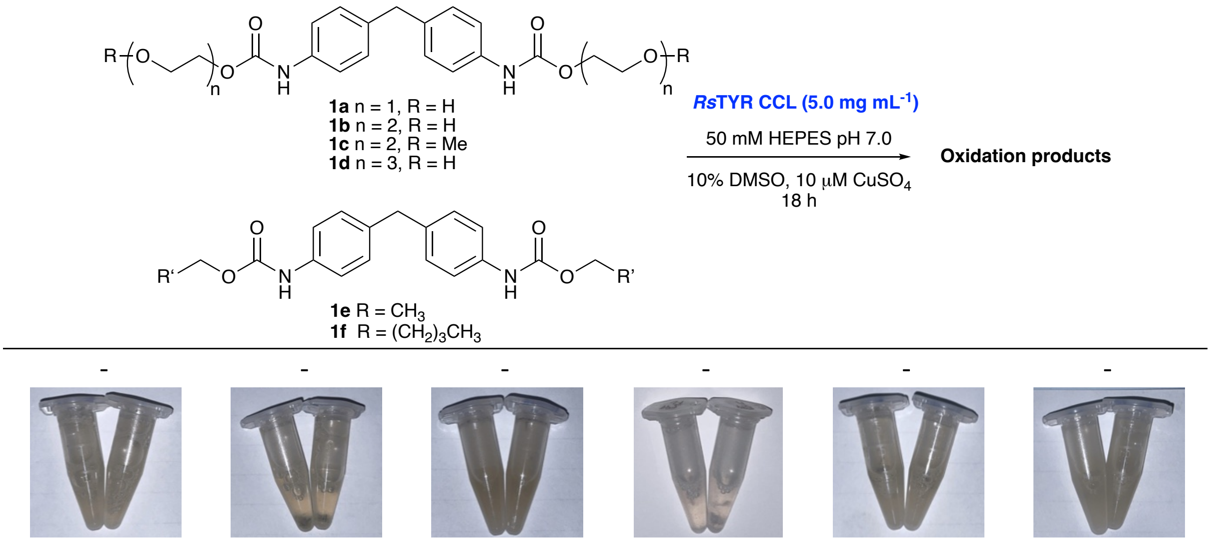

1a 1b 1c 1d 1e 1f

Figure S6*. Oxidation of* ***1a-f*** *using RsTYR. 2.5 mM* ***1****, 10% clarified cell lysate, 10% DMSO, and 10 μM $CuSO_4$ in 50 mM HEPES pH 7.0. Reactions were shaken at 1000 rpm at 37 °C for 18 h.*

1.11 Proposed mechanism of tyrosinase-mediated oxidation of MDA

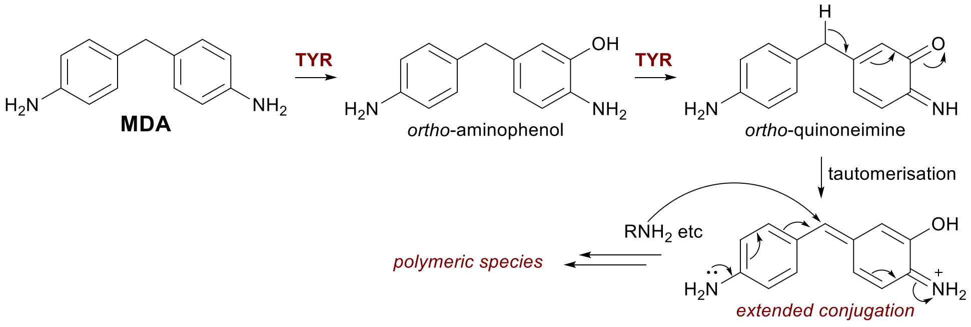

Scheme S1. *Proposed oxidation of MDA to the ortho-aminphenol and further oxidation to the ortho-quinoneimine followed by tautomerisation to give a conjugated species as a colorimetric readout for use in assays.*

1.12 List of hydrolases screened with the HTS colorimetric method

Table S3*. List of Enzymes screened (from the Jeffries-Hailes enzyme library) using the colorimetric assay.*

Entry	Enzyme	Bacterium/Species	Function	UniProtKB/GenBank
1	PET1 (*is*PETase)[11]	*Ideonella sakaiensis*	PETase	A0A0K8P6T7
2	PET2 (*is*PETase_W159H/ S238F) [12]	*Ideonella sakaiensis*	PETase	A0A0K8P6T7
3	*Pb*-PETase[13]	*Polyangium brachysporum* DSM7029	PETase	A0A0G3BI90
4	*Ad*-PETase[13]	*Acidovorax delafieldii BS-3*	PETase	BAB86909.1
5	*Bp*-PETase[13]	*Burkholderiales* bacterium	PETase	OGB27210.1
5s	s*Bp*-PETase[13]	*Burkholderiales* bacterium	PETase	-
6	umb-PETase[14]	Uncultured marine bacterium	PETase	C3RYL0

7	*Sp*-PETase	*Streptomyces pactum*	PETase	A0A1S6J2Q7
8	PET8	metagenomic PETase	PETase	MGYP000202667312
8s	PET8s	metagenomic PETase	PETase	MGYP000202667312
9	ThermoPETase[15]	*Ideonella sakaiensis*	PETase	A0A0K8P6T7
10	LCC-ICCG[16]	leaf-branch compost cutinase	PETase	G9BY57
11	DuraPETase[17]	*Ideonella sakaiensis*	PETase	A0A0K8P6T7
12	HotPETase[18]	*Ideonella sakaiensis*	PETase	A0A0K8P6T7
13	FAST-PETase[19]	*Ideonella sakaiensis*	PETase	A0A0K8P6T7
14	T-lipase[20]	*Thermomonospora curvata*	Lipase	D1A9G5
15	UMG-SP-1[21]	uncultured bacterium	Amidase	OP972509
16	UMG-SP-2[21]	uncultured bacterium	Amidase	OP972510
17	UMG-SP-3[21]	uncultured bacterium	Amidase	OP972511

Enzymes were cloned and expressed as previously described.[11-21]

1.13 Layout of Jeffries-Hailes library in 96-well plate used for the colorimetric assay

The numbers in the plate refer to the entries in Table S3. EVC = Empty vector cell, BL21(DE3) with pET-29a(+).

	**1**	**2**	**3**	**4**	**5**	**6**	**7**	**8**	**9**	**10**	**11**	**12**
**A**	1	1	1	2	2	2	3	3	3	4	4	4
**B**	5	5	5	5s	5s	5s	6	6	6	7	7	7
**C**	8	8	8	8s	8s	8s	9	9	9	10	10	10
**D**	11	11	11	12	12	12	13	13	13	14	14	14
**E**	15	15	15	16	16	16	17	17	17	EVC	EVC	EVC
**F**												
**G**												
**H**												

1.14 Procedure for the screening of enzyme panel with the colorimetric assay

1.14.1 Expression of enzymes in deep 96-well plate

E. coli BL21(DE3) or Shuffle T7 Express glycerol stocks of the hydrolase genes were used to inoculate 3 x 250 µL of LB media supplemented with 50 µg/mL kanamycin in a deep 96-well plate, which was incubated at 37 °C overnight at 1800 rpm in a thermomixer. The OD_{600} of the overnight cultures was measured using a plate reader, averaged across the plate and a fresh deep 96-well plate containing 250 µL/well MagicMedia® supplemented with 50 µg/mL kanamycin was inoculated using an amount of overnight culture to reach an OD_{600} of 0.1. The plate was incubated at 37 °C at 1800 rpm in a thermomixer and after 3 h the temperature was lowered to 25 °C and incubated for a further 24 h for protein expression. The plate was then centrifuged at 4000 rcf for 20 min at 4 °C and the media was gently poured off. Excess media was tapped onto a paper towel and the pellets were resuspended and lysed in 150 µL 50 mM KPi pH 7.4 containing 1X BugBuster® lysis agent for 30 min at 1200 rpm using a silicon sealing mat. The plate was then centrifuged at 5000 rcf for 40 min at 4 °C to obtain clarified cell lysates.

1.14.2 Screening of enzymes

To 35 µL 50 mM KPi pH 7.4, 100 µL of CCL from 1.13.1 were added to a new 96-well plate. Then 15 µL of a 25 mM stock solution in DMSO of **1a**-**d** was added to initiate the reaction, and the plate was incubated at 37 °C for 24 h at 1000 rpm. 15 µL of 50 mg mL^{-1} *Rs*TYR in 50 mM KPi buffer pH 7.4 and 2.0 µL of 825 µM $CuSO_4$ in water were added to the reaction to start the oxidation of MDA generated by the enzymatic hydrolysis. The reaction was incubated at 37 °C and 1000 rpm for a further 24 h.

1.15 Batch growth of urethanases

BL21(DE3) glycerol stocks of the selected genes in pET-29a(+) obtained from GenScript were used to inoculate 10 mL of LB media supplemented with 50 µg/mL kanamycin, which was incubated at 37 °C overnight at 250 rpm. 1mL inoculum of the overnight cultures was then used to inoculate 100 mL of LB or TB media with 50 µg/mL kanamycin. The cultures were incubated at 37 °C and shaken at 250 rpm until the OD_{600} reached 0.4-0.7, then induced with 0.5 mM IPTG and the temperature was lowered to 25 °C for overnight protein expression. The cells were then harvested by centrifugation (5000 rcf, 30 min, 4 °C), the pellet was resuspended in 50 mM KPi buffer at pH 7.5 and lysed by sonication (15 sec on 20 sec off, 15 cycles, 75% intensity). The lysate was clarified by centrifugation (10000 rcf, 40 min, 4 °C) and the supernatant was divided in 200-500 µL aliquots, flash frozen and stored at -20 °C. Total protein concentration was calculated using a standard Bradford assay and the target enzyme concentration was calculated by gel densitometry using ImageJ software.

1.16 General procedure for the enzymatic hydrolysis of substrates 1

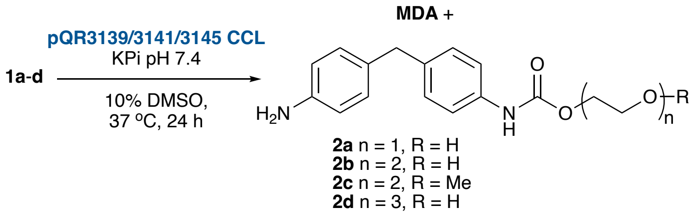

To 180 µL CCL of pQR3137-3145 enzymes in 50 mM KPi buffer pH 7.4 (~10 mg mL^{-1} total protein content) in a microcentrifuge tube, 20 µL of a 25 mM stock solution in DMSO of **1a-d** was added to initiate the reaction. The reaction was incubated at 37 °C and 1000 rpm in a thermomixer for 24 h.

***Rs*TYR tandem reaction.** After 24 h, 20 µL of 50 mg mL^{-1} *Rs*TYR in 50 mM KPi buffer pH 7.4 and 2.0 µL of 1.0 mM $CuSO_4$ in water were added to the reaction to start the oxidation of MDA generated by the enzymatic hydrolysis. The reaction was incubated at 37 °C and 1000 rpm for a further 24 h.

HPLC analysis. The reaction was then quenched with 200 µL DMSO, centrifuged at maximum speed for 5 min and filtered through a 0.45 µm syringe filter. It was then analysed by HPLC to calculate the product conversion.

1.17 HPLC calibration curves

The concentration of MDA and starting materials **1a-e** was determined via analytical HPLC using product standards, with UV detection at 260 nm using the following **method A**:

- Column: InfinityLab Poroshell 120 EC-C18, 4.6 x 150 mm, 4 µm fitted with a guard column.
- Gradient method: **solvent A** 10 mM NH_4Ac pH 5.0, **solvent B** MeOH, flow 1.0 mL min^{-1}, linear gradient to 25-95% B over 15 min, hold at 95% B for 3 min, linear gradient to 25% B in 30 s and hold for 2.5 min at 25% B.

Each calibration point was prepared in triplicate.

1.17.1 MDA

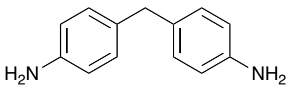

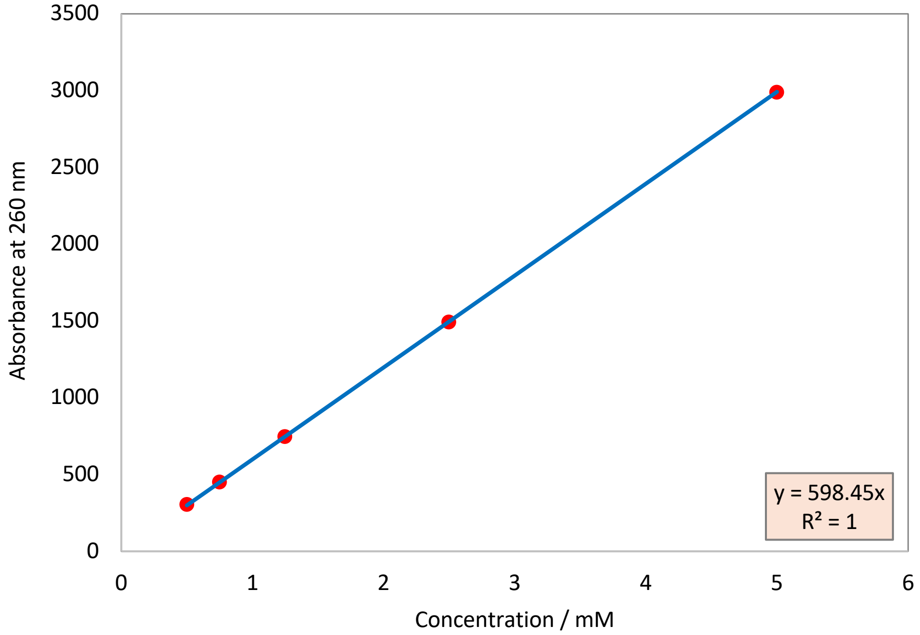

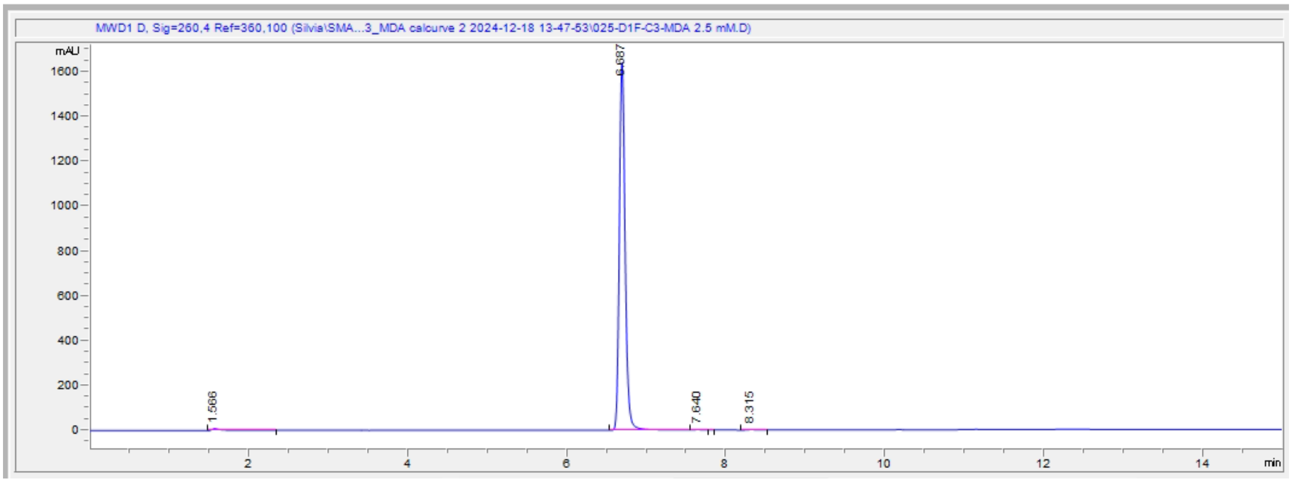

Retention time: 6.7 min

1.17.2 Bis(2-hydroxyethyl) (methylenebis(4,1-phenylene))dicarbamate 1a

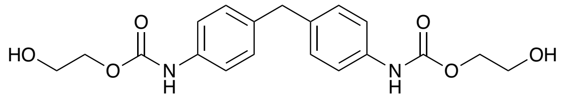

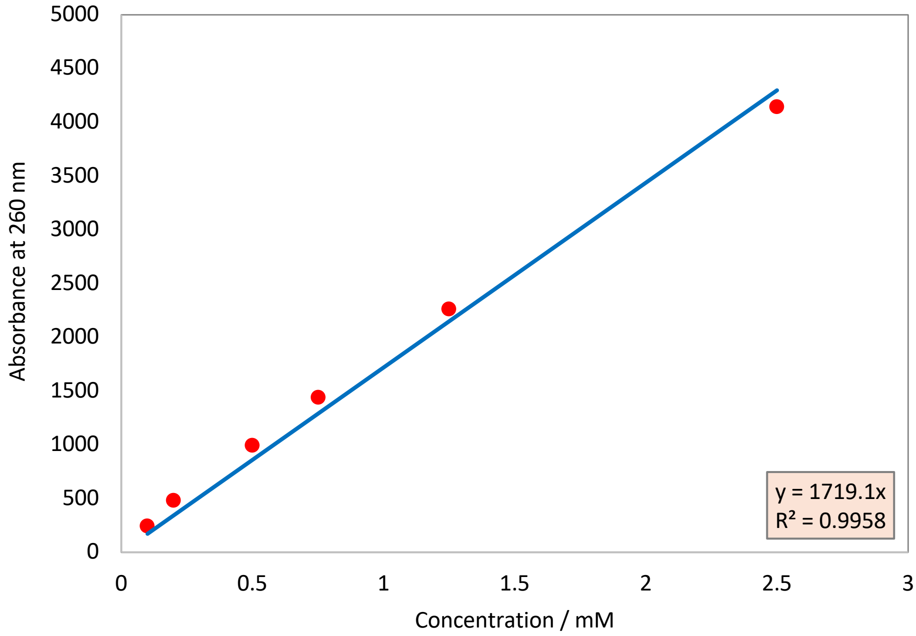

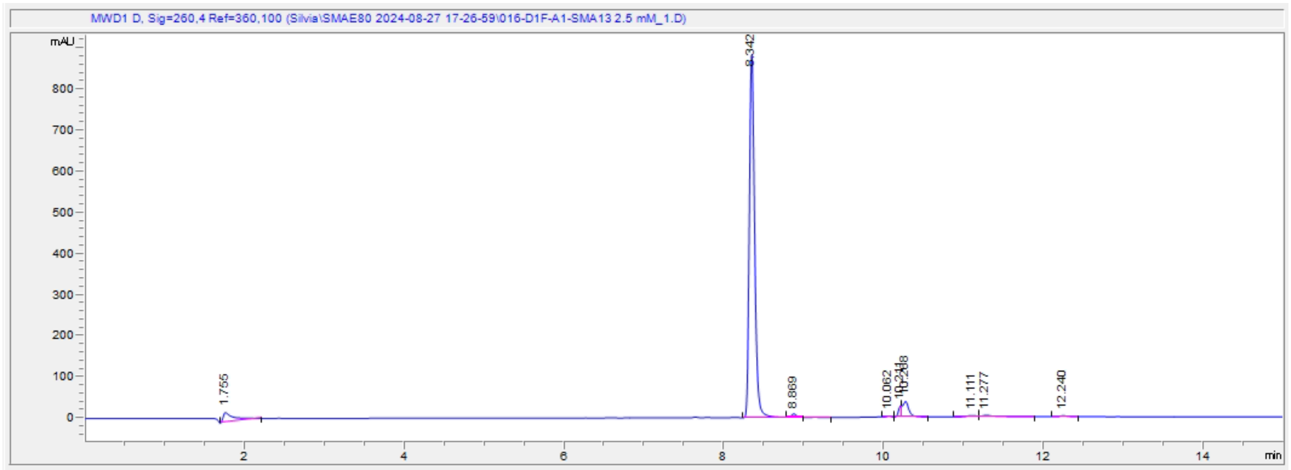

Retention time: 8.3 min.

1.17.3 Bis(2-(2-hydroxyethoxy)ethyl) (methylenebis(4,1-phenylene))dicarbamate 1b

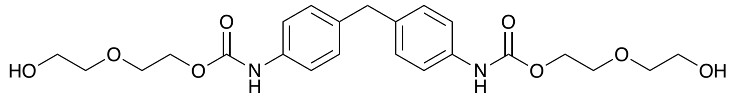

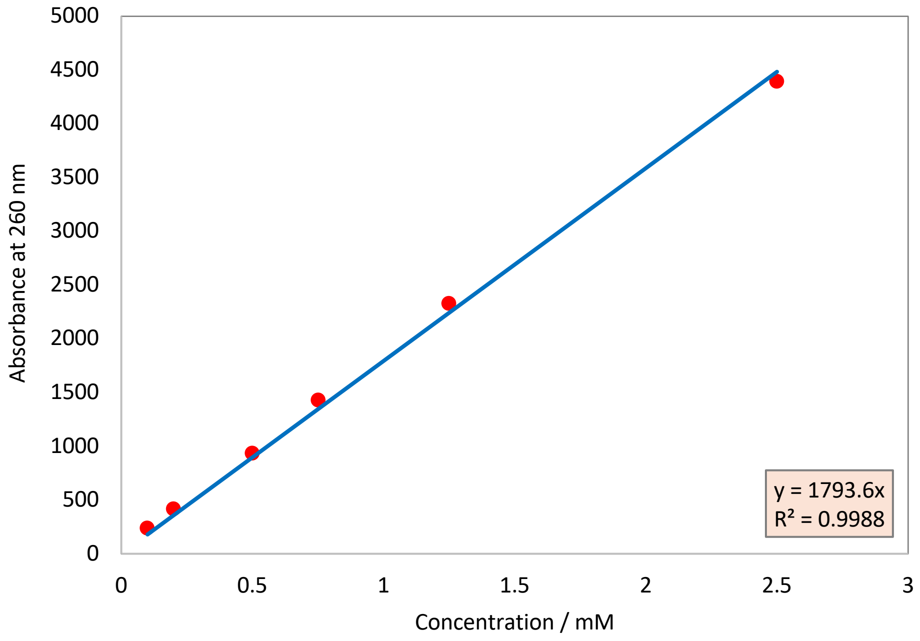

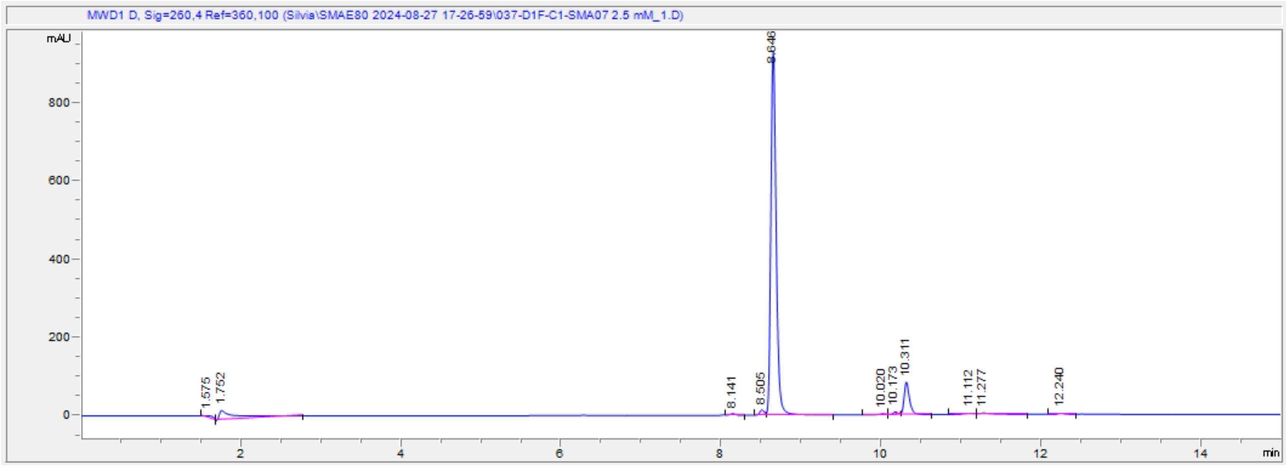

Retention time: 8.7 min.

1.17.4 Bis(2-(2-methoxyethoxy)ethyl) (methylenebis(4,1-phenylene))dicarbamate 1c

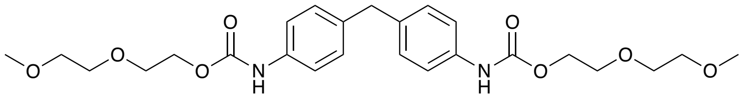

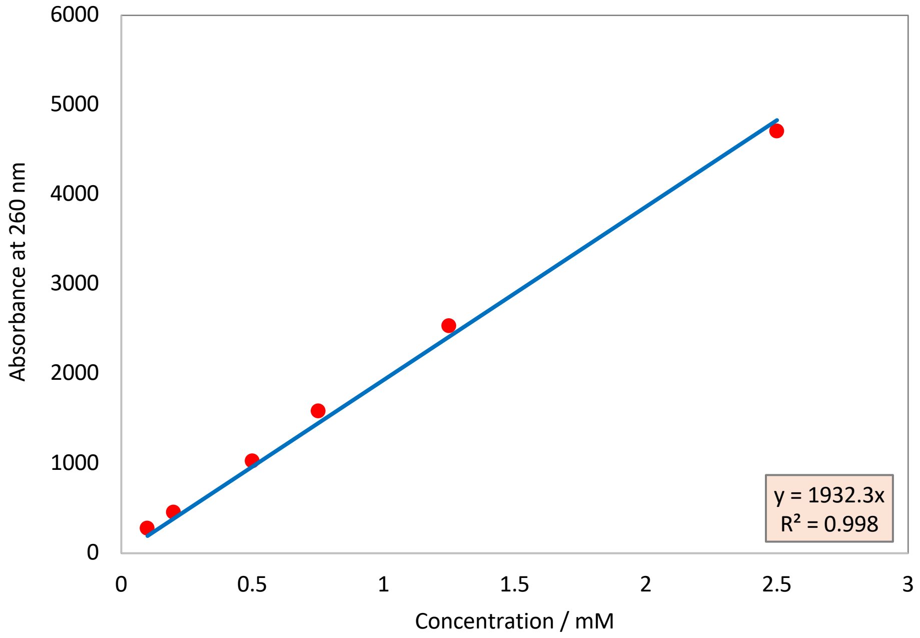

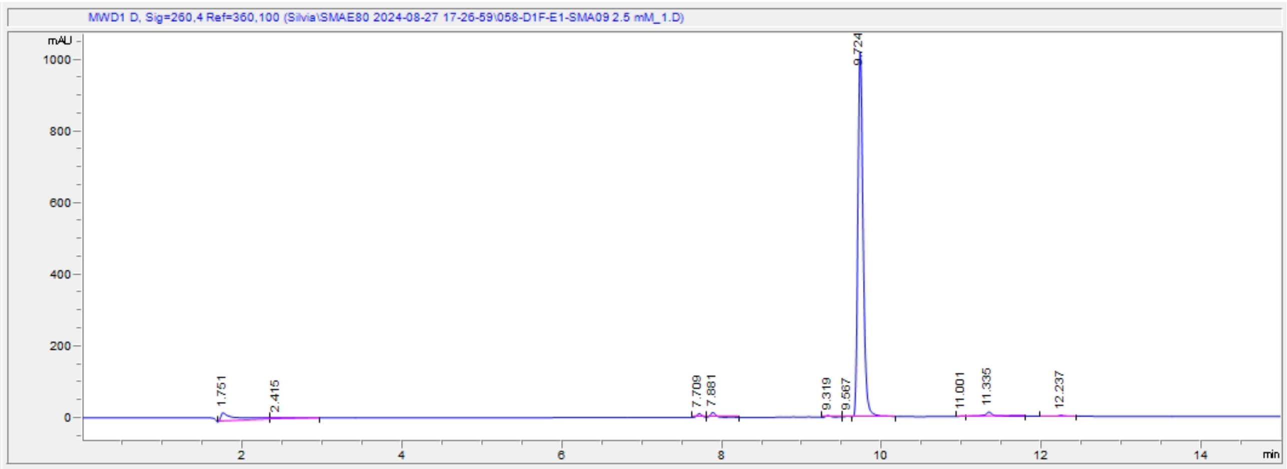

Retention time: 9.7 min.

1.17.5 Bis(2-(2-(2-hydroxyethoxy)ethoxy)ethyl) (methylenebis(4,1-phenylene))dicarbamate

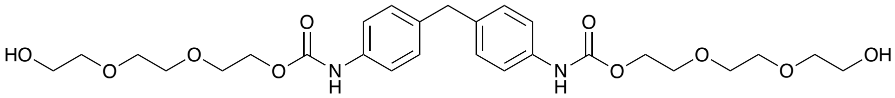

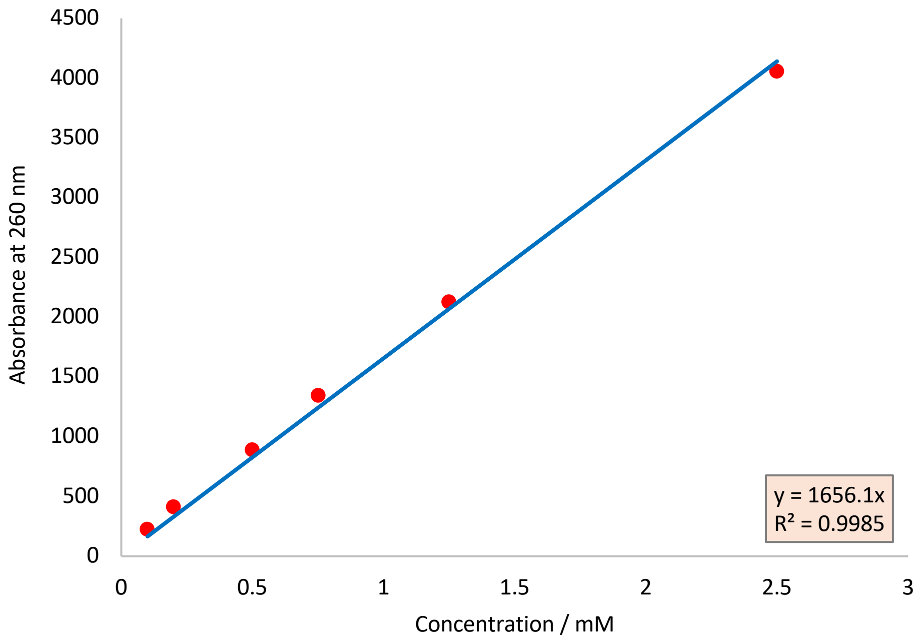

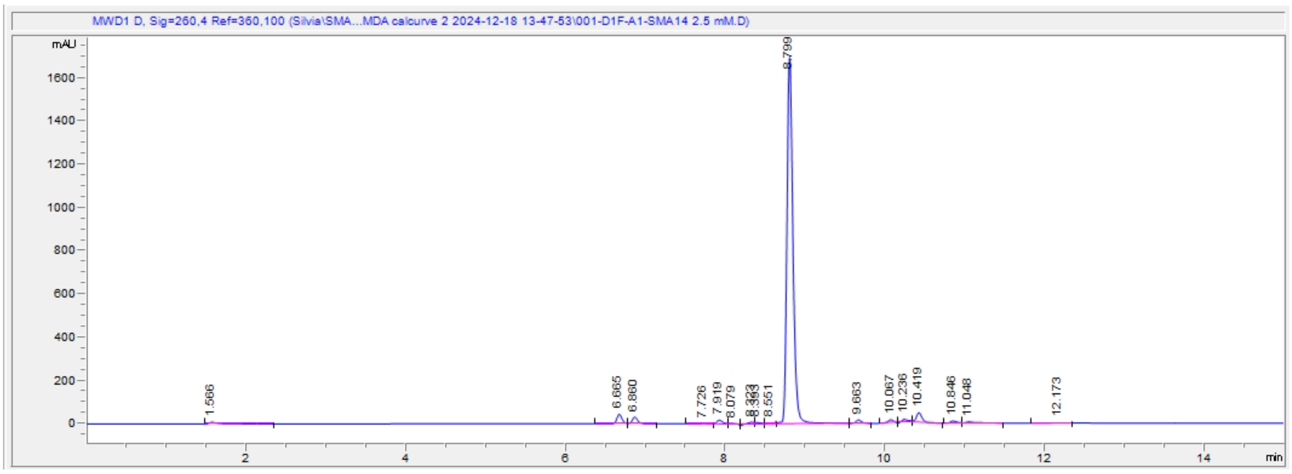

Retention time 8.8 min.

1.17.6 Diethyl (methylenebis(4,1-phenylene))dicarbamate 1e

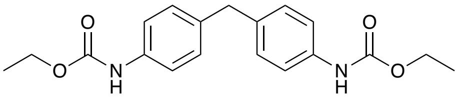

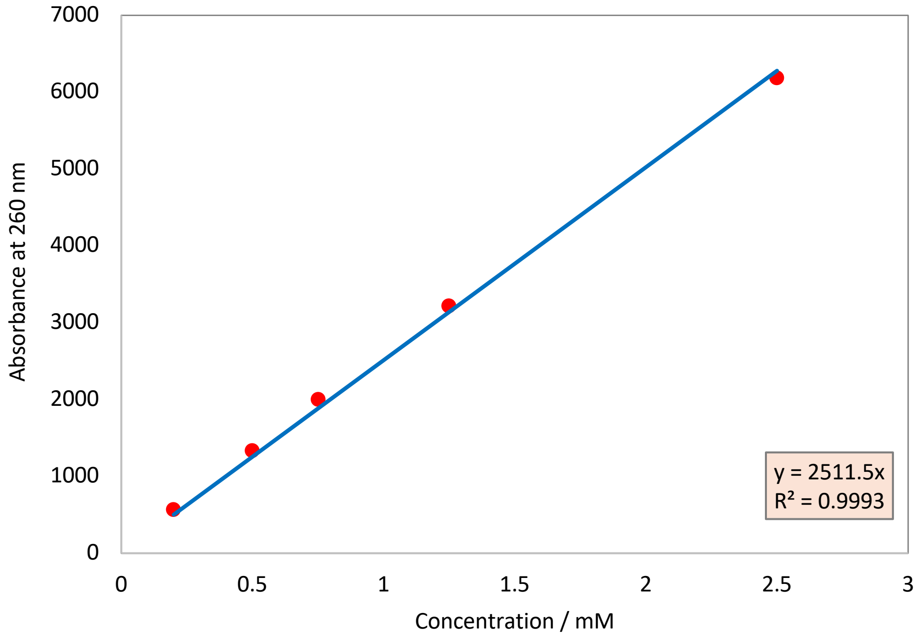

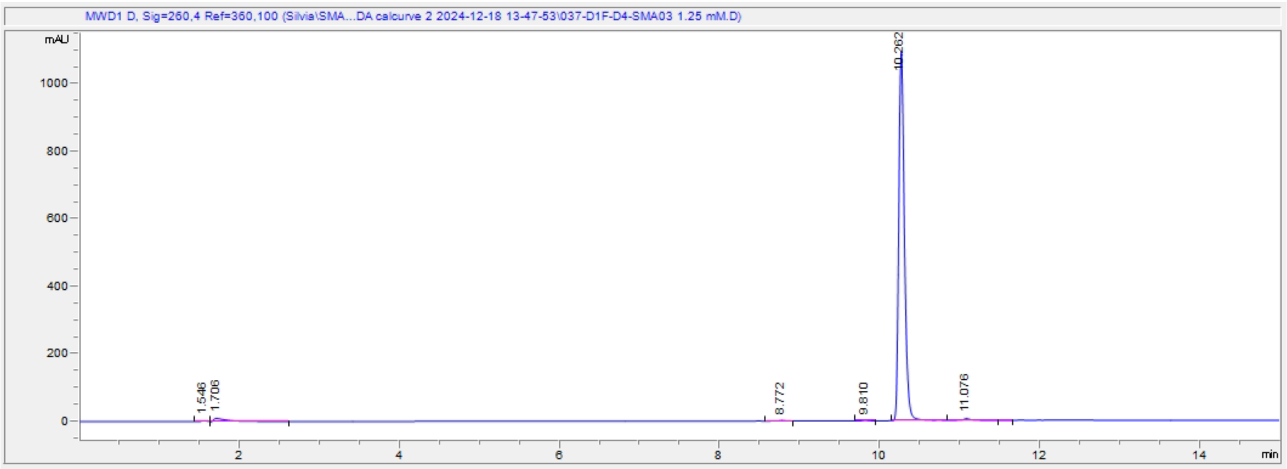

Retention time: 10.3 min.

2 Chemistry

2.1 General methods

Reagents and solvents were used as supplied from the vendor without further purification. Thin layer chromatography plates (Merk, silica gel 60 F254, aluminium backed) were viewed under UV light and stained using KMnO4 developed using heat. $MgSO_4$ (Sigma Aldrich, anhydrous ≥ 98.0 %) was used as the drying agent. Column chromatography was performed on silica gel for flash chromatography (Sigma Aldrich, 40-63 µm particle size, 60 Å pore size). Products were characterised by ^{1}H NMR and ^{13}C NMR spectra obtained from one of the following: a) Bruker (Germany) Avance Neo 400 (^{1}H 400 MHz, ^{13}C 101 MHz) at 300 K; b) Bruker (Germany) Avance Neo 500 (^{1}H 500 MHz, ^{13}C 126 MHz) at 300 K. Chemical shifts are reported in ppm relative to the reference peaks of the indicated deuterated solvents. Coupling constants (*J*) are reported in Hz, multiplicities are specified as singlet (s), doublet (d), triplet (t), quartet (q), pentet (p), sextet (sx), septet (h), multiplet (m). Assignment of peaks was aided by two-dimensional NMR spectroscopy (COSY, HSQC, HMBC and NOESY).

HPLC and LC-MS analysis was carried out using one of the following: a) Agilent 1260 Infinity II HPLC system comprising a G7167A multisampler, a G7112B bin pump, a G7116A multi-column thermostat (MCT) and a G7165A muti-wavelength detector (MWD), equipped with an Agilent Poroshell 120 EC-C18 column (100 mm × 4.6 mm) with the UV detector at 1 = 254 nm, 2 = 240 nm, 3 = 210 nm; b) Waters Acquity UPLC connected to a TUV detector and SQD2 operating in electrospray ionisation methods in the positive mode (ESI+). High resolution mass spectra were acquired on a Waters LCT Premier XE instrument, using ESI+ mode.

2.2 General synthesis of polyether model substrates 1a-d

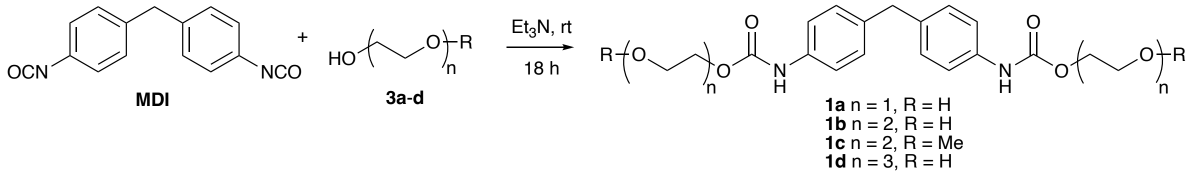

The selected n-ethylene glycol **3** (10-40 eq.), Et_3N (0.1 eq.) and solvent (20 mL) were stirred at room temperature (rt). To this solution, methylene diphenyl diisocyanate (MDI) (250 mg, 1.0 eq.) in 20 mL of solvent was added dropwise. The reaction mixture was stirred for 18 h and monitored by TLC analysis. Upon completion, the reaction for **1a** was quenched with water and the product filtered, washed with water and dried in a 30 °C oven overnight or *in vacuo*. Reactions to generate **1b-d** were quenched with water, extracted with EtOAc (3 x 10 mL), washed with saturated $NaHCO_3$ (5 x 20 mL) to remove the excess glycol, and dried in a 30 °C oven overnight or *in vacuo*.

2.2.1 Bis(2-hydroxyethyl) (methylenebis(4,1-phenylene))dicarbamate 1a[22]

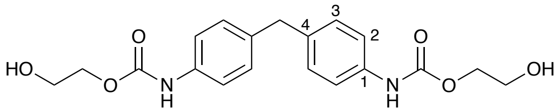

Synthesised using MDI (250 mg, 1.00 mmol), ethylene glycol **3a** (2.23 mL, 40.0 mmol) and acetone as the solvent to give **1a** (284 mg, 76% yield) as a white amorphous solid. Analytical HPLC retention time: 8.3 min (HPLC **method A**), Rf = 0.73 (EtOAc/EtOH/hexane, 3:1:1). ^{1}H NMR (500 MHz; DMSO-d_6) δ 9.58 (2H, s, 2 x NH), 7.35 (4H, d, *J* = 8.1 Hz, 2 x 2-H), 7.13 – 7.06 (4H, m, 2 x 3-H), 4.80 (2H, t, *J* = 5.2 Hz, 2 x OH), 4.07 (4H, t, *J* = 5.2 Hz, 2 x CH_2OCO), 3.78 (2H, s, CH_2Ar), 3.60 (4H, q, *J* = 5.2 Hz, 2 x CH_2OH); ^{13}C NMR (126 MHz; DMSO-d_6) δ 153.7, 137.2, 135.5, 128.9, 118.4, 65.9, 59.3, 40.0; *m/z* [ES+] 375 ($[M+H]^+$); *m/z* [HRMS ES+] found $[M+H]^+$ 375.1545. $[C_{19}H_{23}N_2O_6+H]^+$ requires 375.1551.

2.2.2 Bis(2-(2-hydroxyethoxy)ethyl) (methylenebis(4,1-phenylene))dicarbamate 1b[22]

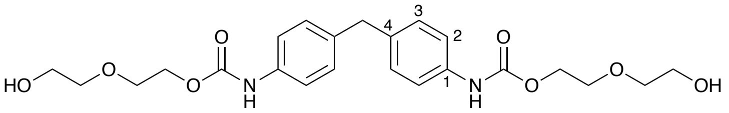

Synthesised using MDI (250 mg, 1.00 mmol), diethylene glycol **3b** (0.946 mL, 10.0 mmol) and CH_2Cl_2 as the solvent to give **1b** (275 mg, 56% yield) as a viscous yellow oil. Analytical HPLC retention time: 8.6 min (HPLC **method A**); Rf = 0.83 (CH_2Cl_2/MeOH 95:5); ν_{max} (neat)/cm^{-1} 3319, 2947, 1695, 1533; ^{1}H NMR (500 MHz; DMSO-d$_6$) δ 9.64 (2H, s, 2 x NH), 7.35 (4H, d, *J* = 8.4 Hz, 2 x 2-H), 7.09 (4H, d, *J* = 8.4 Hz, 2 x 3-H), 4.61 (2H, t, *J* = 5.4 Hz, 2 x OH), 4.19 – 4.17 (4H, m, 2 x CH_2OCO), 3.78 (2H, s, CH_2Ar), 3.65 – 3.60 (4H, m, 2 x C*H*$_2$CH$_2$OCO), 3.54 – 3.47 (4H, m, 2 x C*H*$_2$OH), 3.47 – 3.43 (4H, m, 2 x C*H*$_2$CH$_2$OH); ^{13}C NMR (126 MHz; DMSO-d$_6$) δ 153.5, 137.1, 135.5, 128.9, 118.3, 72.3, 68.7, 63.5, 60.2, 39.6; *m/z* [ES+] 463 ([M+H]$^+$); *m/z* [HRMS ES+] found [M+H]$^+$ 463.2069. [$C_{23}H_{31}N_2O_8$+H]$^+$ requires 463.2075.

2.2.3 Bis(2-(2-methoxyethoxy)ethyl) (methylenebis(4,1-phenylene))dicarbamate 1c

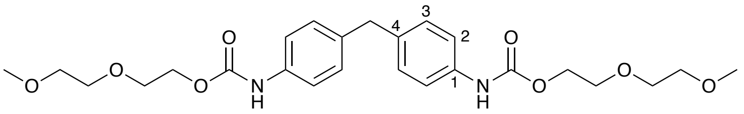

Synthesised using MDI (250 mg, 1.00 mmol), diethylene glycol monomethyl ether **3c** (1.65 mL, 14.0 mmol) and CH_2Cl_2 as the solvent to give **1c** (0.343 g, 70% yield) as a viscous yellow oil. Analytical HPLC retention time: 9.7 min (HPLC **method A**); Rf = 0.75 (hexane/EtOAc 1:4); ν_{max} (neat)/cm^{-1} 3249, 2855, 1705, 1529; ^{1}H NMR (500 MHz; $CDCl_3$) δ 7.30 – 7.24 (4H, m, 2 x 2-H), 7.09 (4H, d, *J* = 8.3 Hz, 2 x 3-H), 6.70 (2H, s, NH), 4.35 – 4.29 (4H, m, 2 x CH_2OCO), 3.88 (2H, s, CH_2Ar), 3.76 – 3.71 (4H, m, 2 x C*H*$_2$CH$_2$OCO), 3.69 – 3.63 (4H, m, C*H*$_2$CH$_2$OCH$_3$), 3.58 – 3.54 (4H, m, 2 x C*H*$_2$OCH$_3$), 3.38 (6H, s, 2 x OCH_3); ^{13}C NMR (126 MHz, $CDCl_3$) δ 153.5, 136.5, 136.0, 129.6, 119.0, 72.0, 70.6, 69.6, 64.3, 59.2, 40.7; *m/z* [ES+] 491 ([M+H]$^+$); *m/z* [HRMS ES+] found [M+H]$^+$ 491.2387. [$C_{25}H_{35}N_2O_8$+H]$^+$ requires 491.2388.

2.2.4 Bis(2-(2-(2-hydroxyethoxy)ethoxy)ethyl) (methylenebis(4,1-phenylene))dicarbamate 1d

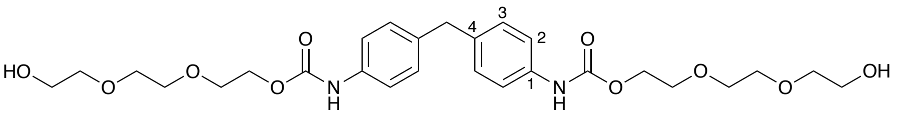

Synthesised using MDI (250 mg, 1.00 mmol), triethylene glycol **3d** (1.36 mL, 10.0 mmol) and toluene as the solvent to give **1d** (385 mg, 70% yield) as a viscous oil. Analytical HPLC retention time: 8.8 min (HPLC **method A**); Rf = 0.75 (hexane/EtOAc 1:4); ν_{max} (neat)/cm^{-1} 3275, 2922, 1707, 1530; ^{1}H NMR (500 MHz; DMSO-d$_6$) δ 9.65 (2H, s, 2 x NH), 7.35 (4H, d, *J* = 8.2 Hz, 2 x 2-H), 7.12 – 7.06 (4H, m, 2 x 3-H), 4.57 (2H, t, *J* = 5.4 Hz, 2 x OH), 4.20 – 4.14 (4H, m, 2 x CH_2OCO), 3.78 (2H, s, CH_2Ar), 3.65 – 3.61 (4H, m, 2 x C*H*$_2$CH$_2$OCO), 3.57 – 3.50 (8H, m, 4 x OCH_2), 3.50 – 3.44 (4H, m, 2 x C*H*$_2$OH), 3.43 – 3.39 (4H, m, 2 x C*H*$_2$CH$_2$OH); ^{13}C NMR (126 MHz; DMSO-d$_6$) δ 153.5, 137.1, 135.5, 128.9, 118.4, 72.4, 69.78, 69.76, 68.7, 63.5, 60.2, 39.8; *m/z* [ES+] 551 ([M+H]$^+$); *m/z* [HRMS ES+] found [M+H]$^+$ 551.2571. [$C_{27}H_{39}N_2O_{10}$ +H]$^+$ requires 551.2599.

2.3 Diethyl (methylenebis(4,1-phenylene))dicarbamate 1e[22]

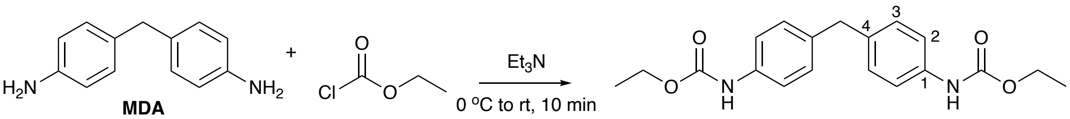

MDA (200 mg, 2.52 mmol) and Et_3N (365 µL, 2.63 mmol) were added to CH_2Cl_2 (5 mL) and stirred at 0 °C. Ethyl chloroformate (250 µL, 2.63 mmol) was then added dropwise and the reaction was warmed to rt. Upon

completion (10 min), the reaction was quenched with water (20 mL), extracted with CH_2Cl_2 (3 x 10 mL), and washed with brine (10 mL). The combined organic layers were dried over $MgSO_4$ and concentrated under pressure. The crude product was purified by column chromatography (7:3 hexane/EtOAc) to give **1e** (151 mg, 44% yield) as a white amorphous solid. M.p. 123-127 °C; Analytical HPLC retention time: 10.3 min (HPLC **method A**); Rf = 0.47 (hexane/EtOAc 2:1); ν_{max} (neat)/cm^{-1} 3313, 1693, 1525, 1413; ^{1}H NMR (500 MHz; CD_3OD) δ 7.31 (4H, d, J = 8.3 Hz, 2 x 2-H), 7.08 (4H, d, J = 8.3 Hz, 2 x 3-H), 4.15 (4H, q, J = 7.1 Hz, 2 x OCH_2), 3.84 (2H, s, CH_2Ar), 1.28 (6H, t, J = 7.1 Hz, 2 x CH_3); ^{13}C NMR (126 MHz; CD_3OD) δ 156.2, 138.2, 137.6, 130.1, 120.1, 61.8, 41.4, 14.9; *m/z* [ES+] 343 ([M+H]$^+$); *m/z* [HRMS ES+] found [M+H]$^+$ 343.1640. [$C_{19}H_{23}N_2O_4$ +H]$^+$ requires 343.1652.

2.4 Synthesis of dihexyl (methylenebis(4,1-phenylene))dicarbamate 1f[23]

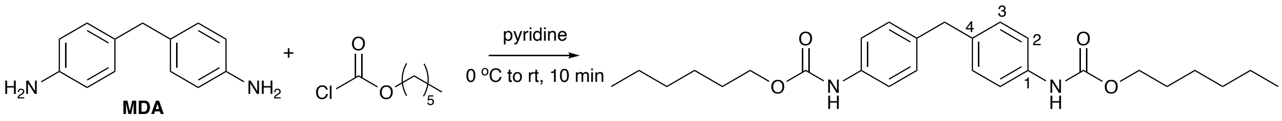

MDA (500 mg, 2.52 mmol) and pyridine (611 µL, 7.56 mmol) were added to dry DMF (8 mL) under argon and stirred at 0 °C. Hexyl chloroformate (824 µL, 5.04 mmol) was then added dropwise and the reaction was warmed to rt. After 10 min, the reaction was complete by TLC analysis and EtOAc (20 mL) was added and the product was washed with 5% HCl (2 x 20 mL). Small amounts of sat. $NaHCO_3$ solution were used to eliminate emulsion formation. Then the combine aqueous layers were extracted with EtOAc (2 x 20 mL) and further washed with sat. $NaHCO_3$, dried over $MgSO_4$ and concentrated under pressure. To remove trace amounts of DMF, hexane was added and the product precipitated, which was filtered and rinsed with cold hexane to afford **1f** (1.03 g, 75% yield) as colourless crystals. M.p. 103-107 °C; Rf = 0.78 (hexane/EtOAc 2:1); ν_{max} (neat)/cm^{-1} 3309, 2953, 1697, 1533; ^{1}H NMR (500 MHz; DMSO-d_6) δ 9.49 (2H, s, 2 x NH), 7.34 (4H, d, J = 8.1 Hz, 2 x 2-H), 7.11 – 7.05 (4H, m, 2 x 3-H), 4.04 (4H, t, J = 6.6 Hz, 2 x CH_2OCO), 3.78 (2H, s, 2H, CH_2Ar), 1.64 – 1.54 (4H, m, 4H, 2 x C$H_2$$CH_2OCO$), 1.39 – 1.31 (4H, m, 4H, 2 x C$H_2$$CH_2CH_2OCO$), 1.31 – 1.21 (8H, m, 4 x CH_2), 0.87 (6H, t, J = 7.0 Hz, 2 x CH_3); ^{13}C NMR (126 MHz, DMSO-d_6) δ 153.7, 137.2, 135.4, 128.8, 118.3, 64.1, 39.8, 30.9, 28.5, 25.1, 22.1, 13.9; *m/z* [ES+] 455 ([M+H]$^+$); *m/z* [HRMS ES+] found [M+H]$^+$ 455.2882. [$C_{27}H_{39}N_2O_4$ +H]$^+$ requires 455.2904.

2.5 NMR spectra

2.5.1 Bis(2-hydroxyethyl) (methylenebis(4,1-phenylene))dicarbamate 1a

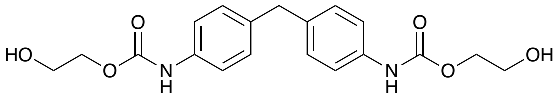

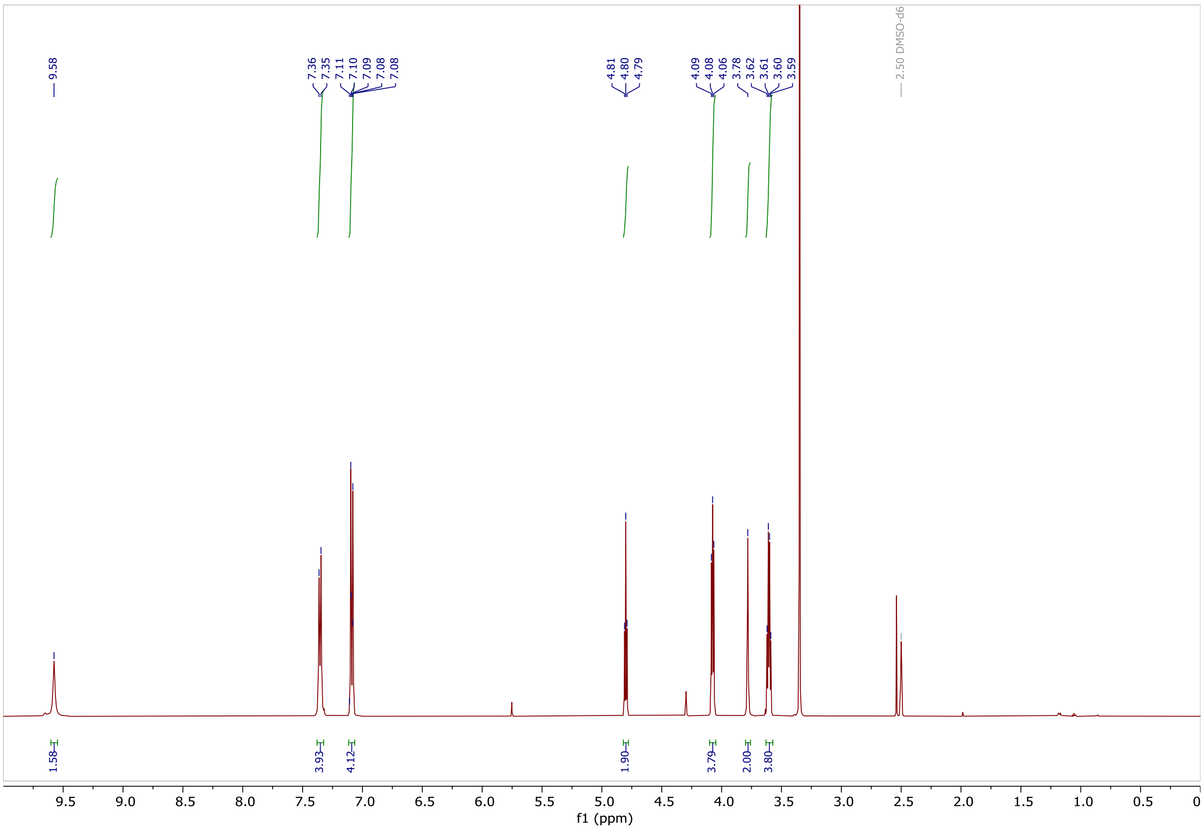

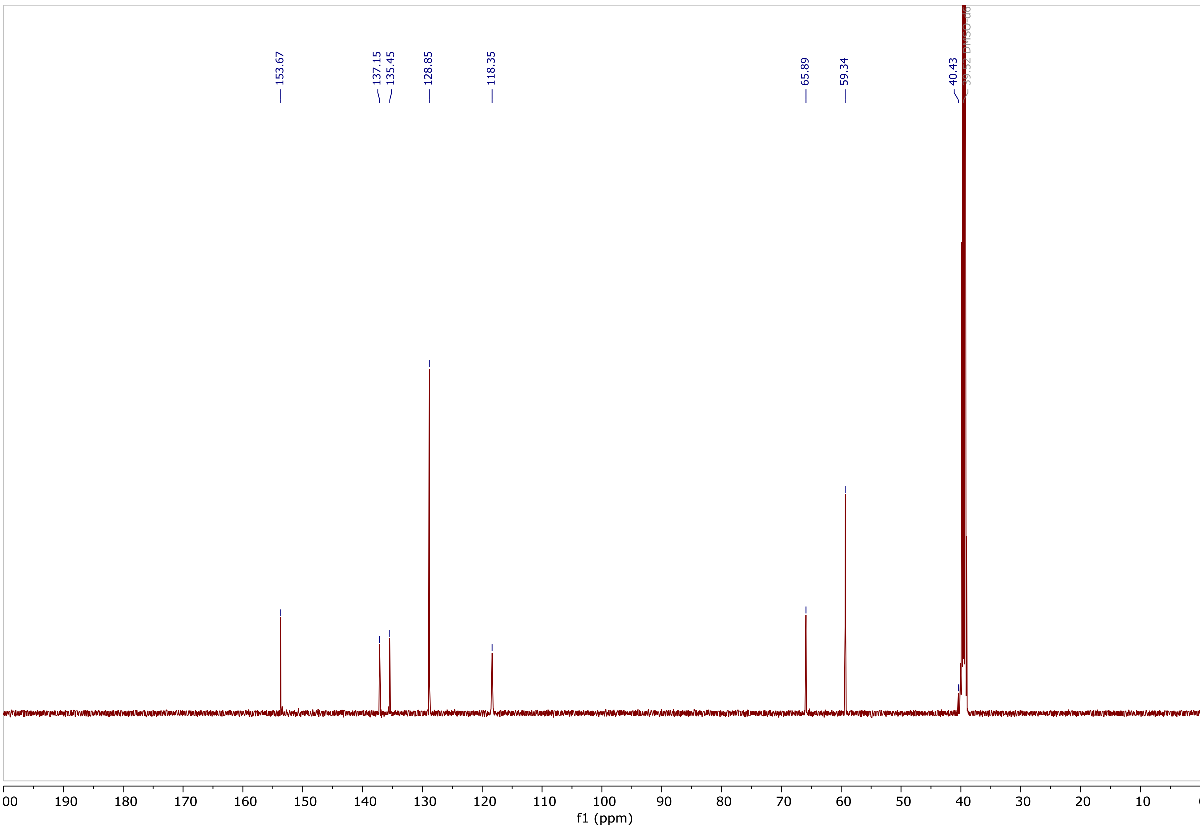

2.5.2 Bis(2-(2-hydroxyethoxy)ethyl) (methylenebis(4,1-phenylene))dicarbamate 1b

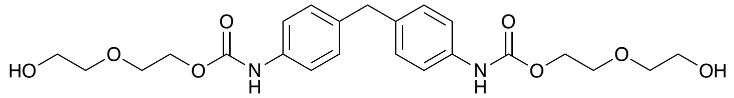

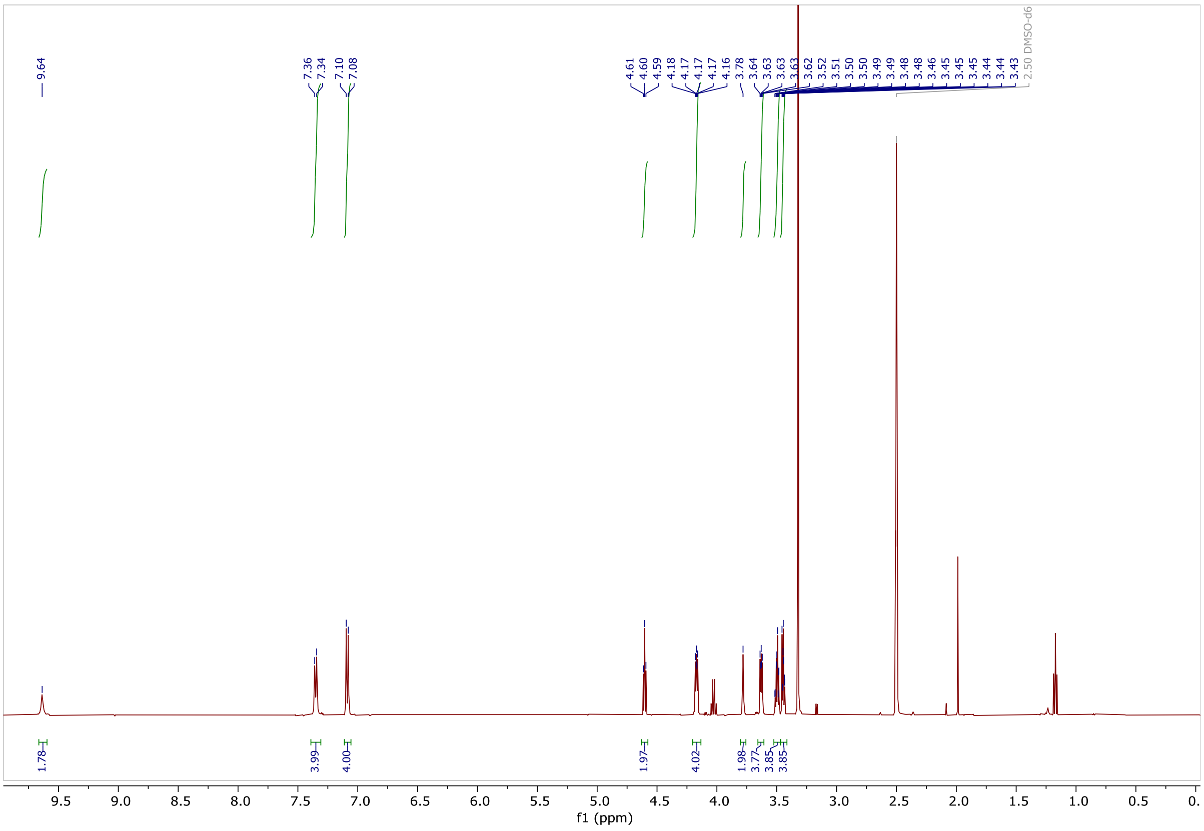

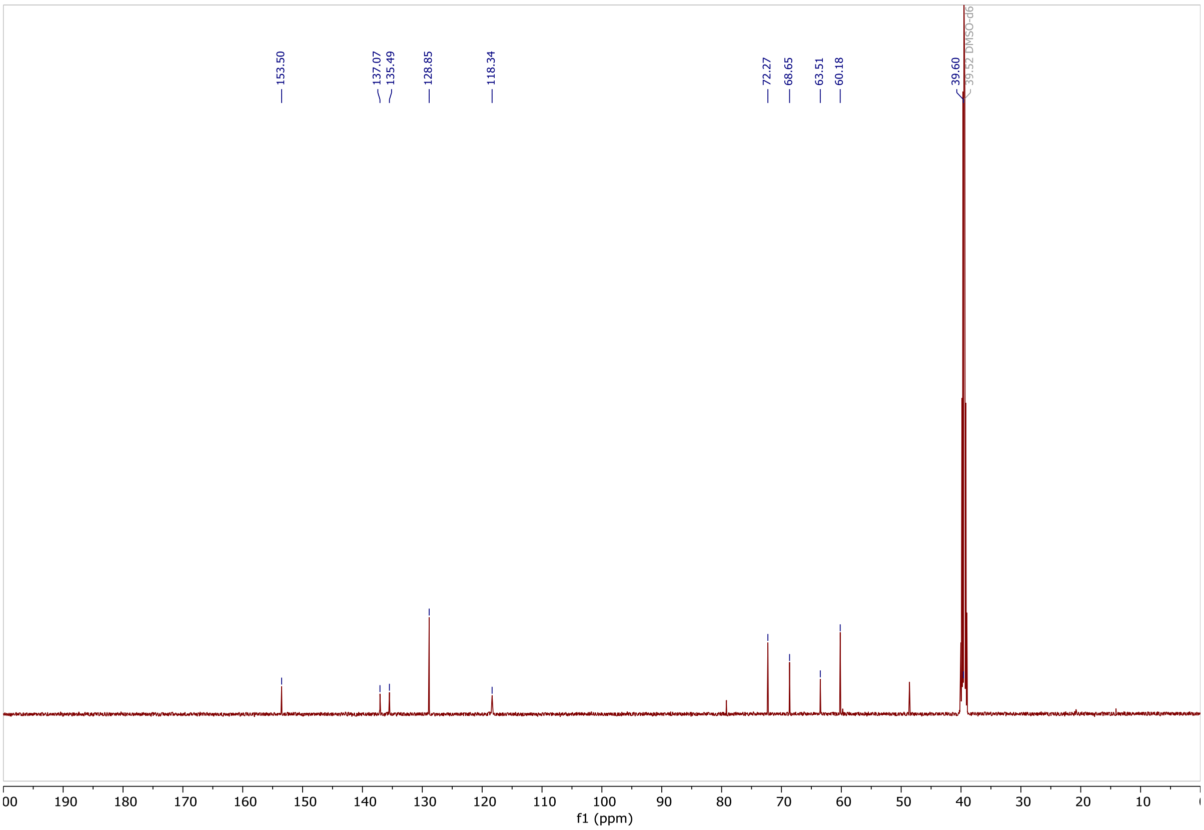

2.5.3 Bis(2-(2-methoxyethoxy)ethyl) (methylenebis(4,1-phenylene))dicarbamate 1c

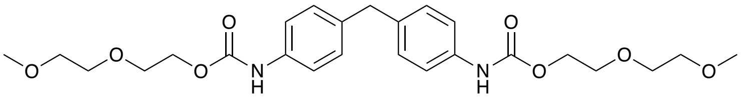

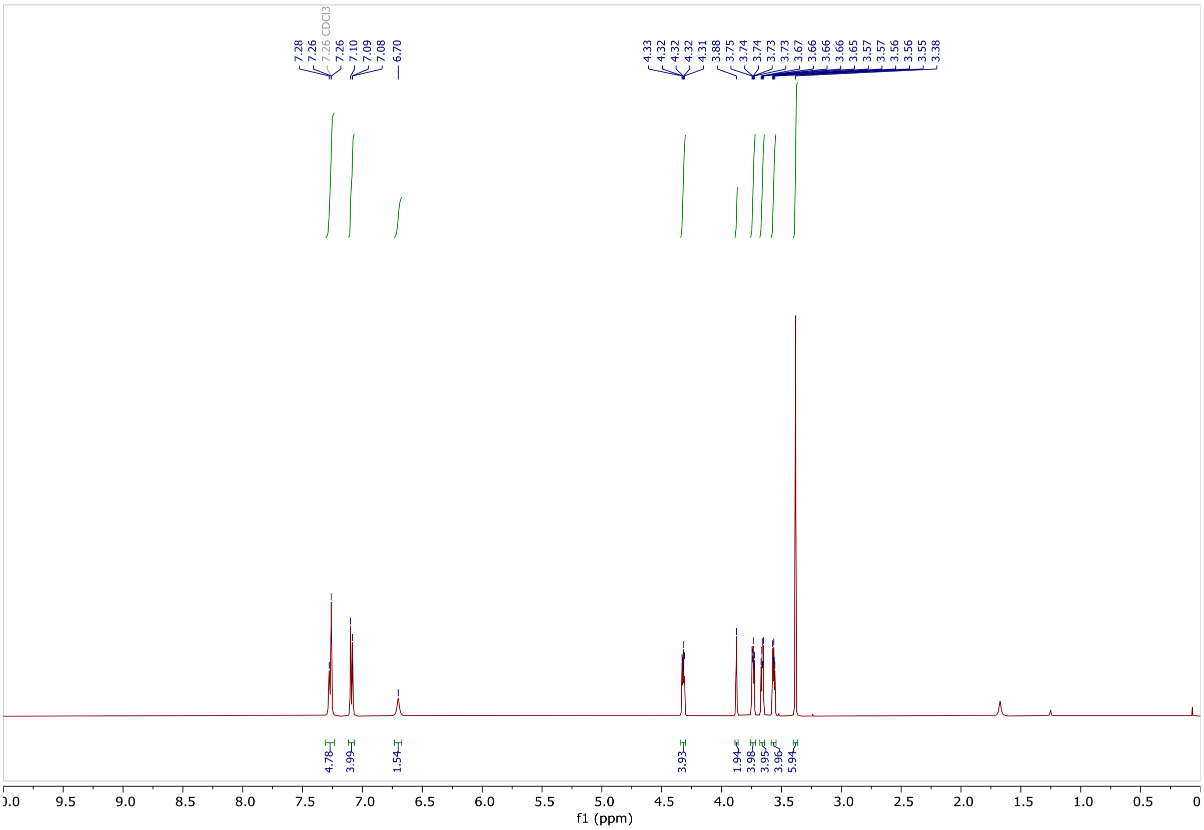

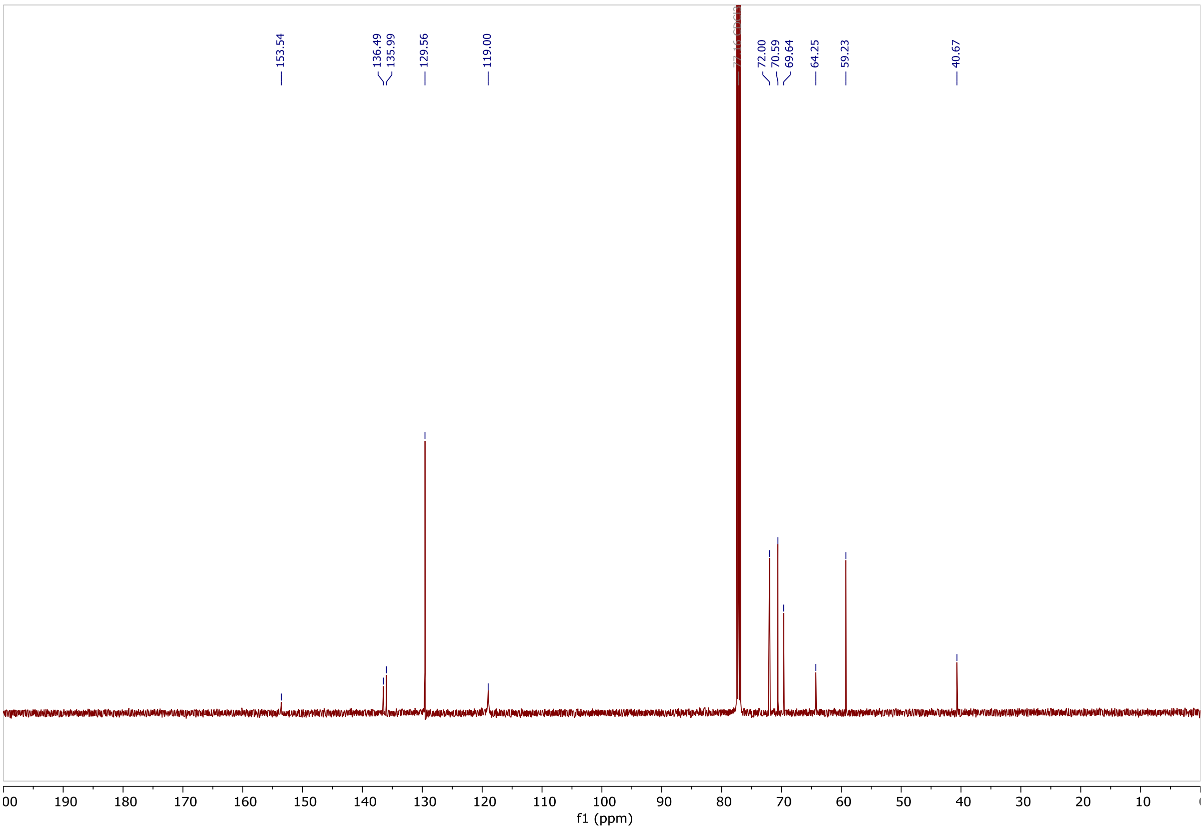

2.5.4 Bis(2-(2-(2-hydroxyethoxy)ethoxy)ethyl) (methylenebis(4,1-phenylene))dicarbamate 1d

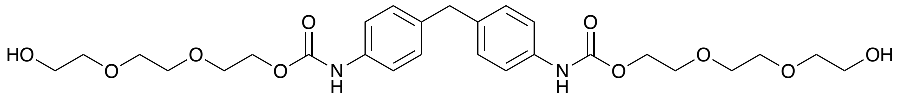

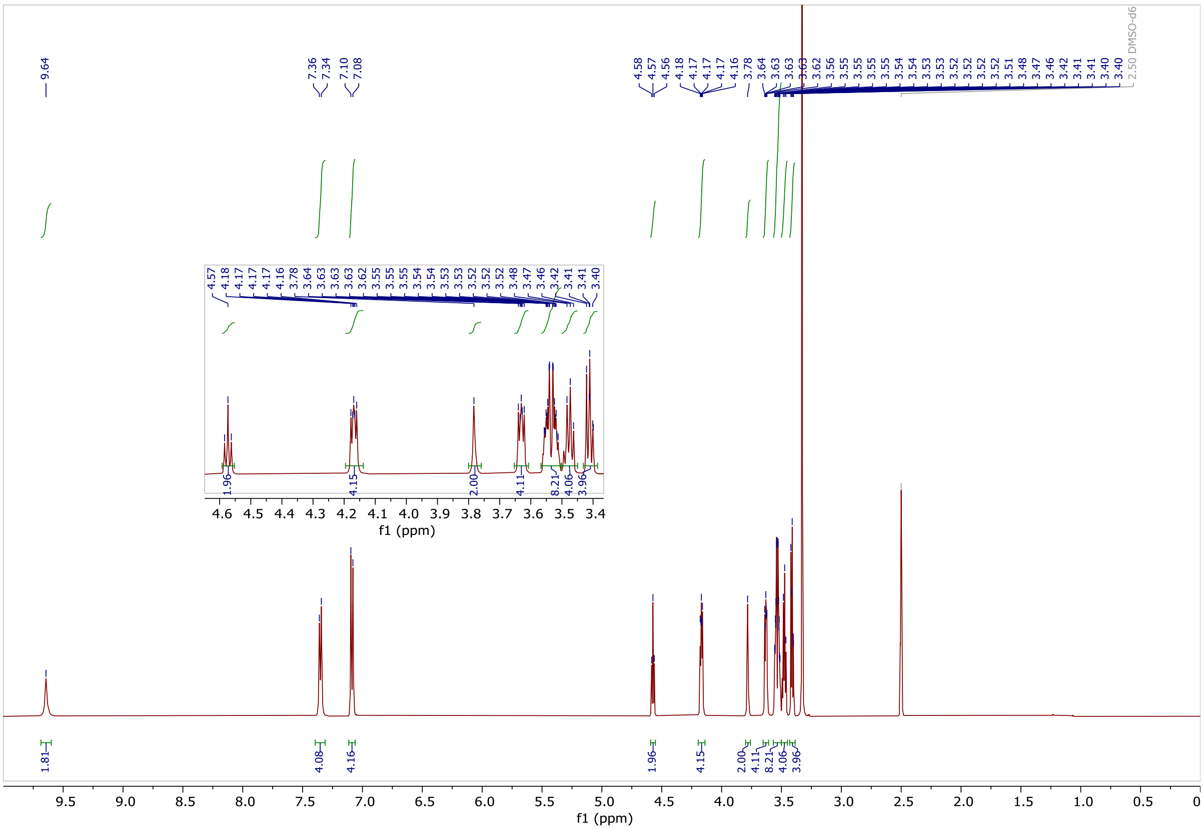

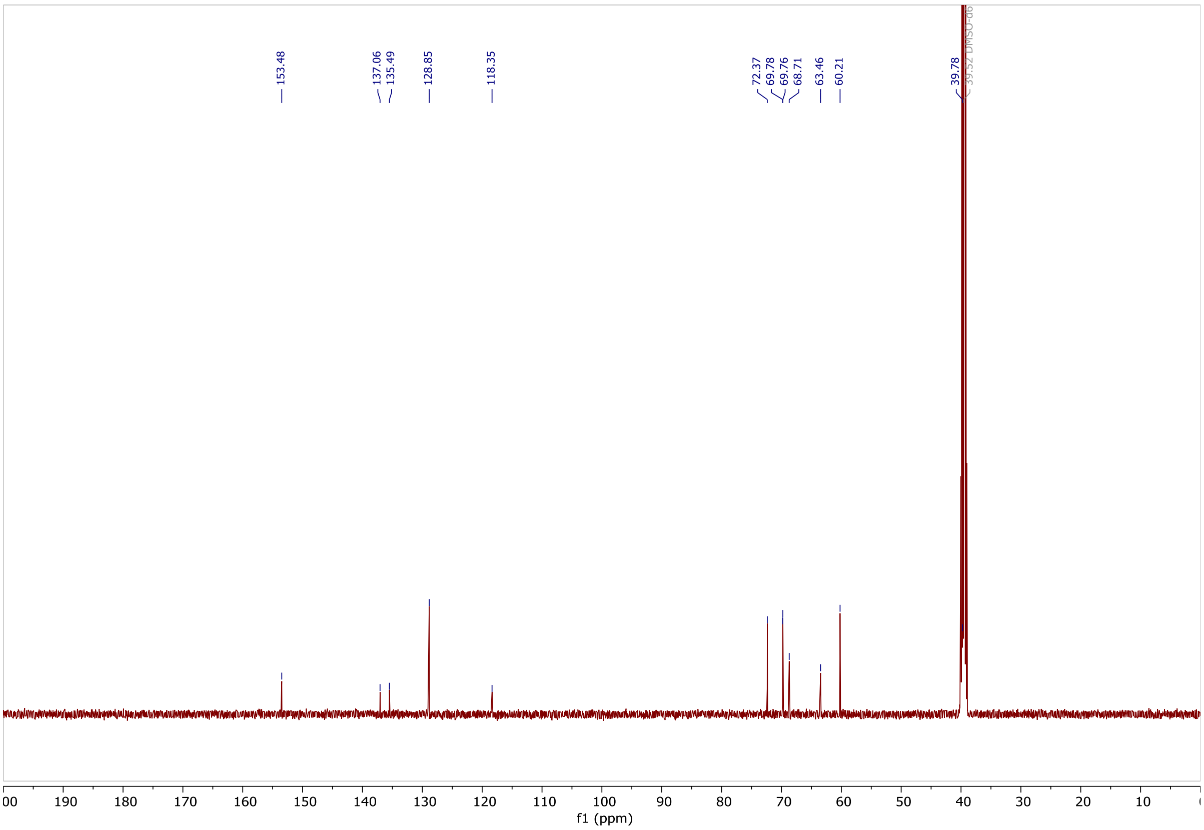

2.5.5 Diethyl (methylenebis(4,1-phenylene))dicarbamate 1e

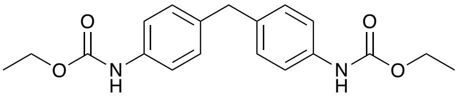

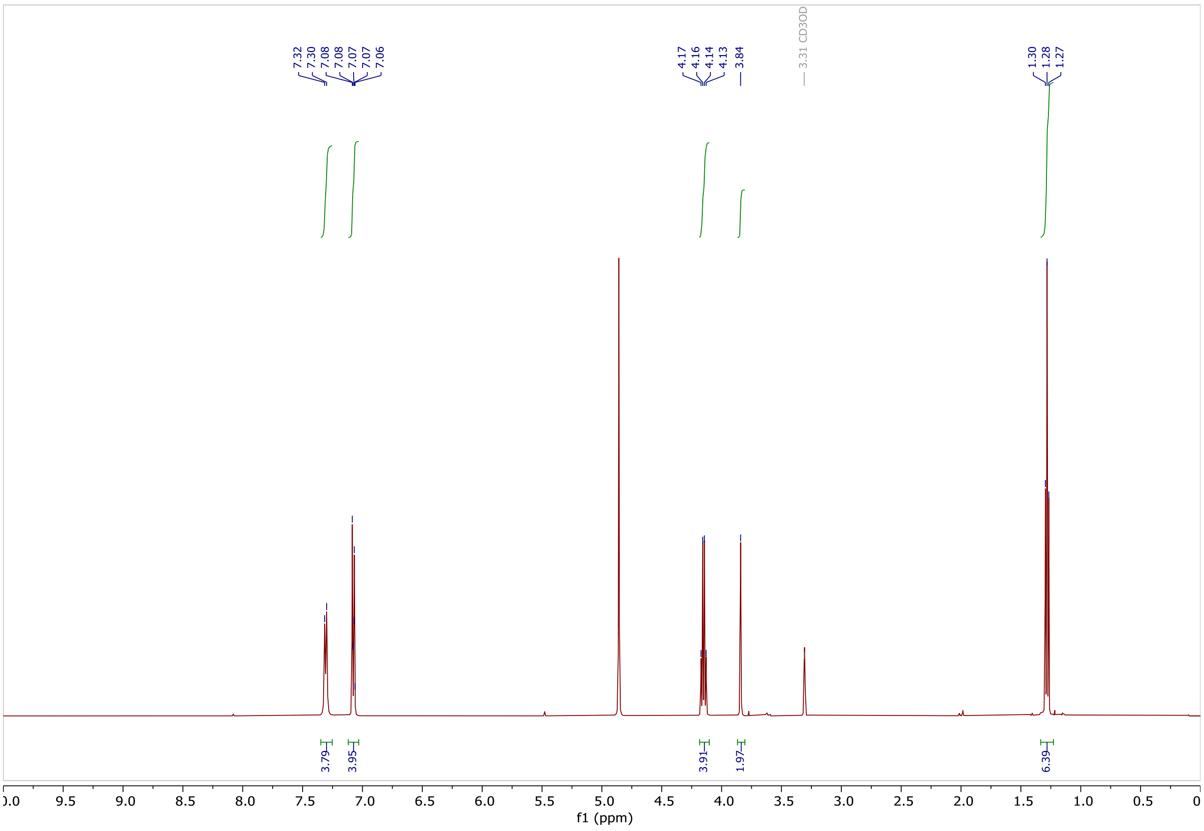

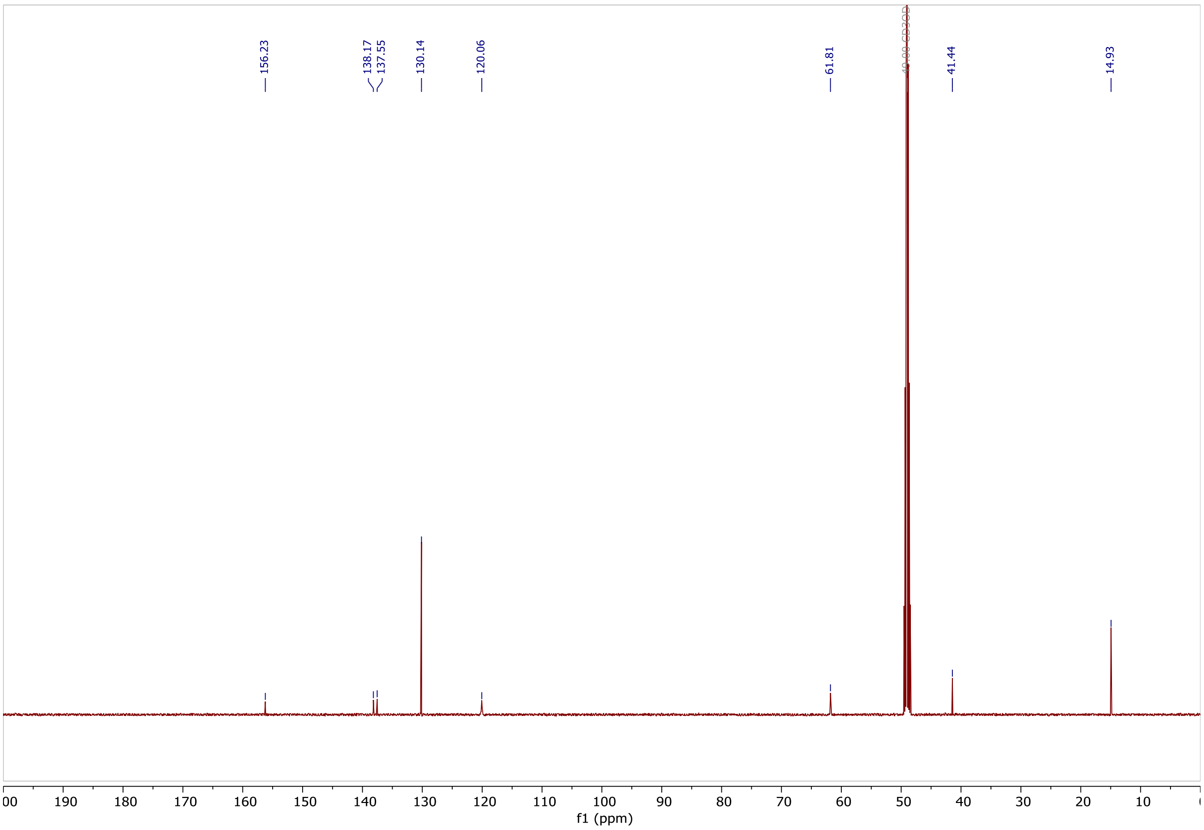

2.5.6 Dihexyl (methylenebis(4,1-phenylene))dicarbamate 1f

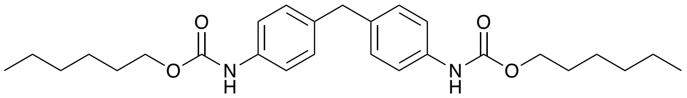

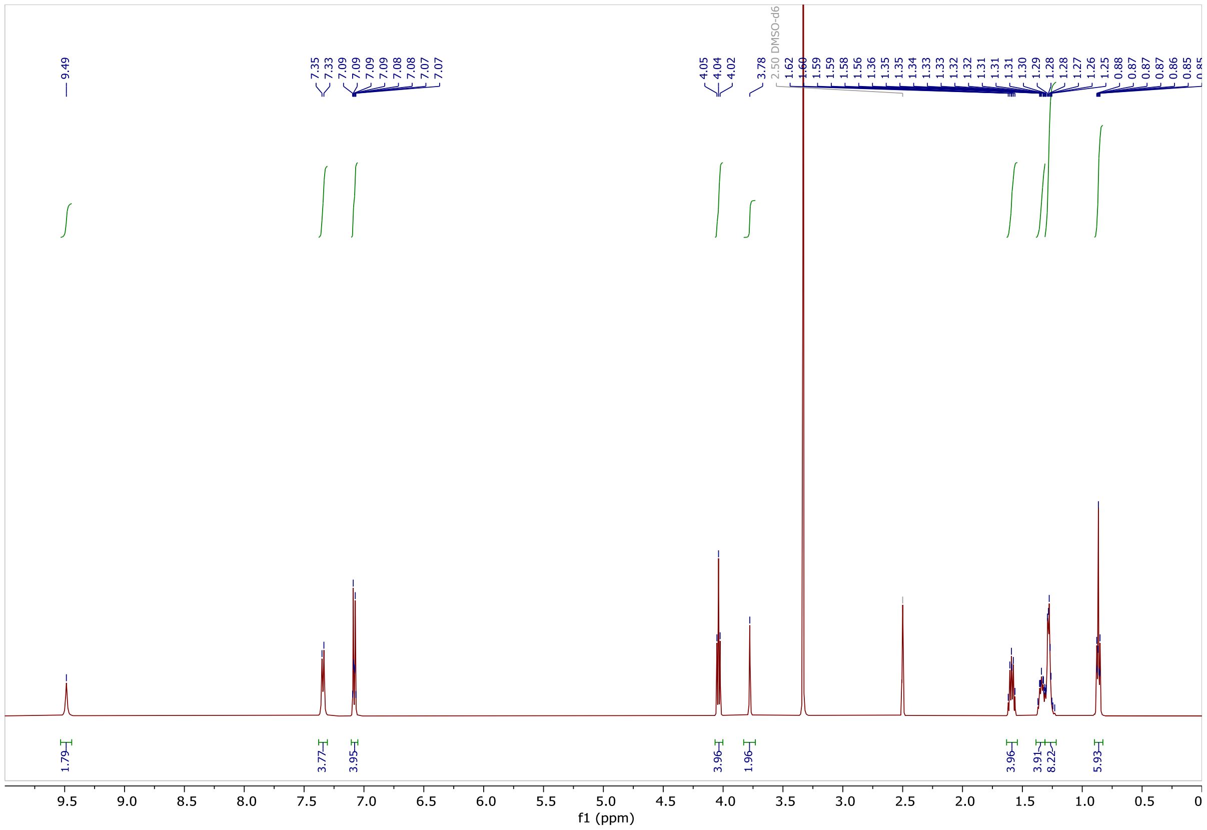

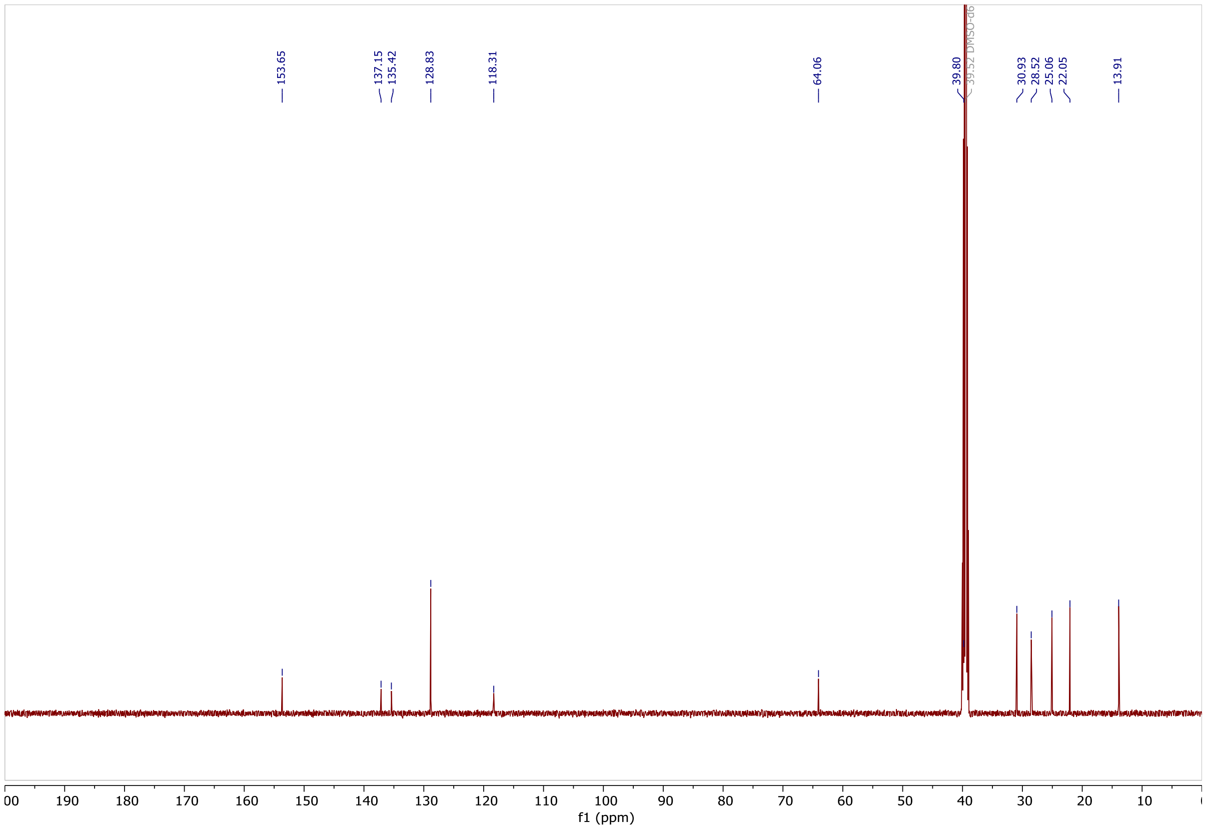

2.6 Mass spectrometry analysis of monohydrolysed compounds 2

The following LC-MS spectra were obtained from the crude enzymatic reaction with pQR3139 and substrates **1b-d** to demonstrate the formation of the mono-hydrolysed products **2**. The reactions were quenched with MeOH and filtered with a 0.45 µm syringe filter. Spectra were obtained by electrospray ionisation methods in the positive mode (ESI+). Conversion to mono-hydrolysed products **2** was calculated by finding the difference between the remaining starting material **1** and the concentration of MDA using the calibration curves in 1.16. Green spectra show the full range 0-500 *m/z* after integrating the LC peak, while the red spectra show the integration of the peak of interest in the green one. The *m/z* of each product **2** is indicated.

2.6.1 2-(2-Hydroxyethoxy)ethyl (4-(4-aminobenzyl)phenyl)carbamate 2b

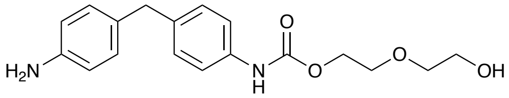

Expected *m/z* $[M+H]^+$: 331.1658; found 331.4

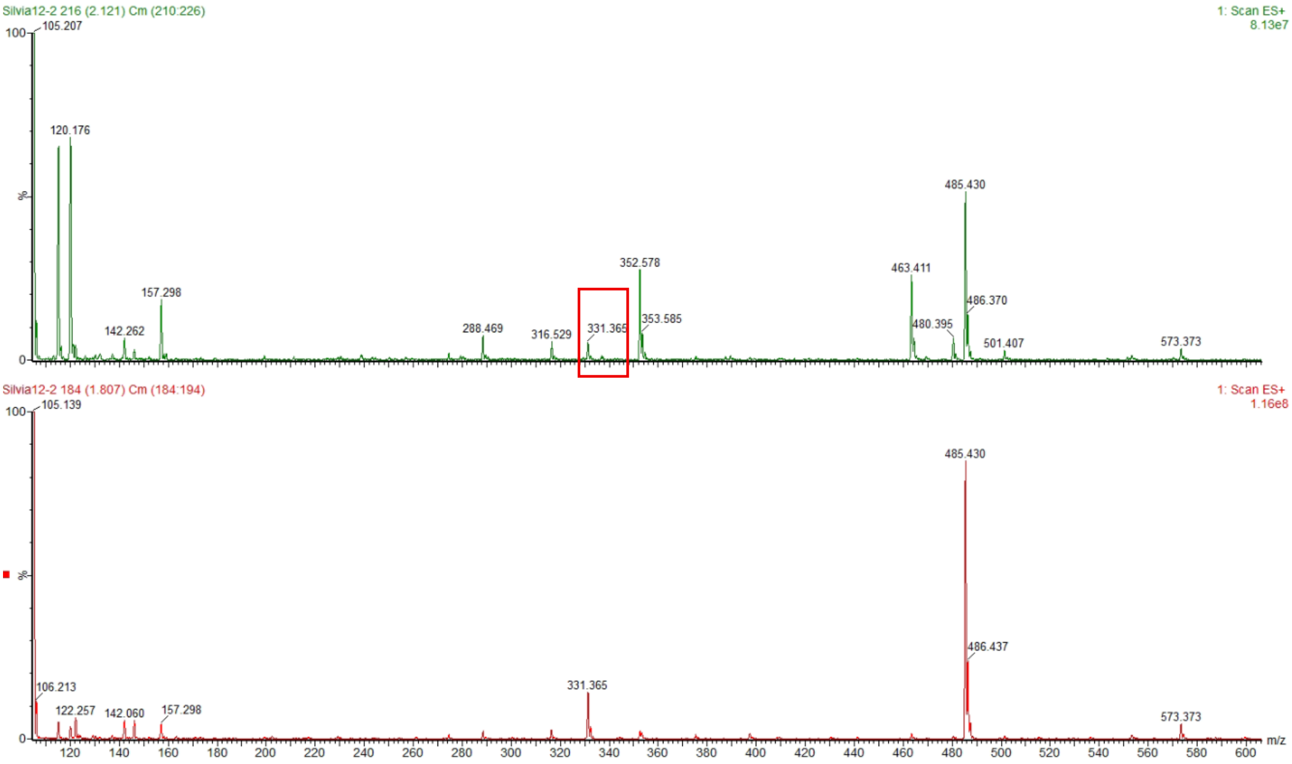

2.6.2 2-(2-Methoxyethoxy)ethyl (4-(4-aminobenzyl)phenyl)carbamate 2c

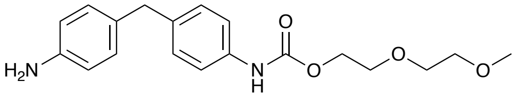

Expected *m/z* $[M+H]^+$: 345.1814; found 345.3

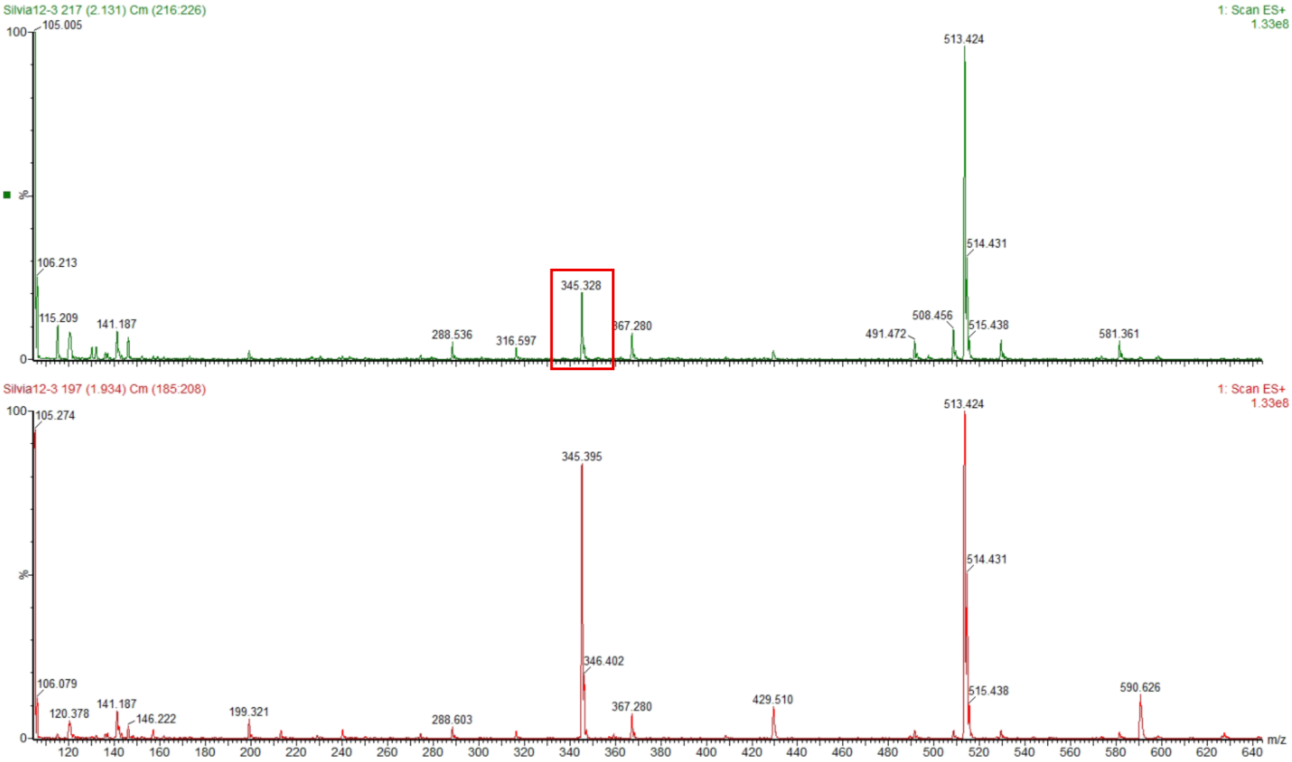

2.6.3 2-(2-(2-Hydroxyethoxy)ethoxy)ethyl (4-(4-aminobenzyl)phenyl)carbamate 2d

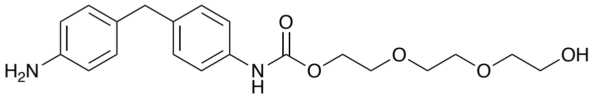

Expected *m/z* $[M+H]^+$: 375.192; found 375.3.

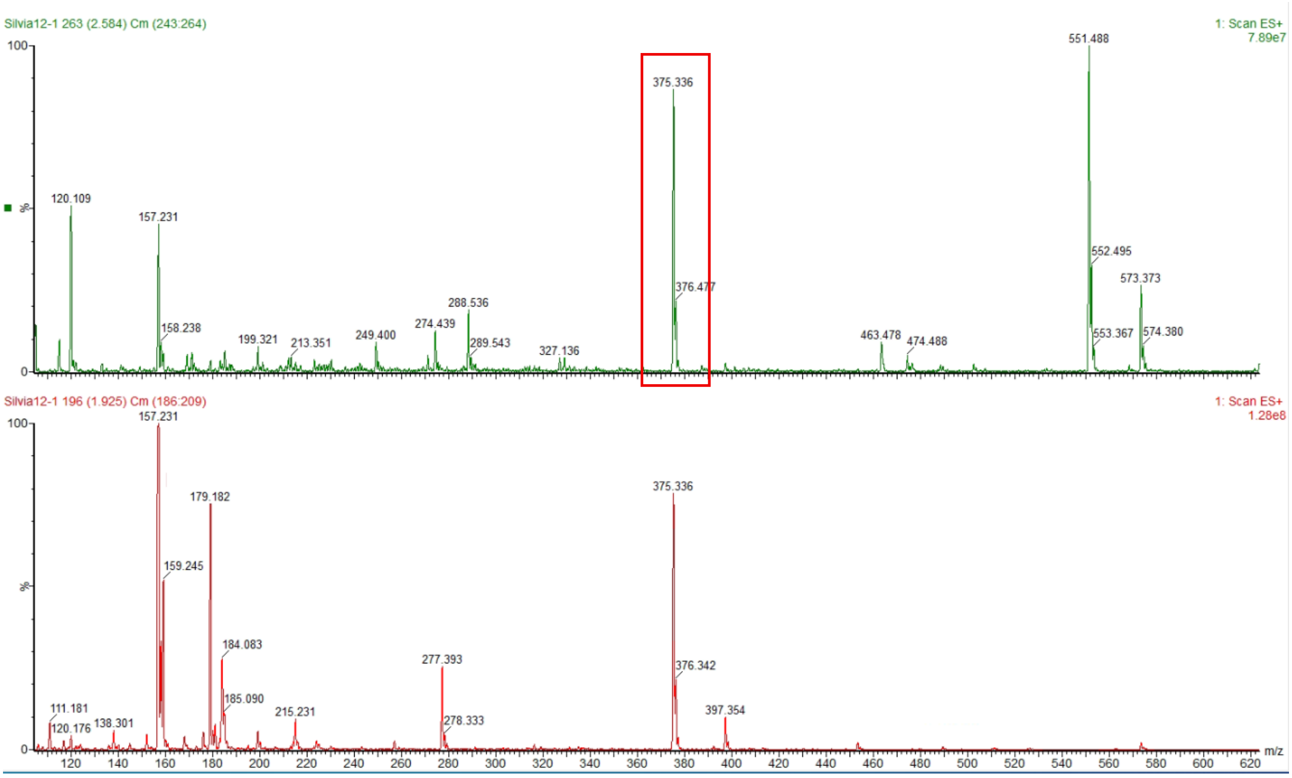

3 Molecular docking

Protein structure preparation: The structural models of pQR3139 were built using AlphaFold2[24]. The protein pdb file was opened using AutoDock Tools (v.1.5.6)[25] and hydrogens were added to the protein. The box size for ligand docking was set to cover the whole protein (CENTERX = 0, CENTERY = 0, CENTERZ = 0, docking box dimension of 60*60*60 Å). The file was then saved as a pdbqt file.

Ligand preparation: Chemdraw3D was used to generate and minimise the energy of the ligand. Then, the ligand was formatted as pdbqt file via AutoDock Tools.

Docking was performed via Autodock Vina (v.1.2.0)[26,27] through a terminal window.

Table S4. Docking analysis for pQR3139.

Ligand	ΔG (kcal/mol)	Ranking[a]	In catalytic site
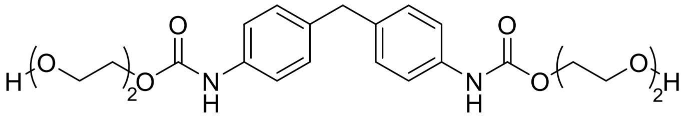 **1b**	-6.5	1	Yes
	-6.3	2	Yes
	-6.2	3	No
	-6.2	4	No
	-6.2	5	No
	-6.2	6	No
	-6.2	7	No
	-6.0	8	No
	-6.0	9	No
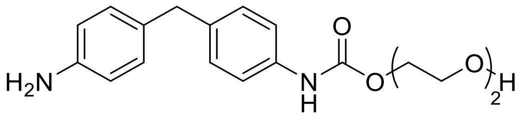 **2b**	-7.0	1	Yes
	-6.7	2	Yes
	-6.5	3	No
	-6.4	4	Yes
	-5.9	5	No
	-5.8	6	No
	-5.8	7	No
	-5.7	8	No
	-5.6	9	No
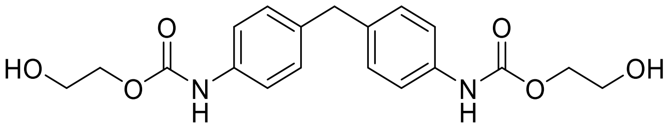 **1a**	-7.8	1	Yes
	-7.8	2	Yes
	-7.6	3	Yes
	-7.4	4	Yes
	-7.4	5	Yes
	-7.3	6	Yes
	-7.2	7	No
	-6.9	8	No
	-6.8	9	No
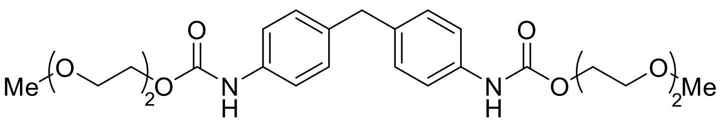 **1c**	-7.4	1	Yes
	-6.5	2	Yes
	-6.0	3	No
	-5.9	4	No
	-5.8	5	No
	-5.7	6	No
	-5.7	7	No
	-5.7	8	No
	-5.7	9	Yes

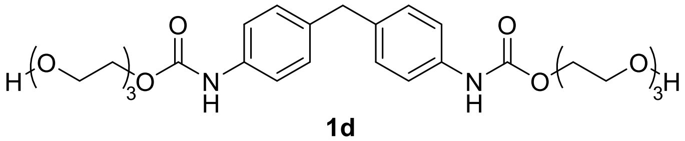 1d	-7.5	1	Yes
	-7.2	2	Yes
	-7.2	3	Yes
	-6.4	4	Yes
	-6.3	5	Yes
	-5.9	6	Yes
	-5.8	7	No
	-5.5	8	No
	-5.5	9	No

[a] The ranking order followed the affinity energy ΔG.

4 Experiments with commercial fabrics

Materials. Two fabrics were purchased: Premium Quality Viscose 4 Way Stretch Soft Jersey Fabric Rayon Material (95% viscose/5% elastane) by FABRIQUES and Cotton Lycra Elastane Jersey Fabric | Soft & Breathable 4 Way Stretch Material (96% cotton/4% elastane) by Tia Knight.

4.1 General procedures

Washing: The fabrics were soaked in MeOH overnight, then further rinsed with MeOH and water. The fabrics were then dried to obtain the clean fabric samples.

Bead-milling: 250 mg pre-washed fabrics were cut into small pieces (~ 0.5 cm x 0.5 cm), mixed with 1 mL MeOH in the jars, along with 5 x 5 mm stainless steel balls. The fabrics were milled for 1 h with 0.5 h cooling, for 4 cycles, at 30 Hz. The resulting materials were washed out with MeOH. The solid residues were washed and dried on the vacuum filter for further study. The liquid layer was combined and concentrated, then resuspended in DMSO (1-2 mL, 100 mg/mL final conc.) for further study.

Amidase reactions: 10 mg of solid fabric residues from bead-milling were mixed in 100 µL DMSO, 800 µL KPi buffer (50 mM, pH 7.5) and 100 µL amidase (0.2-0.3 mg/mL final conc. for purified enzymes or 0.6-1 mg/mL final conc. for CCL) were added. The reaction was incubated at 37 °C and 950 rpm in a thermomixer for 24-72 h.

***Rs*TYR tandem reactions**: To a 160 µL of amidase reaction results, was added 20 µL *Rs*TYR (purified, ~0.2 mg/mL final conc.) and 20 µL $CuSO_4$ (100 µM, in water). The reaction was incubated at 37 °C and 950 rpm in a thermomixer for 24 h.

4.2 Investigations with commercial fabrics

For degradation assays with the elastane fabrics, the general procedure in 4.1 was used. Assays were performed after 0 h, 1 h, 24 h and 72 h and the 96-well plates are shown below together with an empty vector control (EVC). Fractions from the bead-milling were also treated with KPi buffer, rather than amidases, and then *Rs*TYR (Figure S7, 12th lane) for the colorimetric readout.

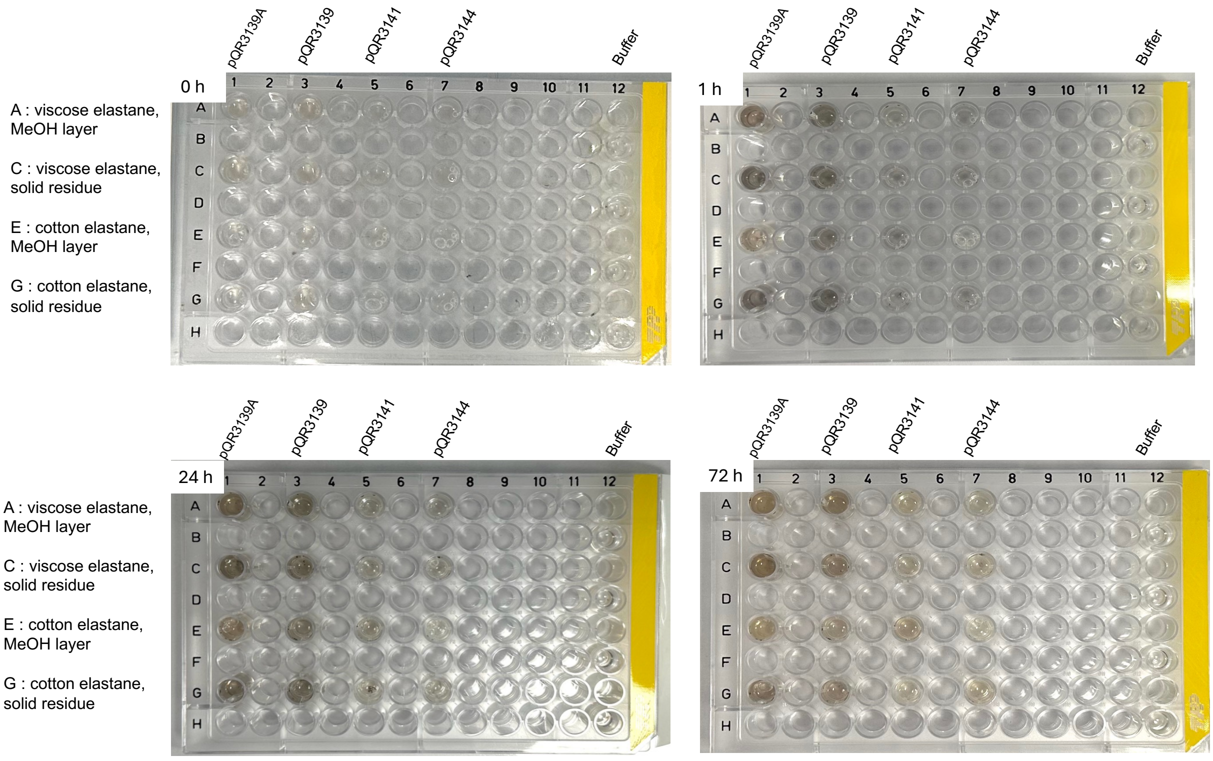

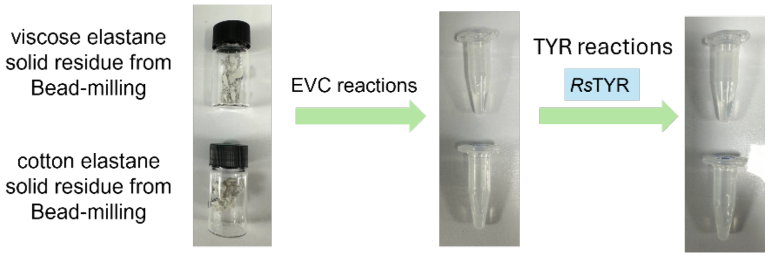

Figure S7. *TYR colorimetric assay of amidases (pQR3139, pQR3141, pQR3144) on fabrics (95% viscose/5% elastane and 96% cotton/4% elastane) in 96-well plates and the empty vector negative control (EVC) was carried out in Eppendorf tubes (EVC reactions: 10 mg materials in 100 µL DMSO, 100 µL lysates of pET-29a(+) empty vector, 800 µL KPi buffer (50 mM, pH 7.5), 37 °C, 48 h; TYR reactions: 160 µL EVC reaction results, 20 µL RsTYR, 20 µL $CuSO_4$, 37 °C, 24 h).*

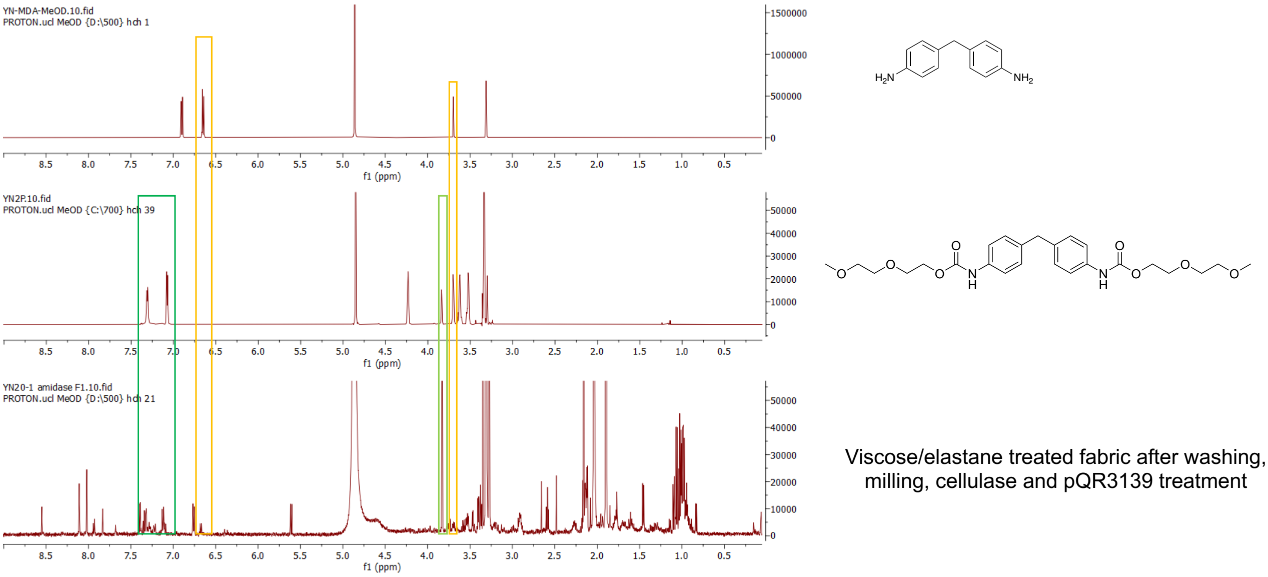

Figure S8. *^{1}H NMR spectroscopic data showing MDA (top),* ***1c*** *(middle), and the viscose/elastane fraction after washing, bead-milling, treatment with cellulase, and then pQR3139. The MDA fragments and key signals are highlighted in yellow and green. The NMR spectroscopic data showed proton signals corresponding to aromatic rings and methylene bridges from the treated fabrics, within the chemical shift range observed for model compound* ***1c*** *(marked in green). Some small peaks attributable to MDA were also detected (marked in yellow).*

5 References

1 X. Robert and P. Gouet, *Nucleic Acids Res.*, 2014, **42**, W320–W324.

2 F. Madeira, N. Madhusoodanan, J. Lee, A. Eusebi, A. Niewielska, A. R. N. Tivey, R. Lopez and S. Butcher, *Nucleic Acids Res*., 2024, **52**, W521-W525.

3 K. Katoh. K. Misawa, K. Kuma and T. Miyata, *Nucleic Acids Res*., 2002, **30**, 3059-3066.

4 L. Zimmermann, A. Stephens, S. Z. Nam, D. Rau, J. Kübler, M. Lozajic, F. Gabler, J. Söding and A. N. Lupas and V. Alva, *J. Mol. Biol.,* 2018, **430**, 2237-2243.

5 F. Gabler, S. Z. Nam, S. Till, M. Mirdita, M. Steinegger, J. Söding, A. N. Lupas and V. Alva, *Curr. Prot. Bioinform.*, 2020, **72**, e108.

6 B. Q. Minh, H. A. Schmidt, O. Chernomor, D. Schrempf, M. D. Woodhams, A. von Haeseler and R. Lanfear, *Mol. Biol. Evol*., 2020, **37**, 1530-1534.

7 S. Kalyaanamoorthy, B. Q. Minh, T. K. F. Wong, A. von Haeseler and L. S. Jermiin, *Nat. Methods*, 2017, **14**, 587-589.

8 G. Bianchini and P. Sánchez-Baracaldo, *Ecol. & Evol*., 2024, 14, e10873.

9 Y. Wang Y, N. Tappertzhofen, D. Méndez-Sánchez, M. Bawn, B. Ly, J. M. Ward and H. C. Hailes, *Angew. Chem. Int. Ed*., 2019, **58**, 10120-10125.

10 Y. Wang, F. Subrizi, E. M. Carter, T. D. Sheppard, J. M. Ward and H. C. Hailes, *Nat. Commun*., 2022, **13**, 5436.

11 S. Yoshida, K. Hiraga, T. Takehana, I. Taniguchi, H. Yamaji, Y. Maeda, K. Toyohara, K. Miyamoto, Y. Kimura and K. Oda, *Science,* 2016, **351**, 1196-1199.

12 H. P. Austin, M. D. Allen, B. S. Donohoe, N. A. Rorrer, F. L. Kearns, R. L. Silveira, B. C. Pollard, G. Dominick, R. Duman, K. El Omari, V. Mykhaylyk, A. Wagner, W. E. Michener, A. Amore, M. S. Skaf, M. F. Crowley, A. W. Thorne, C. W. Johnson, H. Lee Woodcock, J. E. McGeehan and G. T. Beckham, *Proc. Nat. Acad. Sci. U S A*, 2018, **115**, E4350–E4357.

13 E. Ambrose-Dempster, L. Leipold, D. Dobrijevic, M. Bawn, E. M. Carter, G. Stojanovski, T. D. Sheppard, J. W. E. Jeffries, J. M. Ward and H. C. Hailes, *RSC Adv.*, 2023, **13**, 9954-9962.

14 D. Danso, J. Chow and W. R. Streita, *Appl. Environ. Microbiol,*, 2019, **85**, e01095-19.

15 H. F. Son, I. J. Cho, S. Joo, H. Seo, H. Y. Sagong, S. Y. Choi, S. Y. Lee and K. J. Kim, *ACS Catal*, 2019, **9**, 3519-3526.

16 V. Tournier, C. M. Topham, A. Gilles, B. David, C. Folgoas, E. Moya-Leclair, E. Kamionka, M.-L. Desrousseaux, H. Texier, S. Gavalda, M. Cot, E. Guémard, M. Dalibey, J. Nomme, G. Cioci, S. Barbe, M. Chateau, I. André, S. Duquesne and A. Marty, *Nature*, 2020, **580**, 216-219.

17 Y. Cui, Y. Chen, X. Liu, S. Dong, Y. Tian, Y. Qiao, R. Mitra, J. Han, C. Li, X. Han, W. Liu, Q. Chen, W. Wei, X. Wang, W. Du, S. Tang, H. Xiang, H. Liu, Y. Liang, K. N. Houk and B. Wu, *ACS Catal.*, 2021, **11**, 1340-1350.

18 E. L. Bell, R. Smithson, S. Kilbride, J. Foster, F. J. Hardy, S. Ramachandran, A. A. Tedstone, S. J. Haigh, A. A. Garforth, P. J. R. Day, C. Levy, M. P. Shaver and A. P. Green, *Nat. Catal.*, 2022, **5**, 673-681.

19 H. Lu, D. J. Diaz, N. J. Czarnecki, C. Zhu, W. Kim, R. Shroff, D. J. Acosta, B. R. Alexander, H. O. Cole, Y. Zhang, N. A. Lynd, A. D. Ellington and H. S. Alper, *Nature,* 2022, **604**, 662-667.

20 O. Chertkov, J. Sikorski, M. Nolan, A. Lapidus, S. Lucas, T. G. del Rio, H. Tice, J. F. Cheng, L. Goodwin, S. Pitluck, K. Liolios, N. Ivanova, K. Mavromatis, N. Mikhailova, G. Ovchinnikova, A. Pati, A. Chen, K. Palaniappan, O. D. N. Djao, M. Land, L. Hauser, Y. J. Chang, C. D. Jeffries, T. Brettin, C. Han, J. C. Detter, M. Rohde, M. Göker, T. Woyke, J. Bristow, J. A. Eisen, V. Markowitz, P. Hugenholtz, H. P. Klenk and N. C. Kyrpides, *Stand. Genomic Sci.*, 2011, **4**, 13-22.

21 Y. Branson, S. Söltl, C. Buchmann, R. Wei, L. Schaffert, C. P. S. Badenhorst, L. Reisky, G. Jäger and U. T. Bornscheuer, *Angew. Chem. Int. Ed.*, 2023, **62**, e202216220.

22 R. Muranaka, Y. Liu, I. Okada, T. Okazoe and A. Tsuda, *ACS Omega*, 2022, **7**, 5584-5594.

23 Z. Wang, X. Yan, N. Ma, S. Liu, P. Han, H. Li, Q. Mahmood, L. Li and Q. Liu, *J. Catal.*, 2023, **428**, 115165.

24 J. Jumper, R. Evans, A. Pritzel, T. Green, M. Figurnov, O. Ronneberger, K. Tunyasuvunakool, R. Bates, A. Žídek, A. Potapenko, A. Bridgland, C. Meyer, S. A. A. Kohl, A. J. Ballard, A. Cowie, B. Romera-Paredes, S. Nikolov, R. Jain, J. Adler, T. Back, S. Petersen, D. Reiman, E. Clancy, M. Zielinski, M. Steinegger, M. Pacholska, T. Berghammer, S. Bodenstein, D. Silver, O. Vinyals, A. W. Senior, K. Kavukcuoglu, P. Kohli and D. Hassabis, *Nature*, 2021, **596**, 583-589.

25 G. M. Morris, R. Huey, W. Lindstrom, M. F. Sanner, R. K. Belew, D. S. Goodsell and A. J. Olson, *J. Comput. Chem.*, 2009, **30**, 2785-2791.

26 O. Trott and A. Olson, *J. Comp. Chem.,* 2010, **31**, 455-461.

27 J. Eberhardt, D. Santos-Martins, A. F. Tillack and S. Forli, *J. Chem. Inf. Mod*el., 2021, **61**, 3891-3898.
